# RNAseq Analysis of the Parasitic Nematode *Strongyloides stercoralis* Reveals Divergent Regulation of Canonical Dauer Pathways

**DOI:** 10.1371/journal.pntd.0001854

**Published:** 2012-10-25

**Authors:** Jonathan D. Stoltzfus, Samuel Minot, Matthew Berriman, Thomas J. Nolan, James B. Lok

**Affiliations:** 1 Department of Pathobiology, University of Pennsylvania School of Veterinary Medicine, Philadelphia, Pennsylvania, United States of America; 2 Department of Microbiology, Perelman School of Medicine at the University of Pennsylvania, Philadelphia, Pennsylvania, United States of America; 3 Wellcome Trust Sanger Institute, Wellcome Trust Genome Campus, Hinxton, Cambridge, United Kingdom; University of Melbourne, Australia

## Abstract

The infectious form of many parasitic nematodes, which afflict over one billion people globally, is a developmentally arrested third-stage larva (L3i). The parasitic nematode *Strongyloides stercoralis* differs from other nematode species that infect humans, in that its life cycle includes both parasitic and free-living forms, which can be leveraged to investigate the mechanisms of L3i arrest and activation. The free-living nematode *Caenorhabditis elegans* has a similar developmentally arrested larval form, the dauer, whose formation is controlled by four pathways: cyclic GMP (cGMP) signaling, insulin/IGF-1-like signaling (IIS), transforming growth factor β (TGFβ) signaling, and biosynthesis of dafachronic acid (DA) ligands that regulate a nuclear hormone receptor. We hypothesized that homologous pathways are present in *S. stercoralis*, have similar developmental regulation, and are involved in L3i arrest and activation. To test this, we undertook a deep-sequencing study of the polyadenylated transcriptome, generating over 2.3 billion paired-end reads from seven developmental stages. We constructed developmental expression profiles for *S. stercoralis* homologs of *C. elegans* dauer genes identified by BLAST searches of the *S. stercoralis* genome as well as *de novo* assembled transcripts. Intriguingly, genes encoding cGMP pathway components were coordinately up-regulated in L3i. In comparison to *C. elegans*, *S. stercoralis* has a paucity of genes encoding IIS ligands, several of which have abundance profiles suggesting involvement in L3i development. We also identified seven *S. stercoralis* genes encoding homologs of the single *C. elegans* dauer regulatory TGFβ ligand, three of which are only expressed in L3i. Putative DA biosynthetic genes did not appear to be coordinately regulated in L3i development. Our data suggest that while dauer pathway genes are present in *S. stercoralis* and may play a role in L3i development, there are significant differences between the two species. Understanding the mechanisms governing L3i development may lead to novel treatment and control strategies.

## Author Summary

Parasitic nematodes infect over one billion people worldwide and cause many diseases, including strongyloidiasis, filariasis, and hookworm disease. For many of these parasites, including *Strongyloides stercoralis*, the infectious form is a developmentally arrested and long-lived thirdstage larva (L3i). Upon encountering a host, L3i quickly resume development and mature into parasitic adults. In the free-living nematode *Caenorhabditis elegans*, a similar developmentally arrested third-stage larva, known as the dauer, is regulated by four key cellular mechanisms. We hypothesized that similar cellular mechanisms control L3i arrest and activation. Therefore, we used deep-sequencing technology to characterize the *S. stercoralis* transcriptome (RNAseq), which allowed us to identify *S. stercoralis* homologs of components of these four mechanisms and examine their temporal regulation. We found similar temporal regulation between *S. stercoralis* and *C. elegans* for components of two mechanisms, but dissimilar temporal regulation for two others, suggesting conserved as well as novel modes of developmental regulation for L3i. Understanding L3i development may lead to novel control strategies as well as new treatments for strongyloidiasis and other diseases caused by parasitic nematodes.

## Introduction

Parasitic nematodes infect over one billion people worldwide, resulting in vast morbidity [1], as well as causing significant agricultural losses from infections of both animals and plants [2]. The infectious form of many parasitic nematodes, including those causing hookworm disease, filariasis, and strongyloidiasis, is a developmentally arrested third-stage larva (L3i), which is both stress-resistant and long-lived [3]–[5]. Upon entering a suitable host, L3i quickly resume development (activation), eventually forming parasitic adults [4], [5]. The genes and proteins constituting the pathways that control the developmental arrest and activation of L3i represent potential targets for chemotherapy as well as environmental control strategies.

**Figure 1:**
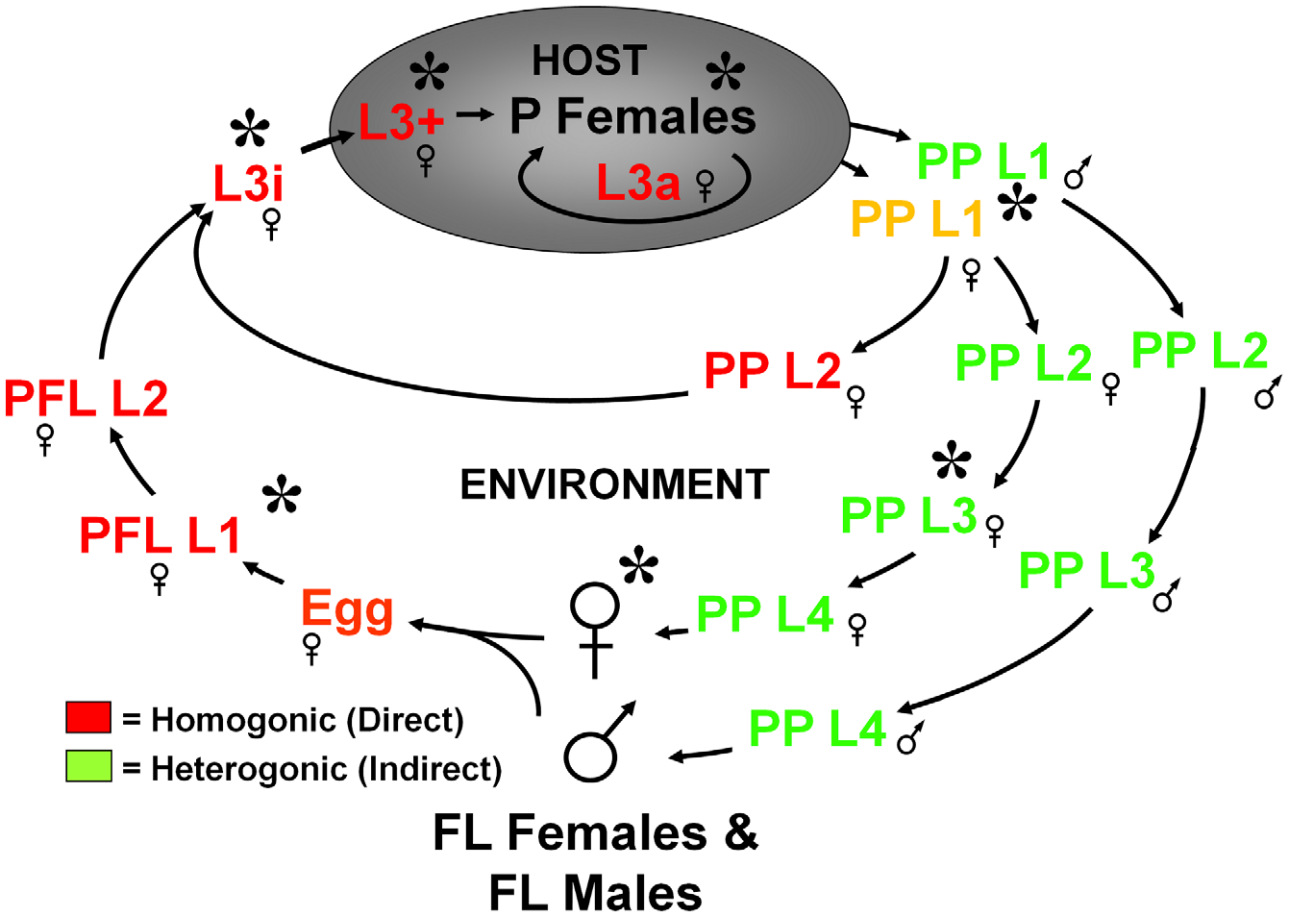
Diagram of the *Strongyloides stercoralis* life cycle. Developmentally arrested infective third-stage larvae (L3i) can form by either a homogonic route (dark red) or a heterogonic route (light green). Female post-parasitic first-stage larvae (PP L1) passed in the feces of the infected host can develop homogonically through two larval molts directly to L3i or heterogonically through four larval molts to free-living females (FL Females). Post-parasitic L1 males invariably develop heterogonically through four molts to free-living males (FL Males). Post-free-living L1 (PFL L1), which are all female, molt twice and develop exclusively to L3i. Upon encountering and penetrating a susceptible host, activated third-stage larvae (L3+) resume feeding and development, migrate to the intestines, and molt twice into parasitic females (P Females). Post-parasitic L1 larvae can also precociously develop into auto-infective third-stage larvae (L3a) entirely within the host. Developmental stages marked with an asterisk (*) were interrogated by RNAseq. Adapted from [7].

Our lab uses the parasitic nematode *Strongyloides stercoralis*, which infects 30–100 million people globally [1], to study mechanisms controlling L3i arrest and activation [6]. *S. stercoralis* has a complex life-cycle (Figure 1), which includes both an obligatory parasitic generation as well as a facultative free-living generation. Parasitic females reproduce parthenogenetically to produce post-parasitic larvae, which develop either directly to L3i (homogonic/direct development) or to free-living males and females (heterogonic/indirect development). Post-free-living larvae constitutively form L3i [7]. This life cycle allows us to investigate the mechanisms underlying different developmental fates for similar larval forms. Additionally, we have developed molecular tools in *S. stercoralis*, which are unavailable in other parasitic nematodes, to investigate molecular mechanisms involved in L3i regulation [8]–[10].

**Figure 2:**
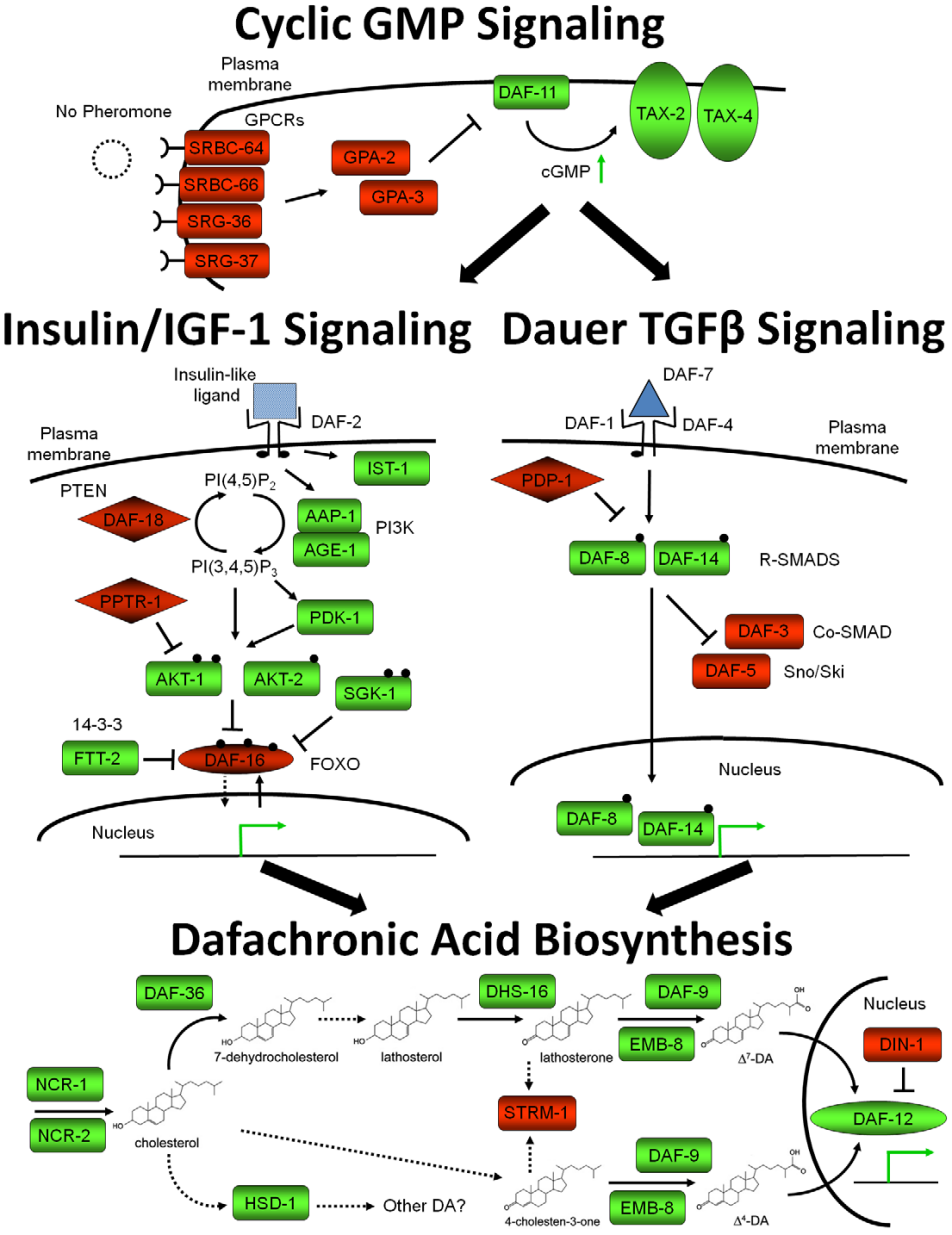
*Caenorhabditis elegans* dauer pathways during reproductive development. Four developmental pathways regulate *C. elegans* dauer entry and exit: a cyclic guanosine monophosphate (GMP) signaling pathway, an insulin/insulin-like growth factor 1 (IGF-1) -like signaling pathway, a dauer transforming growth factor β (TGFβ) pathway, and a nuclear hormone receptor (DAF-12) regulated by a class of steroid ligands known as dafachronic acids (DAs). This simplified model depicts the four pathways under conditions favoring reproductive development and repression of dauer arrest. Proteins in light green are “active,” while proteins in dark red are “inactive.” Black circles represent phosphorylation sites and diamond-shaped boxes represent phosphatases. Green arrows represent either increases in metabolite concentration or increases in gene transcription. Solid black lines represent well-established pathways, while dashed lines represent putative pathways. Adapted from [12], [54], [111], [129].

The free-living nematode *Caenorhabditis elegans* has a developmentally arrested third-stage dauer larva, morphologically similar to L3i, which forms during conditions of low food abundance, high temperature, and high dauer pheromone levels reflecting high population density. Dauer larvae quickly resume development into reproductive adults once environmental conditions improve. Mutant screens in *C. elegans* have identified over 30 genes that are involved in dauer formation (*daf*), and mutations in these genes result in either dauer constitutive (daf-c) or dauer defective (daf-d) phenotypes. Extensive study has placed many of these *daf* genes into four dauer pathways (Figure 2): a cyclic guanosine monophosphate (cGMP) signaling pathway, an insulin/IGF-1-like signaling (IIS) pathway regulated by insulin-like peptide (ILP) ligands, a dauer transforming growth factor β (TGFβ) pathway regulated by the *Ce*-DAF-7 ligand, and a nuclear hormone receptor (NHR) regulated by a class of steroid ligands known as dafachronic acids (DAs) [11]. Epistatic analysis places the cGMP signaling pathway upstream of the parallel IIS and dauer TGFβ pathways, which converge on the DA biosynthetic pathway, ultimately regulating the NHR *Ce*-DAF-12 (Figure 2) [12]. A long-standing paradigm in the field, known as the “dauer hypothesis,” proposes that similar molecular mechanisms regulate the developmental arrest and activation of both *C. elegans* dauer larvae and L3i of parasitic nematodes [4], [13]–[15], despite their high degree of evolutionary divergence [16], [17].

Members from each of the four dauer pathways have been cloned in *S. stercoralis* [18]–[22]; however, it is unclear whether all members from each of the *C. elegans* pathways are present in this parasite, whether their anatomical and temporal regulation is similar to *C. elegans*, and whether they control L3i development in *S. stercoralis*. While we have demonstrated that *S. stercoralis* IIS plays a crucial role in post-free-living L3i arrest and activation [10], [22], we have also shown that an *S. stercoralis* TGFβ ligand encoding gene, *Ss-tgh-1*, is transcriptionally regulated in a manner opposite to that of the *C. elegans* TGFβ ligand encoding gene *Ce-daf-7* [20], [23]. Studies examining the global transcriptional changes during *S. stercoralis* L3i development have failed to identify specific pathways regulating L3i development and have not directly shown whether pathways regulating dauer in *C. elegans* are similarly regulated in *S. stercoralis* [24], [25]. However, these studies have been hindered by a small expressed sequence tag (EST) database, which does not include homologs for many *C. elegans* dauer genes.

To overcome these obstacles, we used a next-generation RNA sequencing (RNAseq) approach aided by the concurrent release of draft *Strongyloides ratti* and *S. stercoralis* genome sequences. Similar to recent work in other parasitic nematode species [26]–[31], we isolated polyadenylated RNA from seven different *S. stercoralis* developmental stages (Figure 1), from which we constructed dsDNA libraries that were subjected to high-throughput sequencing. Using both *S. ratti* and *S. stercoralis* genomic contigs as well as *de novo* assembled RNAseq transcripts, we identified *S. stercoralis* homologs of *C. elegans* genes involved in dauer regulation and examined their temporal regulation throughout the *S. stercoralis* life cycle using a collection of over 2.3 billion paired-end reads.

While we identified *S. stercoralis* homologs of nearly all *C. elegans* dauer genes, some of which appear to have similar developmental regulation between the two species, we also identified multiple differences between *C. elegans* dauer genes and their *S. stercoralis* homologs, including protein structure, developmental regulation, and expansion of gene families. Both IIS and cGMP signaling appear to be regulated in a manner consistent with a role in L3i regulation, while genes putatively involved in DA biosynthesis were not coordinately regulated during L3i development. *S. stercoralis* dauer-like TGFβ signaling was regulated oppositely to that observed in *C. elegans*; nevertheless, this pathway may play a unique role in *S. stercoralis* L3i development.

## Materials and Methods

### Ethics statement

The *S. stercoralis* PV001 strain was maintained in prednisolone-treated beagles in accordance with protocols 702342, 801905, and 802593 approved by the University of Pennsylvania Institutional Animal Care and Use Committee (IACUC). Experimental infections of *S. stercoralis* were conducted in Mongolian gerbils under the same IACUC-approved protocols, and animals were sacrificed by CO_2_ asphyxiation in accordance with standards established by the American Veterinary Medical Association. All IACUC protocols, as well as routine husbandry care of the animals, were carried out in strict accordance with the *Guide for the Care and Use of Laboratory Animals of the National Institutes of Health*.

### *S. stercoralis* maintenance and RNA isolation

The *S. stercoralis* PV001 line, derived from a single female worm [22], was maintained and cultured as previously described [6], [32], [33]. *S. stercoralis* developmental stages were isolated as previously described [22]; see supplemental methods for detailed protocol (Text S1). Both L3+, which had resumed development as evidenced by changes in morphology and resumption of feeding (Figure S1), and parasitic females were derived from experimental infections of Mongolian gerbils, a permissive host [33]. All developmental stages, except for parasitic females and L3+, were rendered free of fine particle debris by migration through agarose [34] into BU buffer [35]. Worms were snap-frozen in TRIzol reagent (Life Technologies, http://www.lifetechnologies.com) in liquid nitrogen; total RNA was extracted using the manufacturer's protocol. Total RNA was quantified using the Bioanalyzer 2100 (Agilent Technologies, Inc., http://www.agilent.com), and only samples with an RNA integrity number (RIN) greater than 8.0 were used.

### *S. stercoralis* polyadenylated RNA library construction and sequencing

Libraries were constructed using the TruSeq RNA Sample Preparation Kit (Illumina, Inc., http://www.illumina.com) according to the manufacturer's protocol. For each of the 21 libraries, 500 ng of total RNA, diluted to 10 ng/µl in de-ionized water, was used as starting material. Polyadenylated RNA enrichment was performed first using olido-dT beads and eluted polyadenylated RNA fragmented at 94°C for eight minutes to approximately 170±50 (standard deviation) bp. Subsequently, first and second strand cDNA was synthesized; unique adapters for each replicate were then ligated. dsDNA fragments with ligated adapters were enriched using 15 cycles of PCR. Libraries were assessed for fragment size distribution using the Bioanalyzer 2100.

The concentration of the dsDNA adapter-ligated libraries was then determined by quantitative PCR (qPCR) using the Kapa SYBR Fast qPCR Kit for Library Quantification (Kapa Biosystems, Inc., http://www.kapabiosystems.com) using the manufacturer's protocol. Three dilutions, at 1∶4,000, 1∶8,000, and 1∶16,000, were used to calculate the concentration of each of the 21 libraries using a calibration curve of Kapa standards. Each library was then diluted to 15 nM, and libraries from each developmental stage were pooled in equal volume quantities. The concentration of each of these pools was determined using qPCR and diluted to a final concentration of 10 nM.

The quality of the pooled libraries from each of the seven developmental stages was assessed using the High Sensitivity DNA Assay (Agilent Technologies). Pooled libraries were loaded on individual lanes of the Illumina HiSeq 2000 flow cell at 4 pM for all libraries, except for the post-free-living L1 and parasitic female libraries, which were loaded at 3 pM. Samples were then sequenced on the Illumina HiSeq 2000 with 100 bp paired-end reads, with image analysis and base calling performed with HiSeq Control Software. Raw flow-cell data was processed and demultiplexed using CASAVA version 1.8.2 (Illumina) for each of the 21 samples (ArrayExpress accession number E-MTAB-1164; http://www.ebi.ac.uk/arrayexpress/experiments/E-MTAB-1164).

### Alignment of *S. stercoralis* RNAseq reads to genomic contigs

Raw reads from each of the 21 samples were independently aligned to *S. stercoralis* genomic contigs (6 December 2011 draft; ftp://ftp.sanger.ac.uk/pub/pathogens/HGI/) using TopHat version 1.4.1 (http://tophat.cbcb.umd.edu/), which utilized the Bowtie aligner version 0.12.7 (http://bowtie-bio.sourceforge.net/index.shtml) and SAMtools version 0.1.18 (http://samtools.sourceforge.net/). We refined the alignment parameters until TopHat accurately predicted introns and exons of several known *S. stercoralis* genes. Default parameters were used, but with the following options: mate inner distance of 25; mate standard deviation of 50; minimum anchor length of 6; minimum intron length of 30; maximum intron length of 20,000; micro exon search; minimum segment intron of 30; and maximum segment intron of 20,000. Aligned reads from each developmental stage were inspected using the Integrated Genome Viewer (IGV) version 2.0.34 (http://www.broadinstitute.org/igv/).

### *De novo* assembly of developmental stage-specific *S. stercoralis* transcripts

RNAseq reads from the sample with the greatest number of reads for each stage were independently *de novo* assembled into transcripts. First, forward and reverse read pairs were merged to form a single “contig” using SeqPrep (https://github.com/jstjohn/SeqPrep), with a quality score cutoff of 35, a minimum merged read length of 100 bp, and no mismatches in the overlapping region. The two read contigs were then trimmed with the FASTX toolkit quality trimmer (http://hannonlab.cshl.edu/fastx_toolkit/) to remove bases from the ends with a quality score less than 35. These high quality contigs were then *de novo* assembled via Trinity release 2012-04-27 (http://trinityrnaseq.sourceforge.net/) using “jellyfish” for k-mer counting. The *de novo* assembled transcripts from each developmental stage (ArrayExpress accession number E-MTAB-1184; http://www.ebi.ac.uk/arrayexpress/experiments/E-MTAB-1184) were tagged with the name of the developmental stage from which they were derived and merged into a single FASTA file. This FASTA file was then searched using the custom BLAST feature in Geneious version 5.5.6 (http://www.geneious.com/) [36] to search for *S. stercoralis* homologs of *C. elegans* genes.

### Identification and annotation of *S. stercoralis* genes

BLAST searches of the *S. stercoralis* (ftp://ftp.sanger.ac.uk/pub/pathogens/HGI/) and *S. ratti* (http://www.sanger.ac.uk/resources/downloads/helminths/strongyloides-ratti.html) genomic contigs using *C. elegans* protein sequences (http://www.wormbase.org/) were performed using Geneious set to the least restrictive parameters. Putative *S. stercoralis* homologs were identified through reverse BLAST searches using NCBI's pBLAST (http://blast.ncbi.nlm.nih.gov/Blast.cgi) [37] against *C. elegans* and/or phylum Nematoda sequences. Putative homologs were then manually annotated using aligned reads from all seven developmental stages by a combination of IGV and Geneious. Manually annotated *S. stercoralis* transcripts (Data S1, Data S2) were used to determine predicted protein sequences (Data S3).

Additional searches for ILP motifs in the *S. stercoralis* and *S. ratti* genomes were performed by translating the contigs in all six reading frames and searching for conserved A and B peptide motifs using Geneious. Similarly, we searched the *S. stercoralis de novo* assembled transcripts for ILP motifs by assembling the contigs from all developmental stages using Geneious, translating into all six reading frames, and searching for the B peptide motifs, C-11X-C and CPPG-11X-C, as well as the A peptide motifs, C-12X-CC, C-13X-CC, C-14X-CC, CC-3X-C-8X-CC, CC-4X-C-8X-CC, CC-3X-C-8X-C, and CC-3X-C-9X-C, where X represents any amino acid except for cysteine.

### Protein alignments and phylogenetic analysis

Protein alignments and phylogenetic analyses were performed when several *S. stercoralis* or *C. elegans* homologs with similar e-values were identified in an attempt to resolve the homology of the *S. stercoralis* genes. Predicted protein sequences for *S. stercoralis* genes were derived from manually annotated transcripts using Geneious. Protein alignments using related S. *stercoralis*, *C. elegans*, phylum Nematoda, and other kingdom Animalia protein sequences were generated with Clustal W, using a BLOSUM matrix, or MUSCLE and neighbor-joining phylogenetic trees constructed using Geneious. Accession numbers for protein alignments referred to in the text can be found in Data S4.

A protein alignment for full-length guanylyl cyclases, similar to *Ce*-DAF-11, was performed with Clustal W in Geneious (Data S5). A neighbor-joining tree with 100 iterations of boot-strapping was constructed using Geneious and inspected for clear homology between *Ce*-DAF-11 and nematode homologs (Figure S2).

A protein alignment for the TGFβ super-family ligands (Data S6) was performed using only the ligand domain, truncated at the first conserved cysteine residue [38], with Clustal W in Geneious. A neighbor-joining tree with 100 iterations of boot-strapping was constructed using Geneious. A protein alignment for the TGFβ ligand domains that included all cysteine residues was performed using MUSCLE in Geneious and manually corrected (Figure S3).

A protein alignment for the full-length SMADs (Data S7) using every publicly available phylum Nematoda sequence was performed with Clustal W in Geneious. A neighbor-joining tree with 100 iterations of boot-strapping was constructed using Geneious and inspected for clear homology between *C. elegans* proteins and other nematode homologs (Figure S4). Similarly, a protein alignment for full-length short-chain dehydrogenases related to *Ce*-DHS-16 (Data S8) was used to construct a neighbor-joining phylogenetic tree (Figure S5) to find an *S. stercoralis* homolog most similar to *Ce*-DHS-16. A similar approach was used for cytochrome P450 proteins related to *Ce*-DAF-9 to generate a protein alignment (Data S9) and construct a neighbor-joining phylogenetic tree (Figure S6) to find the *S. stercoralis* homolog most similar to *Ce*-DAF-9.

### Differential analysis of *S. stercoralis* transcripts

Transcript abundances of manually annotated *S. stercoralis* genes were calculated using Cufflinks version 2.0.0 (http://cufflinks.cbcb.umd.edu/) as fragments per kilobase of exon per million mapped reads (FPKM), with paired-end reads counted as single sampling events [39]. FPKM values for coding sequences (CDS) were calculated for each gene in each of the 21 samples and FPKM values for entire transcripts were calculated for each isoform in each of the 21 samples (Data S10). Log transformed values, ±95% confidence intervals, were plotted in Prism version 5.03 (GraphPad Software, Inc., http://www.graphpad.com/), and the y-axis was scaled from zero to 3.5 to aid comparisons between genes. Significant differences in FPKM values between developmental stages and p-values were determined using Cuffdiff version 1.3.0, a program with the Cufflinks package [40].

## Results

### RNAseq of seven *S. stercoralis* developmental stages

Many genes involved in *C. elegans* dauer regulation are transcriptionally regulated, including genes encoding ILPs [41], the dauer TGFβ ligand-encoding gene *Ce-daf-7* [42], and the genes encoding biosynthetic enzymes for DA [43] that regulate the NHR *Ce*-DAF-12 [44]. To acquire a comprehensive transcriptomic profile of the *S. stercoralis* homologs of these genes, as well as other genes potentially involved in *S. stercoralis* L3i developmental regulation, we undertook a next-generation RNA sequencing (RNAseq) approach using Illumina HiSeq technology.

Since *S. stercoralis* has a unique life cycle with a single free-living generation (Figure 1), several pair-wise comparisons can be made between life stages fated for free-living versus parasitic development. For RNAseq analysis, we examined the following developmental stages: gravid free-living females (FL Females), post-free-living first-stage larvae (PFL L1), infectious third-stage larvae (L3i), *in vivo* activated third-stage larvae (L3+), gravid parasitic females (P Females), predominantly (>95%) heterogonically developing post-parasitic first-stage larvae (PP L1), and post-parasitic larvae at approximately the third-stage developing heterogonically to free-living adults and enriched for females (PP L3).

**Figure 3:**
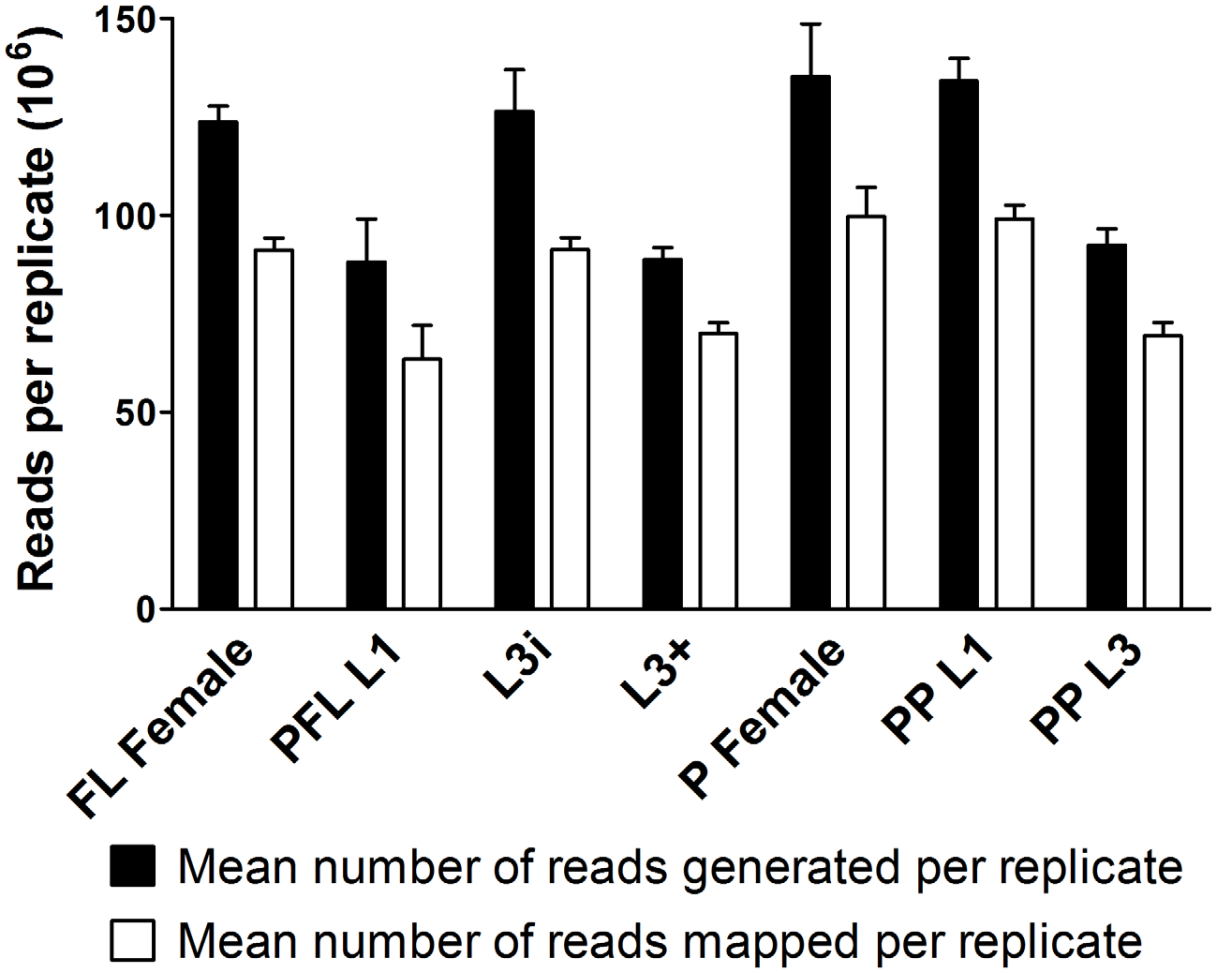
*S. stercoralis* RNAseq mean library sizes and number of reads aligning to the genome. A total of 21 libraries were derived from polyadenylated RNA and sequenced from seven developmental stages, each in biological triplicate. Paired-end 100 base-pair (bp) reads were generated from the following developmental stages: free-living females (FL Female), post-free-living first-stage larvae (PFL L1), infectious third-stage larvae (L3i), *in vivo* activated third-stage larvae (L3+), parasitic females (P Female), predominantly (>95%) heterogonically developing post-parasitic first-stage larvae (PP L1), and post-parasitic approximately third-stage larvae heterogonically developing to free-living adults and enriched for females (PP L3). The mean number of reads generated per replicate refers to the mean number of 100 bp reads sequenced (black bars) per biological replicate from each developmental stage. The mean number of mapped reads per replicate refers to the mean number of 100 bp reads aligned to *S. stercoralis* genomic contigs using TopHat (white bars) per biological replicate from each developmental stage. Error bars represent +1 standard deviation.

We isolated total RNA, in biological triplicate, from these seven developmental stages, using an *S. stercoralis* strain derived from a single free-living female (Data S11) [22] to decrease the number of nucleotide polymorphisms, which can confound alignment [30]. Using these samples, we constructed 21 polyadenylated RNA libraries, which we sequenced with 100 base-pair (bp) paired-end reads on an Illumina HiSeq 2000 instrument, generating a total of 2.36 billion reads (Figure 3). We independently aligned reads from each sample to the approximately 41 megabases of *S. stercoralis* genomic contigs using TopHat [40], [45], [46], a strategy used in the clade III parasitic nematode species *Ascaris suum* [31] and *Brugia malayi* [27]. Of the 2.36 billion reads initially sequenced, 1.75 billion (74%) aligned to genomic contigs (Figure 3). The roughly one quarter of reads that did not align to the genome may have come from contaminants such as gut bacteria or the gerbil host, contained sequencing errors, or originated from parts of the *S. stercoralis* genome that remain unsequenced.

### Identification of *S. stercoralis* genes encoding homologs of *C. elegans* dauer genes

To identify *S. stercoralis* homologs of the critical components involved in cGMP signaling, IIS, TGFβ signaling, as well as DA biosynthesis and NHR regulation, we performed BLAST searches of the *S. stercoralis* draft genome using *C. elegans* protein sequences. To confirm hits, we performed reverse BLAST searches to compare the manually annotated *S. stercoralis* sequences with *C. elegans* and phylum Nematoda databases [47]. When several homologs with similar e-values were present, we performed protein alignments and phylogenetic analysis to attempt to resolve the homology of *S. stercoralis* genes using related *S. stercoralis*, *C. elegans*, and phylum Nematoda protein sequences. For a few genes, we were unable to identify clear *C. elegans* homologs in *S. stercoralis* due to the lack of sequence similarity between the two species. We also noted several cases where either *S. stercoralis* or *C. elegans* had several closely related genes for which there was a single homolog in the other species, highlighting the evolutionary divergence between these two species, which are members of clade IV and clade V, respectively [16], [17].

We were unable to identify *S. stercoralis* homologs of several *C. elegans* genes within the *S. stercoralis* or closely related *S. ratti* genome sequences. To determine if these genes are absent from the genome assemblies, but present in the transcriptome, we performed *de novo* assembly of *S. stercoralis* transcripts with Trinity [48]. Using one sample from each developmental stage, we first merged each forward and reverse read pair to form a single, high quality “contig.” These merged single-read contigs were quality filtered and independently assembled to form expressed transcripts for each developmental stage. The seven expressed transcript libraries were merged to form a database on which we performed BLAST searches for *C. elegans* homologs not present in the draft *S. stercoralis* or *S. ratti* genomes. This *S. stercoralis* expressed transcript database contains a total of 210,709 developmental stage-specific transcripts; however, this includes redundant, fragmented, and un-spliced transcripts as well as contaminating sequences from gerbil and other environmental sources.

Due to the compactness of the *S. stercoralis* genome, we were unable to use Cufflinks [40], [49] to reliably predict transcripts because this program merged transcripts with untranslated region (UTR) overlap into single transcripts. Thus, we used aligned reads from all seven developmental stages to manually annotate exons and predict coding sequences for all isoforms of transcripts of interest. We then determined transcript abundances using Cufflinks to calculate fragments per kilobase of exon per million mapped reads (FPKM), with paired-end reads counted as single sampling events [39]. FPKM values were calculated for each gene or isoform in each developmental stage (Data S10), and significant differences between developmental stages were determined using the three biological replicates and Cuffdiff [40].

### Cyclic GMP signaling components are up-regulated in *S. stercoralis* L3i

In *C. elegans*, formation of dauer larvae is regulated by dauer pheromone [50], [51], a constitutively produced complex mixture of ascarosides [52], [53], which is indicative of population density. Dauer entry is promoted by dauer pheromone, which is sensed by several GTP-binding protein (G protein)-coupled receptors (GPCRs), including *Ce*-SRBC-64, *Ce*-SRBC-66, *Ce*-SRG-36, and *Ce*-SRG-37 [54], [55]. When bound by specific ascarosides, GPCRs activate G protein alpha subunits [55], including *Ce*-GPA-2 and *Ce*-GPA-3 [56], resulting in repression of the transmembrane guanylyl cyclase *Ce*-DAF-11 [57] and a decrease in cGMP levels. Intracellular cGMP levels regulate cyclic nucleotide-gated ion channels [58], composed of the *Ce*-TAX-4 α subunits [59] and *Ce*-TAX-2 β subunits, which result in neuron depolarization when activated. The *C. elegans* cGMP signaling pathway is epistatic to the TGFβ pathway [60] (Figure 2) and may regulate the production of the *Ce*-DAF-7 TGFβ ligand [61] as well as the IIS agonists *Ce*-DAF-28 and *Ce*-INS-7 [62], [63]. Other *daf* mutants have been identified that are critical both in the localization of these cGMP signaling pathway proteins to the cilia as well as in the formation of proper ciliary structures [64]. Developmental regulation of *C. elegans* cGMP signaling pathway genes during dauer arrest has not been well studied, although *Ce-gpa-2*, *Ce-gpa-3*, *Ce-daf-11*, *Ce-tax-2*, and *Ce-tax-4* are all down-regulated following dauer recovery in microarray analysis [43].

Outside of *C. elegans*, the role of ascarosides and cGMP pathway signaling in parasitic nematodes has been nearly overlooked. Muscarinic agonists and the cGMP analog 8-bromo-cGMP have been shown to activate *Ancylostoma caninum* L3i [65], [66], and we have previously cloned *S. stercoralis* homologs of *Ce-gpa-2* and *Ce-gpa-3* [18]. Recently, several groups have reported the presence of ascarosides in parasitic nematodes, which appear to differ in structure and composition between species and may play a role in L3i formation [67]–[69]. Thus, we sought to determine whether the components of a cGMP signaling pathway are present in *S. stercoralis* and whether these transcripts are developmentally regulated (Table 1).

**Table 1:** Comparison of cGMP signaling pathway homologs and transcript abundances in *S. stercoralis* and *C. elegans*.

*C. elegans* gene(s)	*S. stercoralis* homolog(s)	*S. stercoralis* transcript abundance profile	Regulation consistent with *C. elegans* (+, +/−, −)^1^
*Ce-gpa-2* & *-3*	*Ss-gpa-2* & *-3*	peak in L3i	+
*Ce-daf-11*	*Ss-gcy-11* 2	peak in L3i and L3+	+
*Ce-tax-2*	*Ss-tax-2*	peak in L3i	+
*Ce-tax-4*	*Ss-tax-4*	peak in L3i	+

1 (+) similar, (+/−) unclear, and (−) dissimilar transcript abundance patterns.

2 Homology is by phylogenetic similarity only.

**Figure 4:**
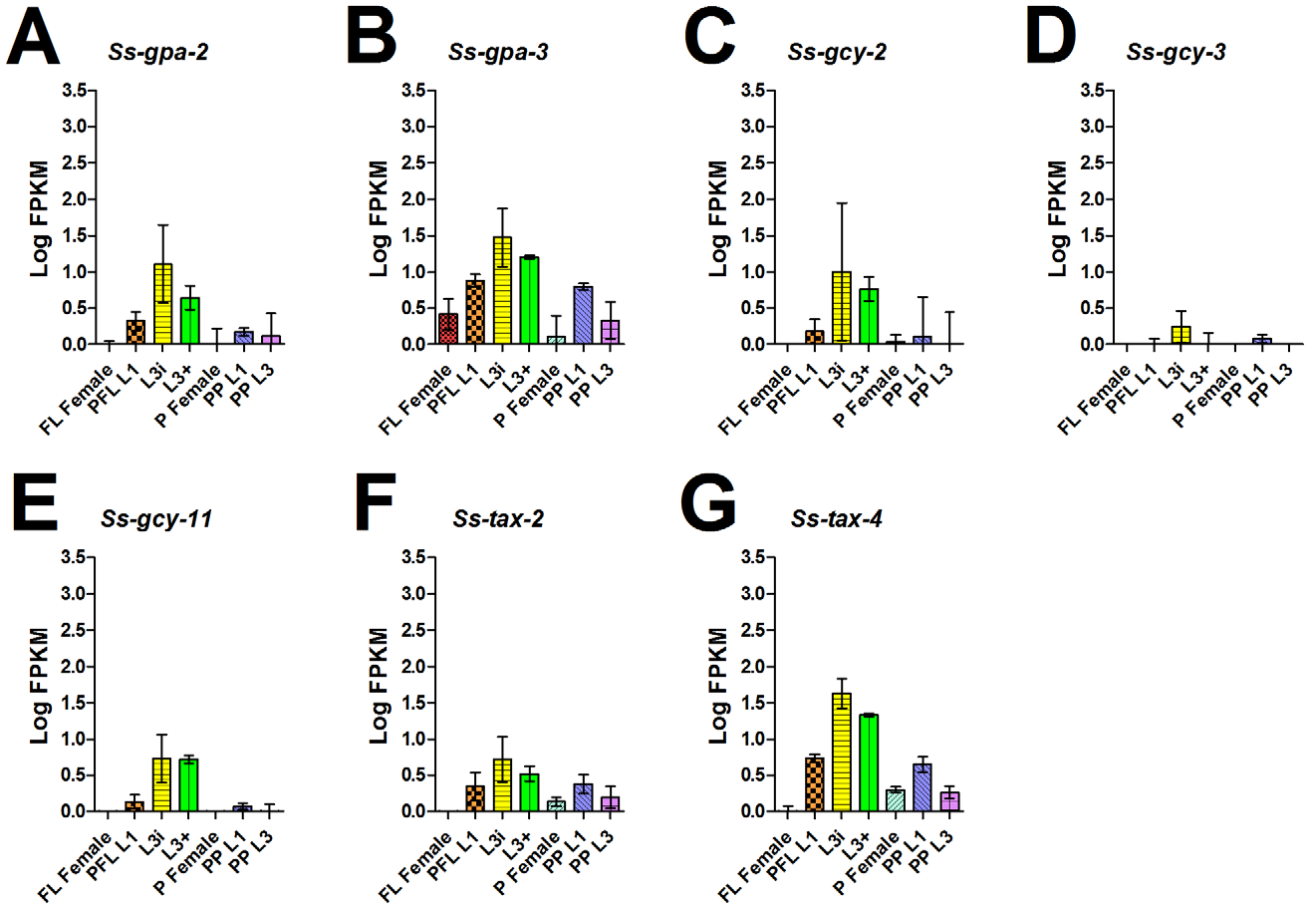
*S. stercoralis* cGMP signaling pathway homologs are coordinately up-regulated in L3i. Transcript abundances were determined for the coding region of: (A) *Ss-gpa-2* and (B) *Ss-gpa-3*, the genes encoding homologs of the G-protein α subunits *Ce*-GPA-2 and *Ce*-GPA-3, respectively; (C) *Ss-gcy-2*, (D) *Ss-gcy-3*, and (E) S*s-gcy-11*, genes which all encode guanylyl cyclase homologs, of which *Ss-gcy-11* encodes the *S. stercoralis* homolog most similar to the guanylyl cyclase *Ce*-DAF-11; (F) *Ss-tax-2* and (G) *Ss-tax-4*, genes encoding homologs of the cyclic nucleotide-gated ion channels *Ce*-TAX-2 and *Ce*-TAX-4, respectively. Transcript abundances were quantified in seven developmental stages: free-living females (FL Female), post-free-living first-stage larvae (PFL L1), infectious third-stage larvae (L3i), *in vivo* activated third-stage larvae (L3+), parasitic females (P Female), post-parasitic first-stage larvae (PP L1), and post-parasitic third-stage larvae (PP L3). Transcript abundances were calculated as fragments per kilobase of coding exon per million mapped reads (FPKM) and log transformed. Error bars represent 95% confidence intervals. The y-axes were scaled from 0 to 3.5 to aid comparison between genes.

We identified an *S. stercoralis* gene encoding a putative guanylyl cyclase that phylogenetically groups with *Ce*-DAF-11, which we termed *Ss-gyc-11* (Figure S2, Data S5). We also identified genes encoding homologs of the two cGMP-gated ion channels, *Ce*-TAX-2 and *Ce*-TAX-4, which we termed *Ss-tax-2* and *Ss-tax-4* respectively. We were unable to identify clear homologs of the GPCR genes, as many of the seven transmembrane receptor families have undergone rapid expansion in *C. elegans* [55]. Examination of the transcript abundance profiles for each of the five *S. stercoralis* genes putatively involved in cGMP pathway signaling revealed strikingly similar temporal regulation (Figure 4), with the steady-state level of each transcript at its peak in L3i and its nadir in both free-living and parasitic females. Interestingly, this developmental transcript abundance profile was also observed for two other guanylyl cyclases similar to *Ss-gyc-11* (Figure 4).

### Insulin-like peptide transcripts are regulated during *S. stercoralis* development

IIS plays a critical role in both dauer arrest and recovery in *C. elegans*. Both microarray [70] and careful transcript quantification experiments [41] have shown that regulation of *C. elegans* IIS transcripts during dauer development takes place at the level of the ILPs, while the intracellular signaling component transcripts are always present. We have previously shown that IIS in *S. stercoralis* plays a crucial role in L3i arrest [10] and activation [22]. However, neither the presence nor regulation of ILPs has been reported in *S. stercoralis* or any other parasitic nematodes.

**Figure 5:**
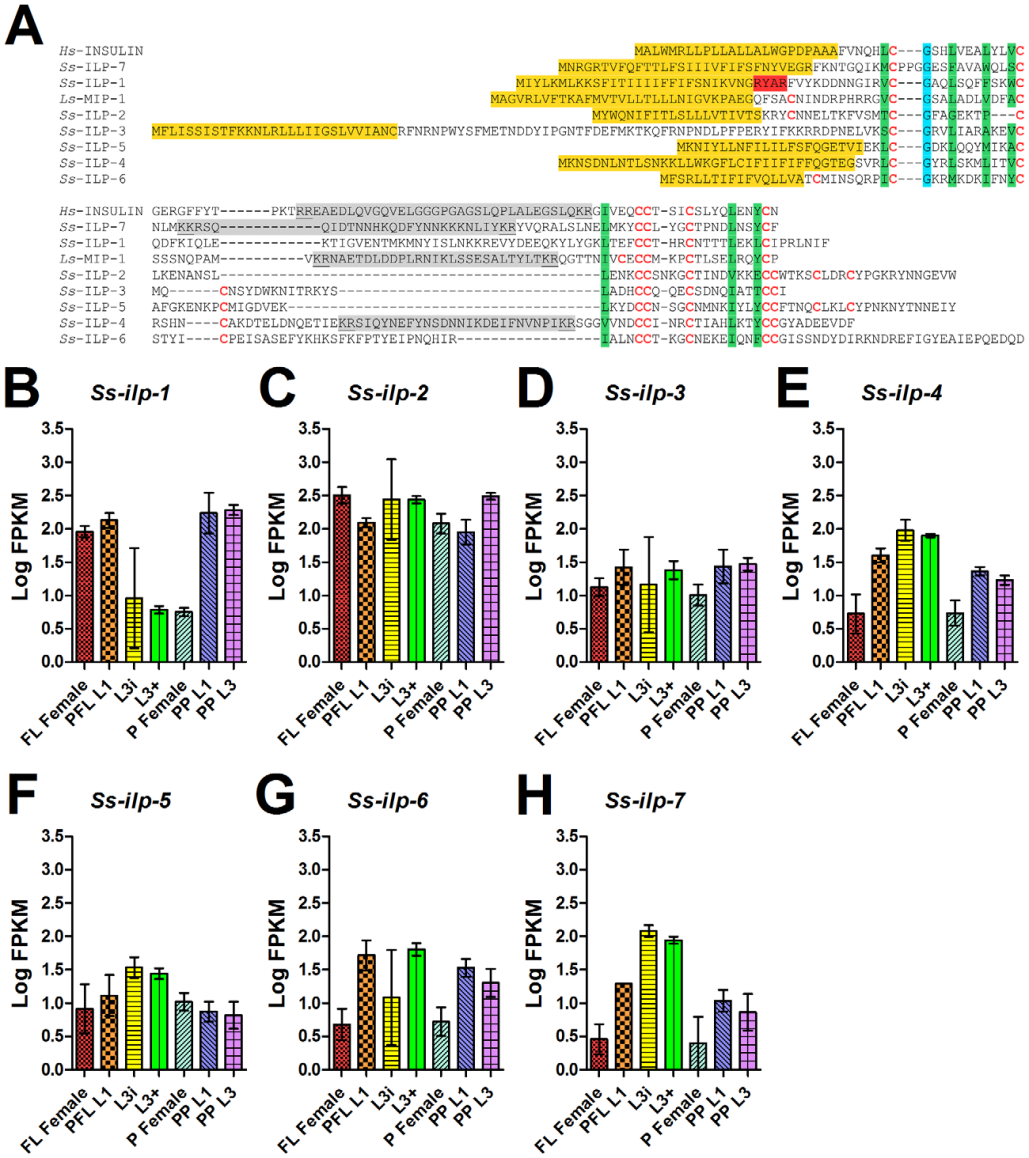
Protein sequence diversity and temporal regulation of *S. stercoralis* insulin-like peptides. (A) A predicted protein sequence alignment of seven *S. stercoralis* insulin-like peptides (ILPs), *Ss*-ILP-1 through -7, was constructed using human insulin (*Hs*-INSULIN) and *Lymnaea stagnalis* molluscan insulin-related peptide I (*Ls*-MIP-1) as the references. Cysteine residues, which are predicted to form disulfide bonds, are in red letters. Predicted signal sequences are highlighted in yellow, predicted furin recognition motifs are highlighted in red, hydrophobic residues important for helix formation are highlighted in green, and a conserved glycine is highlighted in blue. Predicted C peptides are highlighted in gray with dibasic predicted cleavage sites underlined. The B peptide is N-terminal of the C peptide, while the A peptide is C-terminal of the C peptide. (B-H) Transcript abundances were determined for the coding region of seven *S. stercoralis* ILP-encoding genes (*Ss-ilp-1* through *-7*) in seven developmental stages: free-living females (FL Female), post-free-living first-stage larvae (PFL L1), infectious third-stage larvae (L3i), *in vivo* activated third-stage larvae (L3+), parasitic females (P Female), post-parasitic first-stage larvae (PP L1), and post-parasitic third-stage larvae (PP L3). Transcript abundances were calculated as fragments per kilobase of coding exon per million mapped reads (FPKM) and log transformed. Error bars represent 95% confidence intervals. The y-axes were scaled from 0 to 3.5 to aid comparison between genes.

In *C. elegans*, 40 ILPs have been discovered and are thought to play redundant and complex roles in regulating dauer as well as other forms of development, with some ILPs agonizing and others antagonizing IIS [62], [71]. To find *S. stercoralis* ILPs, we performed BLAST searches of the draft genomes of *S. stercoralis* and *S. ratti* as well as our *de novo* assembled *S. stercoralis* transcripts using both *C. elegans* ILP protein sequences and conserved cysteine motifs in the A and B peptides [71]. In total, we identified seven *S. stercoralis* ILPs (Figure 5A, Table 2), which are also present in *S. ratti* (data not shown). The predicted protein sequences of the *S. stercoralis* ILPs are highly divergent from *C. elegans* homologs, except for several conserved cysteine residues which are predicted to form disulfide bonds. In contrast to both *C. elegans* and *Homo sapiens*, *S. stercoralis* ILPs lack the conserved intron located between N-terminal B peptide and C-terminal A peptide, and all but one lack a predicted furin cleavage site [71]–[73]. Furthermore, cleavable C peptides, located between the B and A peptides, are not conserved between species.

**Table 2:** Comparison of IIS pathway homologs and transcript abundances in *S. stercoralis* and *C. elegans*.

*C. elegans* gene(s)	*S. stercoralis* homolog(s)	*S. stercoralis* transcript abundance profile	Regulation consistent with *C. elegans* (+, +/−, −)^1^
**Insulin-like Peptides**			
Type α: *Ce-ins -20* to *-30*, and *-33* to *-36*	None identified		
Type β: *Ce-daf-28*, and *Ce-ins -1* to *-10*	*Ss-ilp-3*	present in all stages examined	+/−
	*Ss-ilp-4*	decreased in FL and P Females	+/−
	*Ss-ilp-6*	increased from L3i to L3+	+
Type γ: *Ce-ins-11* to *-19*, *-31*, *-32*, and *-37*	*Ss-ilp-1*	decreased in PFL generation	+
	*Ss-ilp-7*	increased in L3i and L3+	+
Type δ: None	*Ss-ilp-2* & *-5*	present in all stages examined	+/−
**Intracellular signaling components**			
*Ce-asna-1*	*Ss-asna-1*	present in all stages examined	+
*Ce-daf-2*	*Ss-daf-2*	increased in PFL generation	+
*Ce-ist-1*	*Ss-ist-1* & *-2*	present in all stages examined	+
*Ce-aap-1*	*Ss-aap-1*	increased in FL and P Females	+
*Ce-age-1*	*Ss-age-1*	increased in L3+	+
*Ce-daf-18*	*Ss-pten-1* & -*2*	present in all stages examined	+
*Ce-pdk-1*	*Ss-pdk-1*	increased in L3i	+
*Ce-sgk-1*	*Ss-sgk-1*	absent in L3i and L3+	-
*Ce-akt-1* and *-2*	*Ss-akt-1*	increased in L3i and L3+	+/−
*Ce-pptr-1* 2	*Ss-pptr-1*	present in all stages examined	+
*Ce-ftt-2*	*Ss-ftt-2*	present in all stages examined	+
*Ce-daf-16*	*Ss-daf-16*	decreased in FL and P Females	+/−
***Ce*** **-DAF-16 regulated genes**			
*Ce-sod-3* 3	*Ss-sod-1*	present in all stages examined	−
*Ce-daf-15*	*Ss-daf-15*	present in all stages examined	−
*Ce-acs-19*	*Ss-acs-19*	present in all stages examined	−
*Ce-ldb-1*	*Ss-limdb-1* & *-2*	present in all stages examined	−
*Ce-pitp-1*	*Ss-pitp-1*	present in all stages examined	−
*Ce-Y105E8B.9*	*Ss- Y105E8B.9*	increased in developing larvae	+/−

1 (+) similar, (+/−) unclear, and (−) dissimilar transcript abundance patterns.

2 A homolog for the closely related gene *Ce-pptr-2* was identified and termed *Ss-pptr-2*.

3 The closely related gene *Ce-sod-2* was accounted for; only one *sod* gene was identified in *S. stercoralis*.

The *S. stercoralis* putative ILPs—*Ss*-ILP-3, *Ss*-ILP-4, and *Ss*-ILP-6—have type β cysteine architecture [71]. In *C. elegans*, the type β family includes several agonistic ligands including *Ce*-DAF-28 [62], *Ce*-INS-6 [62], [74], [75], and *Ce*-INS-7 [76], as well as the antagonistic ligand *Ce*-INS-1 [71], [74], [77]–[79]. The type β family also includes the *Lymnaea stagnalis* molluscan insulin-related peptide I (MIP-1) [80]. In contrast, *Ss*-ILP-1 and *Ss*-ILP-7 have type γ cysteine architecture, similar to that found in human insulin [71]. In *C. elegans*, the type γ family includes the putative antagonist *Ce*-INS-18, which has a PPG motif between the conserved cysteine and glycine residues in the B peptide [71], [81]. Interestingly, *Ss*-ILP-7 is the only *S. stercoralis* ILP to share this motif (Figure 5A). Unlike the six cysteine residues found in type α and γ ILPs or the eight found in type β ILPs, *Ss*-ILP-2 and *Ss*-ILP-5 have 10 cysteine residues. We propose that *Ss*-ILP-2 and *Ss*-ILP-5 represent a novel class of nematode ILPs, which we term type δ.

To determine whether *S. stercoralis* ILP transcripts are developmentally regulated, we compared FPKM values for each transcript between developmental stages (Figure 5B–H). In contrast to many *C. elegans* ILPs which are only expressed at one or a few developmental stages [41], transcripts encoding all seven *S. stercoralis* ILPs were detected in all developmental stages examined. We noted that *Ss-ilp-1* transcripts are decreased in L3i and significantly down-regulated in L3+ and parasitic females compared to the other developmental stages examined (p<0.001). We also noted that transcripts for both *Ss-ilp-4* and *Ss-ilp-7*, encoding the only two *S. stercoralis* ILPs with predicted C peptides that are cleaved, are at their peak in L3i. Additionally, we observed high variability in the transcript abundances of several ILP-encoding genes in the L3i developmental stage, evidenced by the large 95% confidence intervals. Since we isolated L3i incubated at 21°C after 8 and 10 days of culture or 25°C after 7 of days of culture (Data S11), we plotted transcript abundance for each *ilp* gene by relative age for each biological replicate (Figure S7). This analysis revealed that the error was not stochastic, but rather a developmental trend dependent upon the relative age of the L3i. In this analysis, we observed a one log increase in the transcript abundance of *Ss-ilp-6* from the oldest L3i to the L3+.

### Intracellular IIS component transcripts are always present in *S. stercoralis*

While *C. elegans* ILPs are developmentally regulated, intracellular IIS components are always present [41], [70]. We have previously cloned and detected transcripts throughout the life cycle of *S. stercoralis* homologs of both the forkhead transcription factor *daf-16* [82] and the *age-1* catalytic subunit of the phosphatidylinositol-3 kinase (PI3K) [22]. Recently, we have also cloned and characterized the *S. stercoralis* genes encoding the *Ss*-AAP-1 PI3K accessory/regulatory subunit [22] and the *Ss*-DAF-2 insulin-like receptor (Massey, HC, *et al.*, in preparation). In this study, we asked whether homologs of the remaining IIS components are present in *S. stercoralis* and, if so, whether their transcripts are also present throughout the life cycle (Table 2).

Downstream of the DAF-2 IIS receptor, we identified two genes encoding homologs of the insulin receptor substrate *Ce*-IST-1 [83], which we termed *Ss-ist-1* and *Ss-ist-2*. Interestingly, we also found two homologs of the gene encoding the *C. elegans* phosphatase and tensin (PTEN) homolog *Ce*-DAF-18, which opposes the function of the PI3K *Ce*-AGE-1 when IIS is activated [84]. We termed these genes *Ss-pten-1* and *Ss-pten-2*. We also identified *Ss-pdk-1* as a homolog of the gene encoding the 3-phosphoinositide-dependent kinase *Ce*-PDK-1, which phosphorylates and activates *Ce*-AKT-1 and -2 when IIS is activated [85]. We identified *Ss-akt-1* as a single homolog of the genes encoding the *C. elegans* serine/threonine kinases *Ce*-AKT-1 and *Ce*-AKT-2 [86], which phosphorylate *Ce*-DAF-16 when IIS is activated [87], [88]. In *C. elegans*, AKT-1 is negatively regulated by *Ce*-PPTR-1, a B56 regulatory subunit of the PP2A phosphatase [89]. We identified an *S. stercoralis* gene encoding a similar phosphatase, which we termed *Ss-pptr-1*. We also found a gene encoding the *C. elegans* homolog of the serum- and glucocorticoid-inducible kinase *Ce*-SGK-1 that regulates *Ce*-DAF-16 [90], which we termed *Ss-sgk-1*. We identified a homolog of the gene encoding the 14-3-3 protein *Ce*-FTT-2 [91] that regulates *Ce*-DAF-16 [92], which we termed *Ss-ftt-2*. Additionally, we found *Ss-asna-1*, a homolog of the gene encoding the ATPase *Ce*-ASNA-1, which regulates ILP secretion in *C. elegans* [93]. Together, these *S. stercoralis* homologs reconstruct a complete IIS pathway similar to that found in *C. elegans* and other metazoans [94].

Transcripts for each of the *S. stercoralis* genes encoding IIS cytoplasmic signaling proteins, except for *Ss-sgk-1*, were detected in every developmental stage examined (Figure S8), suggesting that the IIS cytoplasmic signaling proteins are present throughout the *S. stercoralis* life cycle. We observed varying degrees of transcript up-regulation in the post-free-living generation of genes encoding the core IIS cytoplasmic signaling proteins *Ss*-DAF-2, *Ss*-AGE-1, *Ss*-PDK-1, *Ss*-AKT-1, and *Ss*-DAF-16. Interestingly, the increases in *Ss-akt-1* transcripts in the L3i and L3+ stages were largely due to expression of a second isoform, *Ss-akt-1b*, which encodes a predicted peptide with a shortened N-terminus that results in a 33 amino acid deletion from the AKT pleckstrin homology (PH) domain and which is only present in these two stages (Figure S9). Conversely, we noted an absence of *Ss-sgk-1* transcripts in L3i and L3+ (Figure S8).

### Homologs of *Ce*-DAF-16-regulated genes are not similarly regulated in *S. stercoralis* development

To determine whether IIS regulates similar genes in *S. stercoralis* and *C. elegans*, we then asked whether homologs of genes transcriptionally regulated by *Ce*-DAF-16 were similarly regulated over the course of *S. stercoralis* development (Table 2). In *C. elegans*, multiple studies have examined the genes regulated by the transcription factor *Ce*-DAF-16 [76], [95]–[98]. The superoxide dismutase encoding gene *Ce-sod-3* is a well-characterized gene that is up-regulated by *Ce*-DAF-16 in the dauer stage [41], [99], [100], while the RAPTOR ortholog-encoding gene *Ce-daf-15* is down-regulated by *Ce*-DAF-16 in low IIS conditions [101]. We identified a single superoxide dismutase-encoding gene in *S. stercoralis* that phylogenetically grouped with *Ce-sod-2* and *Ce-sod-3*, which we termed *Ss-sod-1*, as well as a homolog of *Ce-daf-15*, which we termed *Ss-daf-15*. Additionally, we identified *S. stercoralis* homologs of *Ce-acs-19*, *Ce-ldb-1*, *Ce-pitp-1*, and *Ce-Y105E8B.9*, all of which were identified as *Ce*-DAF-16 targets by ChIPseq, are differentially regulated in *Ce-daf-16(mu86)* mutants, and have a phenotype associated with loss of *Ce*-DAF-16 function upon RNAi knock-down [98]. We termed these homologs *Ss-acs-19*, *Ss-limdb-1* and *-2*, *Ss-pitp-1*, and *Ss-Y105E8B.9*, respectively.

Surprisingly, transcript abundance profiles for each of these six genes (Figure S10) revealed that neither *Ss-sod-1*, *Ss-daf-15*, nor the other five genes were up- or down-regulated in L3i. In fact, no large differences in *Ss-sod-1* or *Ss-daf-15* transcript levels were observed among any of the seven developmental stages examined.

### The DAF-7-like TGFβ ligand family is expanded in *S. stercoralis*

In *C. elegans*, mutation of the TGFβ ligand-encoding gene *daf-7* results in temperature sensitive dauer arrest and is the only TGFβ ligand in the *C. elegans* genome in the same family as human TGFβ1, Inhibin/Activin, and Myostatin [42], [102]. *Ce-daf-7* transcripts are at their peak in L1 larvae and are up-regulated during recovery from both L1 and dauer arrested states [23], [42], [43]. In *C. elegans*, DAF-7 is most likely produced in response to food cues and functions in parallel with other pathways to promote continuous development.

Previous work in *S. stercoralis*, *S. ratti*, and *Parastrongyloides trichosuri* has identified *Ce*-DAF-7-like TGFβ ligand-encoding genes, named *Ss*-*tgh-1*, *Sr-daf-7*, and *Pt-daf-7*, respectively [20], [23]. In stark contrast to *C. elegans*, these clade IV parasitic nematode TGFβ ligands are significantly up-regulated in the developmentally arrested L3i and down-regulated in activated L3i—a pattern directly opposite to that predicted under the dauer hypothesis. Similarly, transcripts encoding a DAF-7-like TGFβ ligand, termed *tgh-2*, have been described in the clade V parasitic nematodes *Ancylostoma caninum* [103], [104], *Heligmosomoides polygyrus*, *Nippostrongylus brasiliensis*, *Haemonchus contortus*, and *Teladorsagia circumcincta* [105], as well as the clade III parasitic nematodes *Brugia malayi* and *Brugia pahangi* [106]. For many of these nematode species, the *tgh-2* transcripts are up-regulated in the L3i. These observations have led some groups to question the relevance of using *C. elegans* dauer pathways to predict pathways regulating infectious larval development in parasitic nematodes [107].

**Figure 6:**
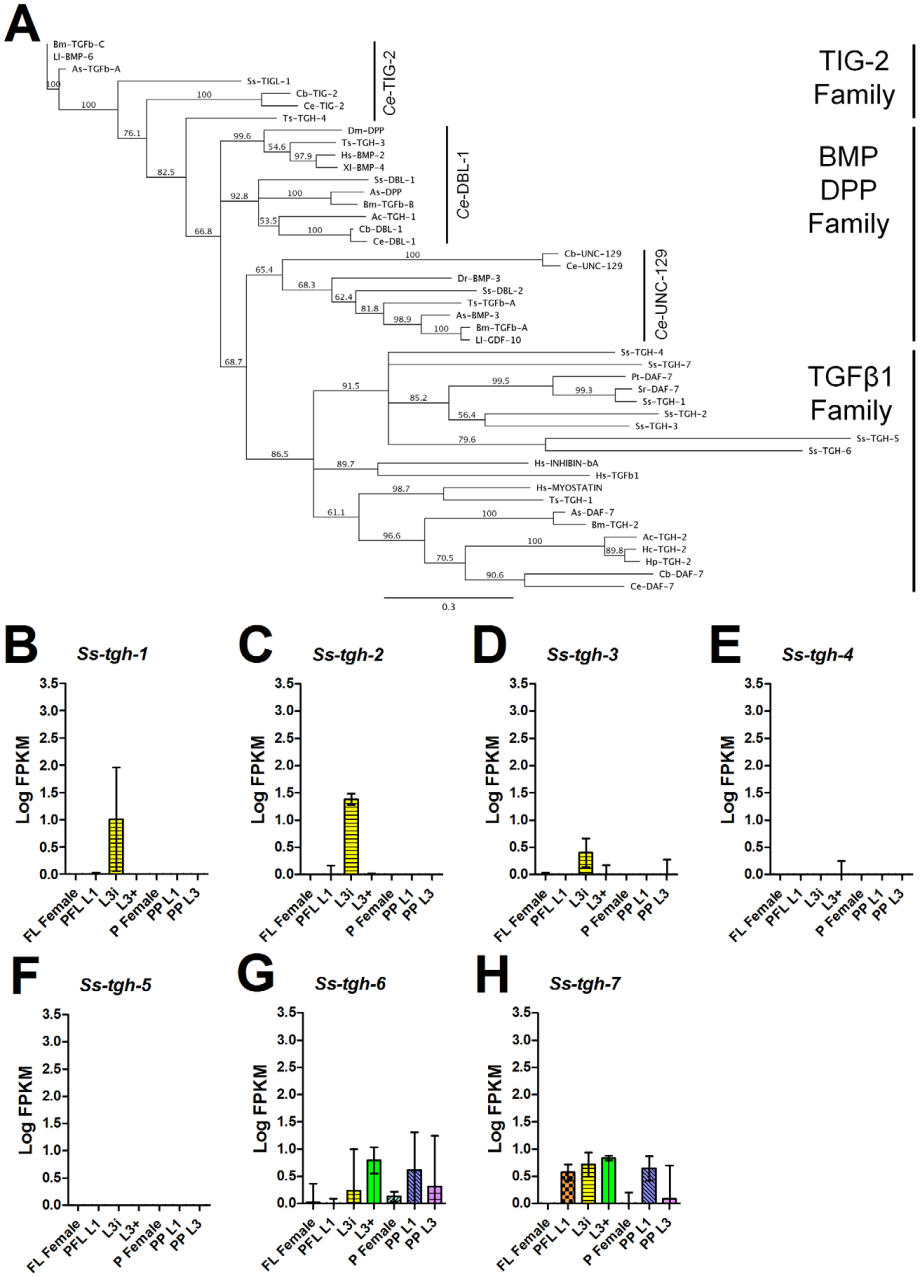
Phylogenetic analysis and temporal regulation of *S. stercoralis* TGFβ ligands. (A) Phylogenetic analysis of the transforming growth factor β (TGFβ) super-family ligands was performed; nematode TGFβ ligands resolved into three main families that share the same cysteine architecture. *Ss*-TIGL-1 groups with the *Ce*-TIG-2-like family; *Ss*-DBL-1 and *Ss*-DBL-2 group with the *D. melanogaster* decapentaplegic (DPP) and vertebrate bone morphogenetic protein (BMP) family; and *Ss*-TGH-1 through -7 group with the human TGFβ1 family that also includes *Ce*-DAF-7. A Clustal W alignment of the TGFβ ligands truncated at the first conserved cysteine was used to construct the neighbor-joining tree with 100 iterations of boot-strapping. Abbreviations: *Ancylostoma caninum* (Ac), *Ascaris suum* (As), *Brugia malayi* (Bm), *Caenorhabditis briggsae* (Cb), *Caenorhabditis elegans* (Ce), *Danio rerio* (Dr), *Drosophila melanogaster* (Dm), *Haemonchus contortus* (Hc), *Heligmosomoides polygyrus* (Hp), *Homo sapiens* (Hs), *Loa loa* (Ll), *Parastrongyloides trichosuri* (Pt), *Strongyloides ratti* (Sr), *Strongyloides stercoralis* (Ss), *Trichinella spiralis* (Ts), and *Xenopus laevis* (Xl). The scale bar represents substitutions per position. Accession numbers are listed in Data S4. (B–H) Transcript abundances were determined for the coding region of seven *S. stercoralis* genes, *Ss-tgh-1* through *-7*, encoding putative TGFβ ligands similar to *Ce*-DAF-7 in seven developmental stages: free-living females (FL Female), post-free-living first-stage larvae (PFL L1), infectious third-stage larvae (L3i), *in vivo* activated third-stage larvae (L3+), parasitic females (P Female), post-parasitic first-stage larvae (PP L1), and post-parasitic third-stage larvae (PP L3). Transcript abundances were calculated as fragments per kilobase of coding exon per million mapped reads (FPKM) and log transformed. Error bars represent 95% confidence intervals. The y-axes were scaled from 0 to 3.5 to aid comparison between genes.

In addition to *Ce*-DAF-7, *C. elegans* also has four other TGFβ ligands that have different cysteine architecture and are not involved in dauer regulation; thus, we sought to identify homologs of all the TGFβ ligands in *S. stercoralis* to ensure proper classification. To our surprise, we discovered a total of 10 TGFβ ligands in both the *S. stercoralis* draft genome (Figure 6A) and *S. ratti* draft genome (data not shown). Protein alignment and phylogenetic analysis placed seven of these ligands in the same family as *Ce*-DAF-7, which also includes the previously described *Ss*-TGH-1 (Figure 6A, Figure S3, Data S6). We named these additional *Ss-tgh-1*-like genes *Ss-tgh-2* through *-7* (Table 3). Interestingly, the putative *Ss*-TGH-6 and *Ss*-TGH-7 ligands are not predicted to have propeptides, an observation previously reported in TGH-2 from *N. brasiliensis* [105], *Schistosoma mansoni* SmInAct [108], and a few TGFβ ligands from Ctenophores (marine invertebrates commonly called comb jellies) [38]. The three additional *S. stercoralis* TGFβ ligands grouped with homologs of *Ce*-DBL-1, *Ce*-UNC-129, and *Ce-*TIG-2 [109] by both phylogenetic analysis (Figure 6A) and protein alignment (Figure S3). We termed the genes encoding these ligands *Ss-dbl-1*, *Ss-dbl-2*, and *Ss-tigl-1*, respectively.

**Table 3:** Comparison of dauer TGFβ signaling pathway homologs and transcript abundances in *S. stercoralis* and *C. elegans*.

*C. elegans* gene(s)	*S. stercoralis* homolog(s)	*S. stercoralis* transcript abundance profile	Regulation consistent with *C. elegans* (+, +/−, −)^1^
*Ce-daf-7* 2	*Ss-tgh-1* to *-3*	L3i only	−
	*Ss-tgh-4* & *-5*	not present in stages examined	+/−
	*Ss-tgh-6*	increased from PFL L1 to L3+	−
	*Ss-tgh-7*	not present in FL or P Females	+/−
*Ce-daf-1*	*Ss-daf-1*	increased in L3i and L3+	−
*Ce-daf-4* 3	*Ss-daf-4*	increased in L3i	−
*Ce-bra-1*	*Ss-bra-1*	present in all stages examined	+/−
*Ce-daf-3*, *-8*, & *-14* 4	*Ss-smad-1*	present in all stages examined	+/−
	*Ss-smad-5*	decreased in P Females	+/−
	*Ss-smad-7*	present in all stages examined	+/−
	*Ss-smad-8*	decreased in FL and P Females	+/−
*Ce-pdp-1*	*Ss-pdp-1*	present in all stages examined	+/−
*Ce-daf-5* 5	*Ss-daf-5*	present in all stages examined	+/−

1 (+) similar, (+/−) unclear, and (−) dissimilar transcript abundance patterns.

2 Homologs for the *Ce-dbl-1*, *Ce-unc-129*, and *Ce-tig-2* were identified and termed *Ss-dbl-1*, *Ss-dbl-2*, and *Ss-tigl-1*, respectively.

3 A homolog for the related gene *Ce-sma-6* was identified and termed *Ss-sma-6*.

4 Homologs for *Ce-sma-2*, *Ce-sma-3*, and *Ce-sma-4* were identified and termed *Ss-smad-2*, *Ss-smad-3*, and *Ss-smad-4*, respectively.

5 A homolog for *Ce-sma-9* was identified and termed *Ss-sma-9*.

We investigated whether the transcript abundance patterns of the seven genes encoding *S. stercoralis* TGH ligands were similar to *Ss-tgh-1* (Figure 6B–H). Interestingly, *Ss-tgh-1*, *-2*, and *-3* transcripts were detected exclusively in L3i, while *Ss-tgh-4* and *-5* were not detected in any of the life stages examined. *Ss-tgh-6* and *-7* had more complex transcript abundance patterns; *Ss-tgh-6* was up-regulated in L3+ in comparison to L3i (p<0.001), while *Ss-tgh-7* was not expressed in either the free-living or parasitic females. Similar to the ILP-encoding genes, the *tgh* genes also had a high degree of variability in the transcript abundances in the L3i developmental stage. As with the *ilp* genes, the variability of the *tgh* genes in L3i represented developmental trends that are dependent upon the relative age of the L3i (Figure S7). We also determined transcript abundances for *Ss-dbl-1*, *Ss-dbl-2*, and *Ss-tigl-1*, which are not predicted to signal through the dauer TGFβ signaling pathway (Figure S11).

### Dauer TGFβ signaling pathway components are present in *S. stercoralis*, but have high sequence divergence

Components of the *C. elegans* dauer TGFβ signaling pathway all have a temperature sensitive dauer phenotype when mutated [60]. Recent studies have presented an integrated model for dauer TGFβ signaling [110], [111], where under well-fed conditions, the *Ce*-DAF-7 ligand is expressed [42], [112] and binds the type I receptor *Ce*-DAF-1 [113] and type II receptor *Ce*-DAF-4 [114], overcoming the inhibition of *Ce*-DAF-1 by *Ce*-BRA-1 [115]. This results in phosphorylation and activation of the cytoplasmic R-SMADs *Ce*-DAF-8 [110] and *Ce*-DAF-14 [116], which together repress the Co-SMAD *Ce*-DAF-3 [117] and allow for reproductive development. However, when the *Ce*-DAF-7 ligand is not present, *Ce*-DAF-3 is active [110] and, together with the Sno/Ski-like transcriptional co-factor *Ce*-DAF-5 [118], represses expression of *Ce-daf-7* and *Ce-daf-8* [110], thereby promoting dauer development (Figure 2). In *C. elegans*, *Ce*-DAF-8 and *Ce*-DAF-14 are also inhibited by the phosphatase *Ce*-PDP-1, which also appears to control components of IIS, including ILPs, suggesting cross-talk between these pathways [111].

Proteins of the *C. elegans* dauer TGFβ pathway have diverged from those of other metazoans in both structure and function. *Ce*-DAF-1 can signal to some extent without *Ce*-DAF-4 [119], and a truncated *Ce*-DAF-4 protein expressed in dauers can negatively regulate *Ce*-DAF-7 signaling [120]. Consensus SMADs have both an MH1 (DNA-binding) and an MH2 (protein-protein interacting) domain and are activated by TGFβ signaling [121]; however, *Ce*-DAF-14 does not contain a consensus MH1 domain [116] and *Ce*-DAF-3 is repressed by *Ce*-DAF-7 signaling [117]. Temporal regulation of multiple components has been observed, including an up-regulation of *Ce*-DAF-1 [119] and *Ce*-DAF-8 [110] in L1 similar to *Ce-daf-7* transcriptional regulation [42], as well as a decrease in full-length *Ce-daf-4* transcripts in dauer larvae [120].

Since we observed a marked increase in the number of *Ce*-DAF-7-like TGFβ ligands in *S. stercoralis*, we asked whether the dauer TGFβ cytoplasmic signaling components were conserved in both protein structure and temporal regulation (Table 3). We sought to differentiate these components from those in the *C. elegans* small body size and male tail abnormal (Sma/Mab) TGFβ pathway. We identified homologs of the genes encoding the *Ce*-DAF-1 type I receptor and the *Ce*-DAF-4 type II receptor, which we termed *Ss-daf-1* and *Ss-daf-4*, respectively. We also identified a homolog of the gene encoding the *Ce*-DAF-1 negative regulator *Ce*-BRA-1, which we termed *Ss-bra-1*. The *C. elegans* Sma/Mab TGFβ pathway, which uses the *Ce*-DBL-1 ligand [122], [123], also utilizes the *Ce*-DAF-4 type II receptor but with *Ce*-SMA-6 as the type I receptor [124]. To ensure proper classification of the type I receptors, we identified a gene encoding a homolog of *Ce*-SMA-6, which we termed *Ss-sma-6*.

Identification of homologs for each of the SMADs proved difficult and was confounded by structurally similar SMADs involved in the dauer and Sma/Mab TGFβ signaling pathways present in *C. elegans* [102]. We identified a gene encoding a homolog of *Ce*-DAF-14 that did not include a MH1 domain, which we termed *Ss-smad-1*. We identified three *S. stercoralis* genes, termed *Ss-smad-5*, *Ss-smad-7*, and *Ss-smad-8*, which encode SMADs similar to *Ce*-DAF-3 and *Ce*-DAF-8; however, we were unable to resolve homology further by protein alignment or phylogenetic analysis (Figure S4, Data S7). Interestingly, we were able to clearly resolve genes encoding Sma/Mab TGFβ pathway SMADs similar to *Ce*-SMA-2, *Ce*-SMA-3, and *Ce*-SMA-4, which we termed *Ss-smad-2*, *Ss-smad-3*, and *Ss-smad-4*, respectively.

We identified a gene encoding a dauer TGFβ pathway *Ce*-DAF-5-like transcriptional co-factor, which we termed *Ss-daf-5*. The gene encoding a homolog of the Sma/Mab TGFβ pathway *Ce*-SMA-9-like transcriptional co-factor, which we termed *Ss-sma-9*, was clearly differentiable from *Ss-daf-5*. We also identified a gene encoding a phosphatase similar to *Ce*-PDP-1, which we termed *Ss-pdp-1*.

Examination of the transcript abundance patterns of the *S. stercoralis* genes encoding dauer pathway TGFβ homologs revealed several interesting trends (Figure S12). In direct contrast to the down-regulation of the type I and type II receptors observed in *C. elegans* dauer larvae [119], [120], *Ss-daf-1* and *Ss-daf-4* transcripts are at their peak in L3i and L3+. Likewise, *Ss-smad-8* transcripts were also at their peak in L3i. These observations are consistent with the expression of the *Ss-tgh-1*, *Ss-tgh-2*, and *Ss-tgh-3* transcripts exclusively in L3i (Figure 6B–D). We also noted a significant decrease in *Ss-smad-5* transcripts in parasitic females in comparison to the other six developmental stages examined (p<0.001). We did not observe any changes greater than one log in the transcript abundance of *Ss-bra-1*, *Ss-smad-1*, *Ss-smad-7*, or *Ss-daf-5* in the seven developmental stages examined. Additionally, we examined the transcript abundances of the components in the Sma/Mab TGFβ pathway and noted that transcript levels for the receptor-encoding genes, *Ss-sma-6* and *Ss-daf-4*, as well as the *Ss-sma-9* transcriptional co-factor, are at their peak in L3i (Figure S12).

### A putative dafachronic acid biosynthetic pathway is present in *S. stercoralis*

In *C. elegans* dauer development, epistatic analysis has placed both the IIS and dauer TGFβ pathways upstream of the NHR *Ce*-DAF-12 [125] (Figure 2). *Ce*-DAF-12 is broadly expressed [126] and is regulated by at least two steroid-like ligands, known as Δ^4^- and Δ^7^-dafachronic acid (DA) [44]. These DAs are synthesized from cholesterol, which is trafficked intracellularly by *Ce*-NCR-1 and -2 [127]. For Δ^7^-DA synthesis, cholesterol is first modified by the Rieske-like oxygenase *Ce*-DAF-36 [128], followed by the short-chain dehydrogenase *Ce*-DHS-16 [129]. In the final step, the cholesterol side chain is oxidized by the cytochrome P450 *Ce*-DAF-9 [130], [131], with likely assistance from the cytochrome P450 reductase *Ce*-EMB-8 [129]. The enzymes that synthesize the precursors of Δ^4^-DA are unknown, although the final oxidation step(s) are carried out by *Ce*-DAF-9 and *Ce*-EMB-8, similarly to Δ^7^-DA [129]. The 3β-hydroxysteriod dehydrogenase/Δ^5^-Δ^4^ isomerase *Ce*-HSD-1 has previously been reported to play a role in Δ^4^-DA biosynthesis [132]; however, a recent study has shown that this is not the case and that *Ce*-HSD-1 may be involved in synthesizing other DAs [129]. Additionally, the *Ce*-STRM-1 methyltransferase modifies DA precursors and can influence dauer development [133].

In favorable environmental conditions and when dauer larvae resume development, DAs are synthesized and bind *Ce*-DAF-12 [44] to promote reproductive development. However, in unfavorable environmental conditions, DAs are not synthesized and *Ce*-DAF-12, along with its co-repressor *Ce*-DIN-1 [134], promotes dauer development. Expression of GFP reporter constructs from *Ce-daf-36* [128] and *Ce-daf-12* [126] promoters is down-regulated in dauers, while microarray evidence has shown that *Ce-daf-9* and *Ce-daf-36* transcripts are up-regulated during dauer recovery [43]. Somewhat contradictorily, *Ce-daf-12* transcripts have been shown to be up-regulated during dauer formation [135].

The *S. stercoralis* homolog of DAF-12 has been cloned [21], and recent evidence from our lab has demonstrated that exogenous application of Δ^7^-DA to *S. stercoralis* L3i results in potent activation, as measured by resumption of feeding, in the absence of all host-like cues [136]. Furthermore, Δ^7^-DA applied to *S. stercoralis* post-free-living larvae results in failure to arrest as L3i and development to free-living L4, which we have termed an “L3i bypass” phenotype [136]. In the closely related parasite *Strongyloides papillosus*, which has a life cycle outside the host very similar to that of *S. stercoralis*, application of Δ^7^-DA to post-free-living larvae results in a second free-living generation of reproductively competent females [137]. In both *S. stercoralis* and *S. papillosus*, Δ^7^-DA results in stronger L3i activation or L3i bypass phenotypes than does Δ^4^-DA [136], [137].

Therefore, we asked whether a biosynthetic pathway for NHR DA ligand(s) similar to that found in *C. elegans* was present in *S. stercoralis* and had similar developmental regulation (Table 4). We identified a single *S. stercoralis* gene encoding a homolog of *Ce*-NCR-1 and -2, which we termed *Ss-ncr-1*, as well as a gene encoding a homolog of *Ce*-DAF-36, which we termed *Ss-daf-36*. We identified several *S. stercoralis* genes encoding putative short-chain dehydrogenases similar to *Ce*-DHS-16; one of these genes, which we termed *Ss-scdh-16*, encoded a predicted protein that phylogenetically grouped closely with *Ce*-DHS-16 (Figure S5, Data S8). Similarly, we identified several *S. stercoralis* genes putatively encoding cytochrome P450s similar to *Ce*-DAF-9; one of these, which we termed *Ss-cyp-9*, encoded a putative peptide that grouped with *Ce*-DAF-9 by phylogenetic analysis (Figure S6, Data S9). We also identified a gene encoding a homolog of *Ce*-EMB-8, which we termed *Ss-emb-8*, as well as a gene encoding a homolog of *Ce*-STRM-1, which we termed *Ss-strm-1*. Curiously, we were unable to identify genes encoding *S. stercoralis* homologs of *Ce*-HSD-1 or *Ce*-DIN-1 in the *S. stercoralis* draft genome, the *S. ratti* draft genome, or our *de novo* assemblies of *S. stercoralis* transcripts. We also found that the *Ss-daf-12* locus encoded a total of seven transcripts encoding three different proteins, with the variability confined to the N-terminus of the predicted protein before the DNA-binding domain, similar to that found in *Ce-daf-12* [126], [135].

**Table 4:** Comparison of NHR pathway homologs and transcript abundances in *S. stercoralis* and *C. elegans*.

*C. elegans* gene(s)	*S. stercoralis* homolog(s)	*S. stercoralis* transcript abundance profile	Regulation consistent with *C. elegans* (+, +/−, −)^1^
**Dafachronic acid biosynthesis**			
*Ce-ncr-1* & *-2*	*Ss-ncr-1*	decreased in P Females	+/−
*Ce-daf-36*	*Ss-daf-36*	decreased in L3i and L3+	+/−
*Ce-dhs-16*	*Ss-scdh-16* 2	decreased from L3i to L3+	−
*Ce-daf-9*	*Ss-cyp-9* 2	decreased in FL and P Females	−
*Ce-emb-8*	*Ss-emb-8*	present in all stages examined	+/−
*Ce-strm-1*	*Ss-strm-1*	decreased from L3i to L3+	+
**Nuclear hormone receptor**			
*Ce-daf-12*	*Ss-daf-12*	peak in L3i	+
*Ce-din-1*	Not identified		
***Ce*** **-DAF-12 regulated genes**			
*Ce-gck-2*	*Ss-gck-2*	present in all stages examined	−
*Ce-lev-9*	*Ss-lev-9*	decreased in FL and P Females	−
*Ce-lit-1*	*Ss-lint-1*	decreased in FL and P Females	−
	*Ss-lint-2*	present in all stages examined	−
*Ce-ugt-65*	*Ss-udpgt-1*	not present in L3i or L3+	+
	*Ss-udpgt-2*	low expression, peak in L3i	−

1 (+) similar, (+/−) unclear, and (−) dissimilar transcript abundance patterns.

2 Homology is by phylogenetic similarity only.

**Figure 7:**
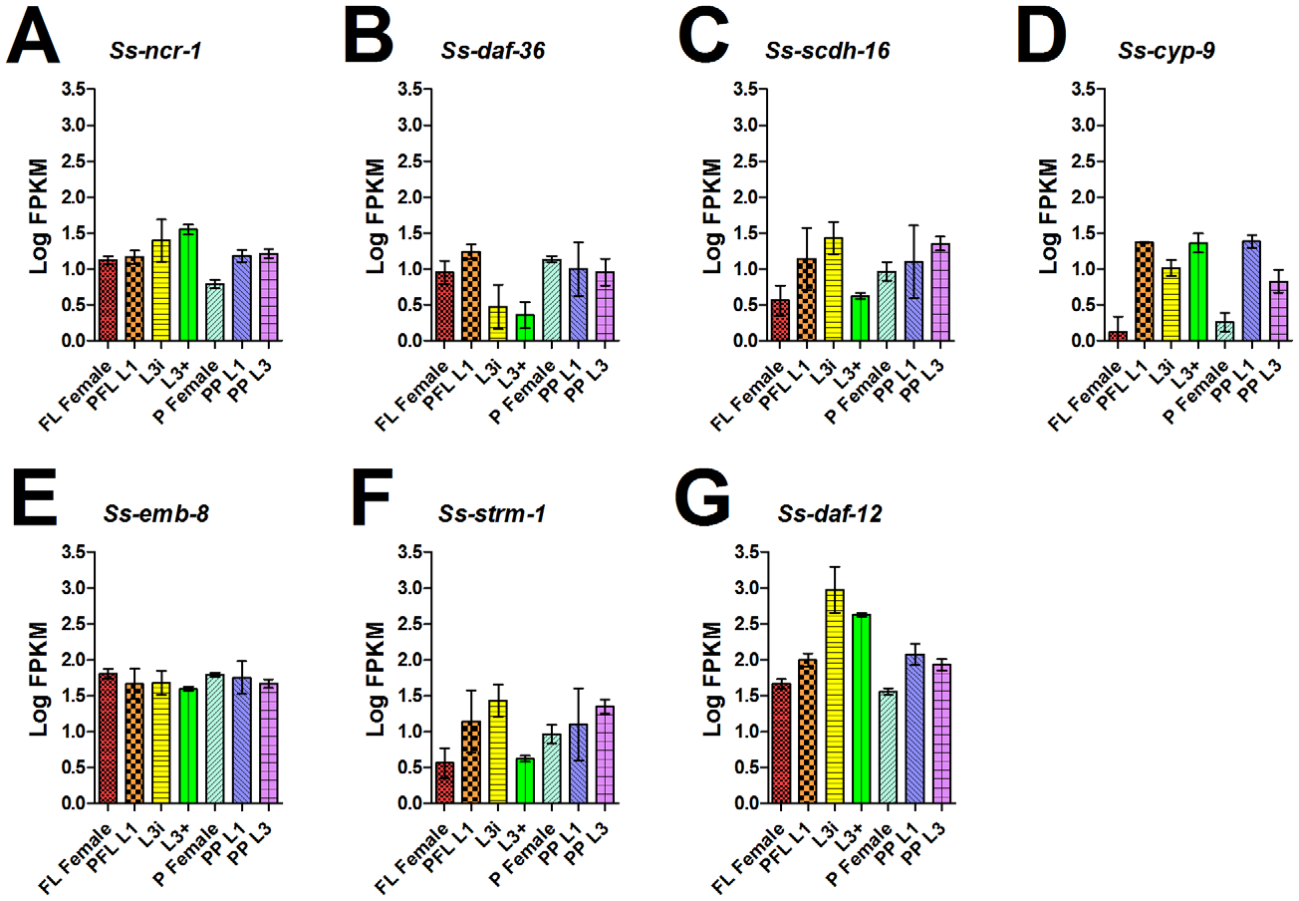
Temporal regulation of *S. stercoralis* DAF-12 and genes putatively involved in dafachronic acid synthesis. Transcript abundances were determined for the coding region of: (A) *Ss-ncr-1*, a gene encoding a homolog of the intracellular cholesterol transporters *Ce*-NCR-1 and *Ce*-NCR-2; (B) *Ss-daf-36*, a gene encoding a homolog of the Rieske-like oxygenase *Ce*-DAF-36; (C) *Ss-scdh-16*, a gene encoding a short-chain dehydrogenase homolog most similar to *Ce*-DHS-16; (D) *Ss-cyp-9*, a gene encoding a cytochrome P450 homolog most similar to *Ce*-DAF-9; (E) *Ss-emb-8*, a gene encoding a homolog of the cytochrome P450 reductase *Ce*-EMB-8; (F) *Ss-strm-1*, a gene encoding a homolog of the methyltransferase *Ce*-STRM-1; and (G) *Ss-daf-12*, the homolog of the nuclear hormone receptor *Ce*-DAF-12. Transcript abundances were quantified in seven developmental stages: free-living females (FL Female), post-free-living first-stage larvae (PFL L1), infectious third-stage larvae (L3i), *in vivo* activated third-stage larvae (L3+), parasitic females (P Female), post-parasitic first-stage larvae (PP L1), and post-parasitic third-stage larvae (PP L3). Transcript abundances were calculated as fragments per kilobase of coding exon per million mapped reads (FPKM) and log transformed. Error bars represent 95% confidence intervals. The y-axes were scaled from 0 to 3.5 to aid comparison between genes.

We then examined the developmental regulation of the *S. stercoralis* genes potentially involved in a DA biosynthetic pathway (Figure 7). We found that *Ss-ncr-1* transcripts peak in L3+ and then significantly decrease in parasitic females (p<0.001), while *Ss-daf-36* transcripts are at their nadir in L3i and L3+ developmental stages. Counterintuitively, we also found that *Ss-cyp-9* transcripts are down-regulated in both free-living and parasitic females compared to the other developmental stages examined. Perhaps our most interesting observation was that *Ss-daf-12* transcript levels peak in L3i and that the differences in expression also reflected significant changes in the promoter usage and coding forms (Figure S9).

### Homologs of *Ce*-DAF-12-regulated genes are not similarly regulated in *S. stercoralis* development

We asked whether homologs of genes transcriptionally regulated by *Ce*-DAF-12 during dauer development were similarly regulated during *S. stercoralis* L3i development. We selected *C. elegans* genes that are directly linked to DAF-12 response elements, are differentially regulated during dauer development [138], and for which we could identify clear homologs in *S. stercoralis* (Table 4). We identified *S. stercoralis* homologs of *Ce-lev-9* and *Ce-gck-2*, which are up-regulated during both dauer induction [138] and following dauer recovery [43], that we termed *Ss-lev-9* and *Ss-gck-2*, respectively. We also identified two *S. stercoralis* homologs of *Ce-lit-1*, which is up-regulated in dauers [138], that we termed *Ss-lint-1* and *Ss-lint-2*. Additionally, we identified two *S. stercoralis* homologs of *Ce-ugt-65*, a gene down-regulated during dauer formation by *Ce*-DAF-12 [138], which we termed *Ss-udpgt-1* and *Ss-udpgt-2*. Intriguingly, we were unable to identify *S. stercoralis* homologs of the *Ce-let-7* microRNA family [139] in the *S. stercoralis* or *S. ratti* draft genomes or in our *de novo* assembled *S. stercoralis* transcripts. Members of this microRNA family are directly regulated by DAF-12 in *C. elegans* and control several dauer developmental programs [140].

We did not observe any consistent regulation of the *S. stercoralis* homologs during L3i formation (Figure S13). In the seven developmental stages examined, *Ss-gck-2* and *Ss-lint-2* had no differences in transcript abundance greater than one log, *Ss-udpgt-1* and *Ss-udpgt-2* were expressed at very low levels in all stages examined, and *Ss-lev-9* and *Ss-lint-1* appeared to have decreased transcripts in parasitic and free-living females in comparison to the other developmental stages. This lack of consistent regulation of target genes between the two species appeared similar to that observed in *S. stercoralis* homologs of genes regulated by *Ce*-DAF-16 in *C. elegans*.

## Discussion

In this study, we determined which homologs of *C. elegans* genes involved in dauer arrest and/or activation (Figure 2) are present in *S. stercoralis* and whether these *S. stercoralis* genes are developmentally regulated in a manner consistent with the regulation of their *C. elegans* counterparts. Our results have provided important insights into which developmental pathways are conserved between the morphologically similar dauer and L3i stages, thereby illuminating potential mechanisms governing L3i development. In our searches of the *S. stercoralis* and *S. ratti* draft genomes as well as our *de novo* assembled *S. stercoralis* transcript database, we were able to identify *S. stercoralis* homologs for nearly every *C. elegans* gene directly involved in the four canonical dauer pathways. While these pathways are well conserved in metazoans, they regulate a wide variety of functions; thus, we were specifically interested in whether they regulate *S. stercoralis* L3i arrest and/or activation.

In previous work, we demonstrated that both IIS and DAs play a role in *S. stercoralis* L3i arrest and activation [10], [22], [136]. However, we have also found that an *S. stercoralis* TGFβ ligand similar to *Ce*-DAF-7 is transcriptionally regulated in opposition to its *C. elegans* homolog [20]. A recent study found that genes involved in dauer recovery differ considerably between the clade V nematodes *Pristionchus pacificus* and *C. elegans* [141], further suggesting potential developmental differences between *S. stercoralis* and *C. elegans*, which are far more evolutionarily divergent [16]. Together, these studies, along with others in multiple parasitic nematode species, have demonstrated that while some *C. elegans* dauer pathway genes and metabolites appear to play a role in L3i development, others appear to be uninvolved [4]. Therefore, in this study, we used an RNAseq approach to globally examine the developmental regulation of *S. stercoralis* homologs in each of the four canonical dauer pathways and to gain key insights into their potential role in regulating *S. stercoralis* L3i development.

### The role of cGMP signaling in *S. stercoralis* development

The pronounced up-regulation in L3i and the striking similarity of the transcriptional profiles of the *S. stercoralis* genes putatively involved in a cGMP signaling pathway (Figure 4) suggest a role in transducing host cues during the infective process. The similar up-regulation of putative guanylyl cyclases that do not phylogenetically group with *Ce*-DAF-11 suggests broad up-regulation of cGMP pathway components in *S. stercoralis* L3i and is reminiscent of studies in *C. elegans* showing that genes with similar temporal regulatory patterns often have similar genetic functions [142]. Since *S. stercoralis* L3i are attracted to chemical and thermal host cues [143]–[145] and are activated in host-like conditions [136], “priming” L3i for infection by up-regulating signaling components that relay these host cues would impart a selective advantage. Therefore, we hypothesize that cGMP signaling plays an important role in transducing signals of host recognition, consistent with studies in *A. caninum*, which demonstrated that a cGMP analog can stimulate L3i activation [66], [146].

This proposed role for cGMP signaling in *S. stercoralis* is somewhat at odds with the role of cGMP pathway signaling in *C. elegans*, where dauer pheromone, composed of a complex mixture of ascarosides, utilizes the cGMP signaling pathway to control dauer arrest [50], [52], [53]. This would suggest that an as yet undiscovered ascaroside helps to control *S. stercoralis* L3i formation. Recent reports suggest that ascarosides play a role in L3i formation in the closely related nematode *P. trichosuri* [68] as well as the entomopathogenic nematode *Heterorhabditis bacteriophora* [67]. Both of these species have multiple free-living generations, allowing ascaroside concentration to build up over time. In contrast, *S. stercoralis* has only one free-living generation, the progeny of which constitutively form L3i regardless of the population density. This makes it difficult to envisage a role for an environmentally secreted ascaroside by either the parasitic or free-living female, although an ascaroside that acts *in utero* on the developing embryo remains a possibility.

### The role of IIS in *S. stercoralis* development

In previous studies, we have demonstrated that *S. stercoralis* IIS is crucial to both L3i arrest [10] and activation [22]. Down-regulation of IIS is necessary for L3i formation, since a *Ss*-DAF-16 dominant interfering construct designed to block the function of native *Ss*-DAF-16 results in L3i bypass phenotypes [10]. Furthermore, up-regulation of IIS is important during L3i activation, since pharmacological inhibition of PI3Ks, which include *Ss*-AGE-1, results in a dramatic decrease in L3i activation [22].

In this study, we demonstrate that the transcripts of intracellular IIS signaling components are always present in all developmental stages examined, with the exception of *Ss-sgk-1* (Figure S8). These results are consistent with findings in *C. elegans*, where IIS signaling is thought to be regulated at the level of the ILPs, while intracellular signaling components are always present [41]. However, we did observe developmental regulation of several IIS signaling component transcripts in the post-free living generation, including increases in the transcript abundances for *Ss-daf-2*, *Ss-age-1*, *Ss-pdk-1*, *Ss-akt-1*, and *Ss-daf-16* (Figure S8). We also noted an absence of *Ss-sgk-1* transcripts in L3i and L3+ (Figure S8). In *C. elegans*, loss of *Ce-sgk-1* results in increased stress resistance and lifespan extension [90]. These two attributes are key features of *S. stercoralis* L3i and we postulate that *Ss-sgk-1* plays a role in these processes. Perhaps our most interesting observation is that *Ss-akt-1b* transcripts, encoding an isoform that is predicted to have a truncated PH domain and may not be subject to regulation by phosphatidylinositol lipids, are found almost exclusively in L3i and L3+ (Figure S9). We hypothesize that *Ss*-AKT-1B modulates *S. stercoralis* IIS during L3i development, potentially by interfering with *Ss*-AKT-1A or its substrates. Together, these data suggest that *S. stercoralis* IIS may be modulated at the level of the intracellular signaling proteins; however, the developmental transcript abundance profiles suggest that the core components are always present.

Upstream regulation of IIS by ILPs has never been demonstrated in parasitic nematodes, and it has generally been assumed that such regulation would be highly complex and redundant, similar to that of *C. elegans*, which has 40 known ILPs [41], [71]. In this study, extensive searches of the *S. stercoralis* and *S. ratti* draft genomes as well as *de novo* assembled *S. stercoralis* transcripts identified only seven ILPs (Figure 5A). These are conserved between these two parasite species but are highly divergent from the ILPs in *C. elegans*. We do not discount the possibility that other ILPs may be present in *S. stercoralis*; however, they would almost certainly have non-canonical cysteine architecture, given our search algorithm. Although we have no direct evidence to support their role in L3i development, we hypothesize that *Ss-ilp-1*, *Ss-ilp-6*, and *Ss-ilp-7* encode ligands that regulate *S. stercoralis* IIS during L3i development. Determining whether an ILP acts as an agonist or antagonist is complicated by the fact that IIS regulates functions other than dauer development in *C. elegans*, including life-span [76].

We hypothesize that *Ss-ilp-7* encodes a type γ antagonistic IIS ligand that promotes developmental arrest, due to the conservation of a unique PPG motif found in *Ce*-INS-18, which acts as an IIS antagonist in *C. elegans* [71], [81]. This hypothesis is supported by our observation that *Ss-ilp-7* transcripts are significantly up-regulated in the post-free-living generation and peak in L3i, which are developmentally arrested (Figure 5H). However, *Ss-ilp-7* transcripts remain at an elevated level in L3+, which are developmentally activated. The similar levels of *Ss-ilp-7* transcripts in L3i and L3+ may reflect the fact that both forms are third-stage larvae and that the L3+ has yet to complete all the developmental programs associated with activation, which may only commence after molting and establishment in the intestine.

We also hypothesize that *Ss-ilp-1* encodes an agonistic ligand that, when down-regulated, allows parasitic development, as it is the only other gene we identified to encode a type γ ILP, a family that also includes the human agonists insulin and IGF-1 [71]. The fact that *Ss-ilp-1* transcripts are significantly down-regulated in L3i, L3+, and parasitic females supports this characterization (Figure 5B). Continued down-regulation of *Ss-ilp-1* transcripts in L3+ and parasitic females is difficult to reconcile with a strictly developmental regulatory role in L3i. However, it should be noted that parasitic females retain characteristics of L3i, including an extended lifespan. Parasitic females live for many months in contrast to a lifespan of a few days for their free-living counterparts [147]. We hypothesize that negative regulation of lifespan by IIS is reversed in the long-lived parasitic forms and that *Ss*-ILP-1 participates in this effect.

We also speculate that *Ss-ilp-6* encodes an agonistic ligand that promotes larval growth and development in both homogonic and heterogonic phases of the life cycle. This is based on our observation that *Ss-ilp-6* transcript levels decrease during post-free-living development, reaching a minimum in physiologically older L3i, and then increase by one log in L3+, which have resumed feeding (Figure S7). Additionally, *Ss-ilp-6* transcripts appear to increase in rapidly developing post-parasitic larvae (Figure 5G).

In future studies, we will test whether these three *S. stercoralis* ILPs, and possibly others, directly regulate L3i development. Due to the significantly smaller number of ILPs in *S. stercoralis* compared to *C. elegans*, as well as their highly divergent amino acid sequences and even novel cysteine architecture, we believe that these two species differ in the diversity and complexity of ligands regulating the DAF-2 receptor. Nevertheless, the data we report here are consistent with roles for *S. stercoralis* ILPs in the regulation of larval development and lifespan via the IIS pathway.

Differences in IIS regulated genes between *S. stercoralis* and *C. elegans* are suggested by our observation that none of the *S. stercoralis* homologs of *Ce*-DAF-16-regulated genes we examined, including *Ss-sod-1* and *Ss-daf-15*, had transcript abundance differences greater than one log between any of the *S. stercoralis* developmental stages examined (Figure S10). We found this observation interesting because *Ss*-DAF-16 can heterologously complement *C. elegans daf-16* mutants [19], suggesting similar biochemical capabilities. These results illustrate the important caveat that heterologous rescue does not prove that homologous genes fulfill similar genetic functions. However, no *Ce*-DAF-16-regulated genes have been shown to be “master regulators” of dauer development, and thus it is difficult to determine which target genes are most important. *Ce*-DAF-16 regulates several biological processes in addition to dauer development, including longevity, stress responses, and metabolism, and this has complicated the identification of target genes for specific processes [98]. The *S. stercoralis* genes in this study were selected because in *C. elegans*, the homologs are transcriptionally regulated directly by *Ce*-DAF-16 during dauer development and have dauer-associated phenotypes upon RNAi knock-down of their transcripts [98]. Our previous work points to *S. stercoralis* IIS regulating L3i arrest and activation, but the genes regulated by *Ss*-DAF-16 to carry out this process are unknown. In future studies, we hope to determine which *S. stercoralis* genes are regulated by *Ss*-DAF-16 using a chromatin immunoprecipitation and deep sequencing (ChIPseq) approach by constructing a stable transgenic *S. stercoralis* line that expresses a tagged version of *Ss*-DAF-16 [148].

### The role of dauer-like TGFβ signaling in *S. stercoralis* development

Previously, we identified an *S. stercoralis* TGFβ ligand similar to *Ce*-DAF-7 [20], a finding repeated in two closely related parasitic nematodes [23]. In contrast to *C. elegans*, these putative TGFβ ligand-encoding genes are up-regulated in L3i, while *Ce-daf-7* is down-regulated in the dauer stage [23]. In this study, we identified six additional *S. stercoralis* genes encoding TGFβ1 family ligands similar to *Ce*-DAF-7 (Figure 6A), which is the only dauer pathway TGFβ ligand in *C. elegans* [42], [102]. We were not only surprised by the increase in the number of genes encoding *Ce*-DAF-7-like ligands in *S. stercoralis*, but also by their temporal regulation.

We noted that *Ss-tgh-1*, *Ss-tgh-2*, and *Ss-tgh-3* transcripts are found exclusively in L3i (Figure 6B–D), suggesting a similar function. As previously proposed, the *S. stercoralis* TGFβ-like ligands encoded by *Ss-tgh-1*, *Ss-tgh-2*, and *Ss-tgh-3* may play a role in L3i arrest [23] or may be stored in L3i and secreted into the host following activation for purposes of immunomodulation [106]. Recent work has shown that *H. polygyrus* excretory-secretory antigen binds and activates the host TGFβ receptor, potentially supporting an immunomodulatory role for nematode TGFβ ligands [149]. Additionally, we noted highly variable transcript abundances for these three genes, as well as several others, in the L3i developmental stage, evidenced by the large 95% confidence intervals. Since we isolated L3i incubated at 21°C or 25°C and after 7, 8, or 10 days of culture (Data S11), we plotted the transcript abundances for the genes encoding both the ILP and TGH ligands over the course of post-free-living larval development, with each L3i biological replicate plotted by relative age (Figure S7). We observed that the large 95% confidence intervals were not stochastic, but rather represented underlying developmental trends dependent upon the relative age of the L3i. Therefore, we concluded that L3i may not be the static population originally assumed. Instead, physiologic age of developmentally arrested L3i, which is a function of temperature and time, may influence the transcriptomic profile of a synchronous population of these infectious larvae. These observations lead us to favor the hypothesis that the up-regulation of *Ss-tgh-1*, *Ss-tgh-2*, and *Ss-tgh-3* during L3i development may play a role in L3i arrest; however, this role is not mutually exclusive with immunomodulation, given the plurality of TGH ligands in *S. stercoralis*.

In this study, we also identified four other genes encoding *Ce*-DAF-7-like ligands (Figure 6A). Both *Ss-tgh-4* and *Ss-tgh-5* transcripts were not detected in any developmental stage examined (Figure 6E–F), while *Ss-tgh-6* and *Ss-tgh-7* transcripts encoded putative peptides without a pro-peptide domain. We do not know whether *Ss-tgh-4* or *Ss-tgh-5* are ever robustly expressed during *S. stercoralis* development or are pseudo-genes; however, two important developmental stages, free-living males and auto-infective L3, were absent from this study. Additionally, the function of the putative ligands encoded by *Ss-tgh-6* and *Ss-tgh-7* are altogether unknown. Advances in the technologies to knock down genes by RNAi in *S. stercoralis*, which to date has been intractable to this approach, would facilitate our ability to address these questions [150].

Examination of intracellular signaling components of the dauer TGFβ pathway in *S. stercoralis* also led to some perplexing observations. While we were able to identify clear *S. stercoralis* homologs of genes encoding the Type I and Type II dauer pathway TGFβ receptors, *Ss-daf-1* and *Ss-daf-4*, as well as the homolog of the *Ce*-DAF-5 transcriptional co-factor, *Ss*-DAF-5, we were unable to clearly identify homologs of the SMADs *Ce*-DAF-8, *Ce*-DAF-14, and *Ce*-DAF-3 (Figure S4). The large protein sequence divergence of dauer pathway TGFβ SMADs in *S. stercoralis* and all other sequenced nematodes, evidenced by our inability to resolve them phylogenetically, indicates a high degree of evolutionary divergence and suggests the potential for rapid evolution of these genes. This is in stark contrast to the SMADs in the Sma/Mab TGFβ pathway, for which clear sequence-level relationships exist for all nematode species examined (Figure S4). However, future testing of the functional consequences of this sequence-level divergence of dauer pathway TGFβ SMADs will be challenging due to the current limitations of functional genomic methods in *S. stercoralis*.

### The regulation of dafachronic acid biosynthesis and DAF-12 in *S. stercoralis* development

We previously demonstrated that Δ^7^-DA is a potent activator of L3i and can act as a ligand for the nuclear hormone-receptor *Ss*-DAF-12 [136]. Furthermore, Δ^7^-DA can promote L3i bypass phenotypes in the post-free-living generation [136]. From these observations, we hypothesized that *S. stercoralis* synthesizes DAs *in vivo* and that the homologs of the enzymes responsible for DA biosynthesis would be up-regulated during reproductive development and down-regulated in L3i. This would also be consistent with observations in *C. elegans*, where *Ce-daf-9* and *Ce-daf-36* are up-regulated during dauer recovery [43].

Contrary to our hypothesis, we did not observe consistent developmental regulation of *S. stercoralis* homologs putatively involved in DA biosynthesis (Figure 7). Most puzzling was the significant decrease in *Ss-cyp-9* transcripts, which encode a putative cytochrome P450 most similar to *Ce*-DAF-9, in both free-living and parasitic females (Figure 7D). Although *Ss-daf-36* transcripts, which encode a putative Rieske-like oxygenase, appeared to be decreased in L3i and L3+ in comparison to other developmental stages, there was no significant difference between L3i and L3+ (Figure 7B). We expected both *Ss-cyp-9* and *Ss-daf-36* transcripts to be down-regulated in the developmentally arrested L3i and up-regulated in activated L3+; however, the L3+ may not have fully initiated all programs associated with resumption of development, as discussed for the ILPs. Only *Ss-strm-1*, the homolog of which in *C. elegans* encodes a methyltransferase that decreases DA levels when active, was down-regulated from L3i to L3+ and consistent with our hypothesis (Figure 7F). This inconsistent regulation of putative DA biosynthetic enzymes may be a result of our misidentification of several enzymes, such as *Ss-cyp-9* and *Ss-scdh-16*, of which several closely related homologs are present in *S. stercoralis* (Figures S5 and S6). Additionally, these inconsistent results may be a result of additional layers of regulation which await discovery. In future studies, we hope to verify the role of these enzymes in a DA biosynthetic pathway.

Interestingly, we noted that unlike *Ce*-DAF-12, which is down-regulated in the dauer stage [126], *Ss-daf-12* transcripts were at their peak in L3i (Figure 7G) and that this up-regulation was isoform specific (Figure S9). The differences in promoter usage as well as the predicted differences in the N-terminus of *Ss*-DAF-12 may represent additional layers of regulation. Until the native *Ss*-DAF-12 ligand(s) are identified and quantified for each developmental stage in future studies, the endogenous role of DAs in *S. stercoralis* development will remain difficult to assess.

Transcriptional regulation of *S. stercoralis* homologs of *Ce*-DAF-12-regulated genes was more difficult to interpret than for the *S. stercoralis* homologs of *Ce*-DAF-16-regulated genes. While some genes, including *Ss-lev-9* and *Ss-lint-1*, appeared to be regulated similarly to *Ss-cyp-9*, others, including *Ss-gck-2* and *Ss-lint-2*, did not display substantial changes in their transcript levels in the developmental stages examined (Figure S13). Our inability to identify *S. stercoralis* homologs of the *Ce-let-7* miRNA family, which in *C. elegans* are directly regulated by *Ce*-DAF-12 to control development [140], further confounded our ability to interpret these results. In future studies, a ChIPseq approach using a tagged *Ss*-DAF-12 construct expressed in a stable transgenic *S. stercoralis* line would allow for the identification of native *Ss*-DAF-12-regulated genes in different developmental stages [148].

### Putative mechanisms controlling *S. stercoralis* L3i development

Our data, as well as that from previous studies in other parasitic nematodes, point to several key regulatory pathways that may govern *S. stercoralis* L3i arrest and activation. The potent up-regulation of many cGMP signaling pathway components in L3i (Figure 4) is striking. We hypothesize that this pathway is directly involved in sensing/transducing host cues when L3i encounter a favorable host.

Perhaps the most interesting observation in this study is the paucity of *S. stercoralis* ILPs in comparison to *C. elegans* and the fact that several of these ILP encoding genes are dramatically up-/down-regulated during the course of L3i development (Figure 5). These data support a role for ILPs in regulating L3i arrest by modulating IIS, in agreement with our previous findings that *Ss*-DAF-16 regulates L3i arrest [10] and that *S. stercoralis* PI3Ks play a role in L3i activation [22]. We hypothesize that both L3i arrest in the environment and activation in the host are functions of the balance between agonistic and antagonistic ILPs. Under this hypothesis, down-regulation of agonistic ILPs and up-regulation of antagonistic ILPs would drive L3i arrest, while the reciprocal balance of ILPs would stimulate L3i activation and resumption of development in the host.

We were surprised by the increased number of genes encoding *S. stercoralis* TGFβ ligands similar to the single *C. elegans* dauer TGFβ ligand and the fact that three of these are expressed solely in L3i (Figure 6). As previously proposed [4], we hypothesize that these *Ce*-DAF-7-like TGFβ ligands play an important role in regulating L3i arrest. It is also possible that these TGFβ ligands may modulate host immunity.

Our lab and others have demonstrated that DAs are potent activators of L3i as well as stimulators of heterogonic development [136], [137]. However, the transcriptional profiles of the *S. stercoralis* DA biosynthetic enzyme homologs identified in this study do not demonstrate any coordinated regulation (Figure 7). Careful dissection of this pathway in future studies will be needed to determine the *in vivo* role of DA biosynthesis and *Ss*-DAF-12 regulation during L3i activation and heterogonic development. Together, these four pathways present several exciting avenues of future research in understanding the mechanisms controlling *S. stercoralis* L3i arrest and activation. Additionally, the transcriptomic data that formed the basis of this study provide a rich source of information for future unbiased global surveys of genes differentially regulated during L3i development and for many other aspects of parasitic nematode biology.

## Supporting Information

Figure S1 **Morphological comparison of** ***S. stercoralis*** **L3i and L3+.** *S. stercoralis* third-stage larvae were photographed as (A) developmentally arrested and non-feeding third-stage larvae (L3i) or (B) as feeding third-stage larvae activated *in vivo* for three days in a permissive host (L3+). Overall, the body of the (A) L3i is more radially constricted than that of the (B) L3+. Similarly, the (A) L3i has a filariform pharynx (f) that is radially constricted forming a long thin tube extending almost to the midpoint of the worm, while the (B) L3+ has a pharynx that is pumping, is not radially constricted, and has differentiated into both muscular (m) and glandular(g) segments. The fork-shaped tail (arrow) in both the (A) L3i and (B) L3+ is characteristic of third-stage larvae in *Strongyloides* species and is lost in subsequent larval stages. Scale bar represents 100 µM.
(TIF)

Figure S2 **Phylogenetic analysis of phylum Nematoda guanylyl cyclases similar to** ***Ce*** **-DAF-11.** A protein alignment, generated with Clustal W, of several predicted guanylyl cyclases similar to *Ce*-DAF-11 from *S. stercoralis* and other parasitic nematodes was used to construct a neighbor-joining tree with 100 iterations of boot-strapping. The predicted *Ss*-GCY-11 protein grouped with *Ce*-DAF-11. Abbreviations: *Ascaris suum* (As), *Brugia malayi* (Bm), *Caenorhabditis briggsae* (Cb), *Caenorhabditis elegans* (Ce), *Heterodera glycines* (Hg), *Loa loa* (Ll), *Strongyloides stercoralis* (Ss), and *Trichinella spiralis* (Ts). The scale bar represents substitutions per position. Accession numbers are listed in Data S4.
(TIF)

Figure S3 **Protein alignment of TGFβ ligand domains by cysteine architecture.** Protein sequence alignment of TGFβ ligand domains including all cysteine residues in *S. stercoralis* and other metazoans revealed three distinct sets of cysteine architecture: *Hs*-TGFβ1-like ligands, including *Ce*-DAF-7, which have nine conserved cysteine residues; *Drosophila melanogaster* decapentaplegic (DPP) and vertebrate bone morphogenetic protein (BMP) -like ligands, including *Ce*-DBL-1 and *Ce*-UNC-129 subfamilies, which have seven conserved cysteine residues; and *Ce*-TIG-2-like ligands, which have six conserved cysteine residues. The conserved cysteine residues, critical for disulfide bond formation and “cysteine knot” folding, are in red. Abbreviations: *Ancylostoma caninum* (Ac), *Ascaris suum* (As), *Brugia malayi* (Bm), *Caenorhabditis briggsae* (Cb), *Caenorhabditis elegans* (Ce), *Danio rerio* (Dr), *Drosophila melanogaster* (Dm), *Haemonchus contortus* (Hc), *Heligmosomoides polygyrus* (Hp), *Homo sapiens* (Hs), *Loa loa* (Ll), *Parastrongyloides trichosuri* (Pt), *Strongyloides ratti* (Sr), *Strongyloides stercoralis* (Ss), *Trichinella spiralis* (Ts), and *Xenopus laevis* (Xl). Accession numbers are listed in Data S4.
(TIF)

Figure S4 **Phylogenetic analysis of phylum Nematoda SMAD homologs.** A protein alignment, generated with Clustal W, of all publicly available phylum Nematoda SMAD homolog predicted proteins, was used to construct a neighbor-joining tree with 100 iterations of boot-strapping. The small body size and male tail abnormal (Sma/Mab) TGFβ pathway SMADs, including *Ce*-SMA-2, *Ce*-SMA-3, and *Ce*-SMA-4, resolved into distinct clades while the dauer TGFβ pathway SMADs, including *Ce*-DAF-3, *Ce*-DAF-8, and *Ce*-DAF-14, did not. Abbreviations: *Ascaris suum* (As), *Brugia malayi* (Bm), *Caenorhabditis briggsae* (Cb), *Caenorhabditis elegans* (Ce), *Drosophila melanogaster* (Dm), *Heterodera glycines* (Hg), *Homo sapiens* (Hs), *Loa loa* (Ll), *Strongyloides stercoralis* (Ss), and *Trichinella spiralis* (Ts). The scale bar represents substitutions per position. Accession numbers are listed in Data S4.
(TIF)

Figure S5 **Phylogenetic analysis of phylum Nematoda short-chain dehydrogenase homologs similar to** ***Ce*** **-DHS-16.** A protein alignment, generated with Clustal W, of phylum Nematoda short-chain dehydrogenase homologs similar to *Ce*-DHS-16 was used to construct a neighbor-joining tree with 100 iterations of boot-strapping. The predicted protein for *Ss*-SCDH-16 grouped closest to *Ce*-DHS-16. Abbreviations: *Ascaris suum* (As), *Brugia malayi* (Bm), *Caenorhabditis briggsae* (Cb), *Caenorhabditis elegans* (Ce), *Loa loa* (Ll), *Strongyloides stercoralis* (Ss), and *Trichinella spiralis* (Ts). The scale bar represents substitutions per position. Accession numbers are listed in Data S4.
(TIF)

Figure S6 **Phylogenetic analysis of phylum Nematoda cytochrome P450 homologs similar to** ***Ce*** **-DAF-9.** A protein alignment, generated with Clustal W, of phylum Nematoda cytochrome P450 homologs similar to *Ce*-DAF-9 was used to construct a neighbor-joining tree with 100 iterations of boot-strapping. The predicted protein for *Ss*-CYP-9 grouped closest to *Ce*-DAF-9. Abbreviations: *Ascaris suum* (As), *Brugia malayi* (Bm), *Bursaphelenchus xylophilus* (Bx), *Caenorhabditis briggsae* (Cb), *Caenorhabditis elegans* (Ce), *Drosophila melanogaster* (Dm), *Homo sapiens* (Hs), *Loa loa* (Ll), *Strongyloides stercoralis* (Ss), and *Trichinella spiralis* (Ts). The scale bar represents substitutions per position. Accession numbers are listed in Data S4.
(TIF)

Figure S7 **Regulation of ILP and TGFβ ligand genes during post-free-living development.** Transcript abundances were determined for the coding region of (A–G) seven *S. stercoralis* insulin-like peptide (ILP) -encoding genes (*Ss-ilp-1* through *-7*) and (H–N) seven *S. stercoralis* DAF-7-like TGFβ ligand encoding genes (*Ss-tgh-1* through *-7*) over the course of post-free living larval development. Transcript abundances were examined for post-free-living first-stage larvae (PFL L1), infectious third-stage larvae (L3i), and *in vivo* activated third-stage larvae (L3+). The mean of both PFL L1 and L3+, from the three biological replicates, was plotted along with the 95% confidence intervals. Each biological sample of L3i was plotted individually according to relative age: L3i incubated at 21°C for 8 days (young L3i), L3i incubated at 25°C for 7 days (medium L3i), and L3i incubated at 21°C for 10 days (old L3i). Transcript abundances were calculated as fragments per kilobase of coding exon per million mapped reads (FPKM) and log transformed. The y-axes were scaled from 0 to 3.5 to aid comparison between genes.
(TIF)

Figure S8 **Developmental regulation of** ***S. stercoralis*** **homologs of intracellular IIS genes.** Transcript abundances were determined for the coding region of *S. stercoralis* homologs of genes encoding insulin/IGF-1-like signaling (IIS) pathway proteins, including: (A) *Ss-asna-1*, a homolog of *Ce-asna-1*, which encodes a putative membrane transporter involved in insulin-like peptide secretion; (B) *Ss-daf-2*, a homolog of *Ce-daf-2*, which encodes an insulin-like receptor; (C) *Ss-ist-1* and (D) *Ss-ist-2*, homologs of the insulin receptor substrate encoding gene *Ce-ist-1*; (E) *Ss-aap-1*, a homolog of *Ce-aap-1*, which encodes a phosphatidylinositol-3 (PI3) kinase accessory/regulatory subunit; (F) *Ss-age-1*, a homolog of *Ce-age-1*, which encodes a PI3 kinase catalytic subunit; (G) *Ss-pten-1* and (H) *Ss-pten-2*, homologs of *Ce-daf-18*, which encodes a phosphatase opposing *Ce-age-1* function; (I) *Ss-pdk-1*, a homolog of *Ce-pdk-1*, which encodes a PTEN-like kinase; (J) *Ss-sgk-1*, a homolog of the serum- and glucocorticoid-inducible kinase *Ce-sgk-1*; (K) *Ss-akt-1*, a homolog of *Ce-akt-1* and *-2*, which encode AKT kinases; (L) *Ss-pptr-1*, a homolog of *Ce-pptr-1*, which encodes a B56 regulatory subunit of the PP2A phosphatase opposing *Ce*-AKT-1 function; (M) *Ss-ftt-2*, a homolog of the 14-3-3 encoding gene *Ce-ftt-2*; and (N) *Ss-daf-16*, a homolog of *Ce-daf-16*, which encodes a forkhead transcription factor. Transcript abundances were quantified in seven developmental stages: free-living females (FL Female), post-free-living first-stage larvae (PFL L1), infectious third-stage larvae (L3i), *in vivo* activated third-stage larvae (L3+), parasitic females (P Female), post-parasitic first-stage larvae (PP L1), and post-parasitic third-stage larvae (PP L3). Transcript abundances were calculated as fragments per kilobase of coding exon per million mapped reads (FPKM) and log transformed. Error bars represent 95% confidence intervals. The y-axes were scaled from 0 to 3.5 to aid comparison between genes.
(TIF)

Figure S9 **Developmental stage-specific transcripts of** ***S. stercoralis akt-1*** **,** ***daf-16*** **, and** ***daf-12*** **isoforms.** (A, B) Two transcripts, each from different promoters, are generated from the *Ss-akt-1* locus: (A) *Ss-akt-1a* and (B) *Ss-akt-1b*. The *Ss-akt-1b* transcript encodes a putative 525 amino acid *Ss*-AKT-1B peptide that has 33 amino acids truncated from the conserved AKT pleckstrin homology (PH) domain at the N-terminus. The *Ss-akt-1a* transcript encodes a putative 580 amino acid *Ss*-AKT-1A protein with a conserved full-length PH domain. (C, D) The locus from which the *S. stercoralis* forkhead transcription factor *Ss-daf-16* is transcribed also produces two transcripts, (C) *Ss-daf-16a* and (D) *Ss-daf-16b*, each from a different promoter. These transcripts encode a 741 amino acid *Ss*-DAF-16A predicted protein and a 566 amino acid *Ss*-DAF-16B predicted protein, which differ at the N-terminus. (E–G) The genomic locus encoding the *S. stercoralis* nuclear hormone receptor homolog *Ss*-DAF-12 expresses at least seven different transcripts, which we termed *Ss-daf-12a-g*, from several promoters. These seven transcripts encode a total of three predicted proteins, each differing at the N-terminus before the DNA-binding domain, with transcripts (E) *Ss-daf-12a,d,f* encoding a putative 752 amino acid *Ss*-DAF-12A protein, (F) *Ss-daf-12b* encoding a putative 947 amino acid *Ss*-DAF-12B protein, and (G) *Ss-daf-12c,e,g* encoding a putative 722 amino acid *Ss*-DAF-12C protein. Transcript abundances were quantified in seven developmental stages: free-living females (FL Female), post-free-living first-stage larvae (PFL L1), infectious third-stage larvae (L3i), *in vivo* activated third-stage larvae (L3+), parasitic females (P Female), post-parasitic first-stage larvae (PP L1), and post-parasitic third-stage larvae (PP L3). Transcript abundances were calculated as fragments per kilobase of transcript exon per million mapped reads (FPKM) and log transformed. Error bars represent 95% confidence intervals. The y-axes were scaled from 0 to 3.5 to aid comparison between genes.
(TIF)

Figure S10 **Lack of developmental regulation of** ***S. stercoralis*** **homologs of** ***Ce*** **-DAF-16-regulated genes.** Transcript abundances were examined for *S. stercoralis* homologs of *C. elegans* genes that are transcriptionally regulated directly by *Ce*-DAF-16 and have a phenotype associated with RNAi knock-down. (A) *Ss-sod-1* is the sole *S. stercoralis* superoxide dismutase and is a homolog of *Ce-sod-3*. (B) *Ss-daf-15* and (C) *Ss-acs-19* are homologs of *Ce-daf-15* and *Ce-acs-19*, respectively. Both (D) *Ss-limdb-1* and (E) *Ss-limdb-2* are homologs of *Ce-lim-1*, while (F) *Ss-pitp-1* and (G) *Ss-Y105E8B.9* are homologs of *Ce-pitp-1* and *Ce-Y105E8B.9*, respectively. Transcript abundances were quantified in seven developmental stages: free-living females (FL Female), post-free-living first-stage larvae (PFL L1), infectious third-stage larvae (L3i), *in vivo* activated third-stage larvae (L3+), parasitic females (P Female), post-parasitic first-stage larvae (PP L1), and post-parasitic third-stage larvae (PP L3). Transcript abundances were calculated as fragments per kilobase of coding exon per million mapped reads (FPKM) and log transformed. Error bars represent 95% confidence intervals. The y-axes were scaled from 0 to 3.5 to aid comparison between genes.
(TIF)

Figure S11 **Developmental regulation of additional** ***S. stercoralis*** **TGFβ ligand transcripts.** The *S. stercoralis* genome contains three TGFβ super-family ligands that do not group with the human TGFβ1 and *Ce*-DAF-7 family: (A) *Ss-tigl-1*, a gene encoding a homolog of *Ce*-TIG-2, a TGFβ ligand that does not have any known biological function or receptor; (B) *Ss-dbl-1*, a gene encoding a homolog of *Ce*-DBL-1, a TGFβ ligand homolog of *D. melanogaster* decapentaplegic (DPP) and vertebrate bone morphogenetic protein (BMP), which signals through the small body size and male tail abnormal (Sma/Mab) pathway; and (C) *Ss-dbl-2*, a gene encoding a homolog of the *Ce*-UNC-129 TGFβ ligand that regulates cell migration and axon guidance and acts independently of type I or type II receptors. Transcript abundances were quantified in seven developmental stages: free-living females (FL Female), post-free-living first-stage larvae (PFL L1), infectious third-stage larvae (L3i), *in vivo* activated third-stage larvae (L3+), parasitic females (P Female), post-parasitic first-stage larvae (PP L1), and post-parasitic third-stage larvae (PP L3). Transcript abundances were calculated as fragments per kilobase of coding exon per million mapped reads (FPKM) and log transformed. Error bars represent 95% confidence intervals. The y-axes were scaled from 0 to 3.5 to aid comparison between genes.
(TIF)

Figure S12 **Developmental regulation of TGFβ signaling homologs in** ***S. stercoralis*** **.** Developmental expression patterns were assessed for members of both the dauer and small body size and male tail abnormal (Sma/Mab) TGFβ pathways: (A) *Ss-daf-4*, a gene encoding a homolog of the type II receptor *Ce*-DAF-4; (B) *Ss-sma-6*, a gene encoding a homolog of the Sma/Mab type I receptor *Ce*-SMA-6; (C) *Ss-daf-1*, a gene encoding a homolog of the dauer type I receptor *Ce*-DAF-1; (D) *Ss-bra-1*, a gene encoding a homolog of the *Ce*-DAF-1 negative regulator *Ce*-BRA-1; (E) *Ss-smad-1*, a gene encoding a SMAD similar to *Ce*-DAF-14; (F) *Ss-smad-2*, (G) *Ss-smad-3*, and (H) *Ss-smad-4*, genes encoding SMAD homologs similar to *Ce*-SMA-2, *Ce*-SMA-3, and *Ce*-SMA-4, respectively; (I) *Ss-smad-5*, (J) *Ss-smad-7*, and (K) *Ss-smad-8*, genes encoding SMAD homologs similar to *Ce*-DAF-3 and *Ce*-DAF-8; (L) *Ss-pdp-1*, a gene encoding a homolog of the phosphatase *Ce*-PDP-1; (M) *Ss-sma-9*, a gene encoding a homolog of the Sma/Mab transcriptional co-factor *Ce*-SMA-9; and (N) *Ss-daf-5*, a gene encoding a Sno/Ski transcription factor homolog similar to *Ce*-DAF-5. Transcript abundances were quantified in seven developmental stages: free-living females (FL Female), post-free-living first-stage larvae (PFL L1), infectious third-stage larvae (L3i), *in vivo* activated third-stage larvae (L3+), parasitic females (P Female), post-parasitic first-stage larvae (PP L1), and post-parasitic third-stage larvae (PP L3). Transcript abundances were calculated as fragments per kilobase of coding exon per million mapped reads (FPKM) and log transformed. Error bars represent 95% confidence intervals. The y-axes were scaled from 0 to 3.5 to aid comparison between genes.
(TIF)

Figure S13 **Developmental regulation of** ***S. stercoralis*** **homologs of** ***Ce*** **-DAF-12-regulated genes.** Transcript abundances of *S. stercoralis* homologs of *C. elegans* genes transcriptionally regulated directly by *Ce*-DAF-12 during dauer development were examined: (A) *Ss-gck-2*, a gene encoding a homolog of *Ce*-GCK-2; (B) *Ss-lev-9*, a gene encoding a homolog of *Ce*-LEV-9; (C) *Ss-lint-1* and (D) *Ss-lint-2*, genes encoding homologs of *Ce*-LIT-1; and (E) *Ss-udpgt-1* and (F) *Ss-udpgt-2*, genes encoding homologs of *Ce*-UGT-65. Transcript abundances were quantified in seven developmental stages: free-living females (FL Female), post-free-living first-stage larvae (PFL L1), infectious third-stage larvae (L3i), *in vivo* activated third-stage larvae (L3+), parasitic females (P Female), post-parasitic first-stage larvae (PP L1), and post-parasitic third-stage larvae (PP L3). Transcript abundances were calculated as fragments per kilobase of coding exon per million mapped reads (FPKM) and log transformed. Error bars represent 95% confidence intervals. The y-axes were scaled from 0 to 3.5 to aid comparison between genes.
(TIF)

Data S1 ***Strongyloides stercoralis*** **dauer homolog genome annotations.**
(GFF3)

Data S2 ***Strongyloides stercoralis*** **dauer homolog transcript sequences.**
(FASTA)

Data S3 ***Strongyloides stercoralis*** **dauer homolog predicted protein sequences.**
(FASTA)

Data S4 **Accession numbers for protein alignments.**
(XLS)

Data S5 **Protein alignment for guanylyl cyclases similar to** ***Ce*** **-DAF-11.**
(NEX)

Data S6 **Protein alignment for truncated TGFβ ligand domains.**
(NEX)

Data S7 **Protein alignment for phylum Nematoda SMADs.**
(NEX)

Data S8 **Protein alignment for short-chain dehydrogenases similar to** ***Ce*** **-DHS-16.**
(NEX)

Data S9 **Protein alignment for cytochrome P450s similar to** ***Ce*** **-DAF-9.**
(NEX)

Data S10 **FPKM values for** ***Strongyloides stercoralis*** **genes and isoforms.**
(XLS)

Data S11 **RNAseq library preparation conditions and alignment statistics.**
(XLS)

Text S1 **Supplemental methods for isolation of** ***Strongyloides stercoralis*** **developmental stages.**
(DOC)
