# Supplementary figures and images for: RNAseq Analysis of the Parasitic Nematode Strongyloides stercoralis Reveals Divergent Regulation of Canonical Dauer Pathways
Source: PLoS Negl Trop Dis. 2012 Oct 25;6(10):e1854. doi: 10.1371/journal.pntd.0001854 (PMC3493385; doi:10.1371/journal.pntd.0001854)

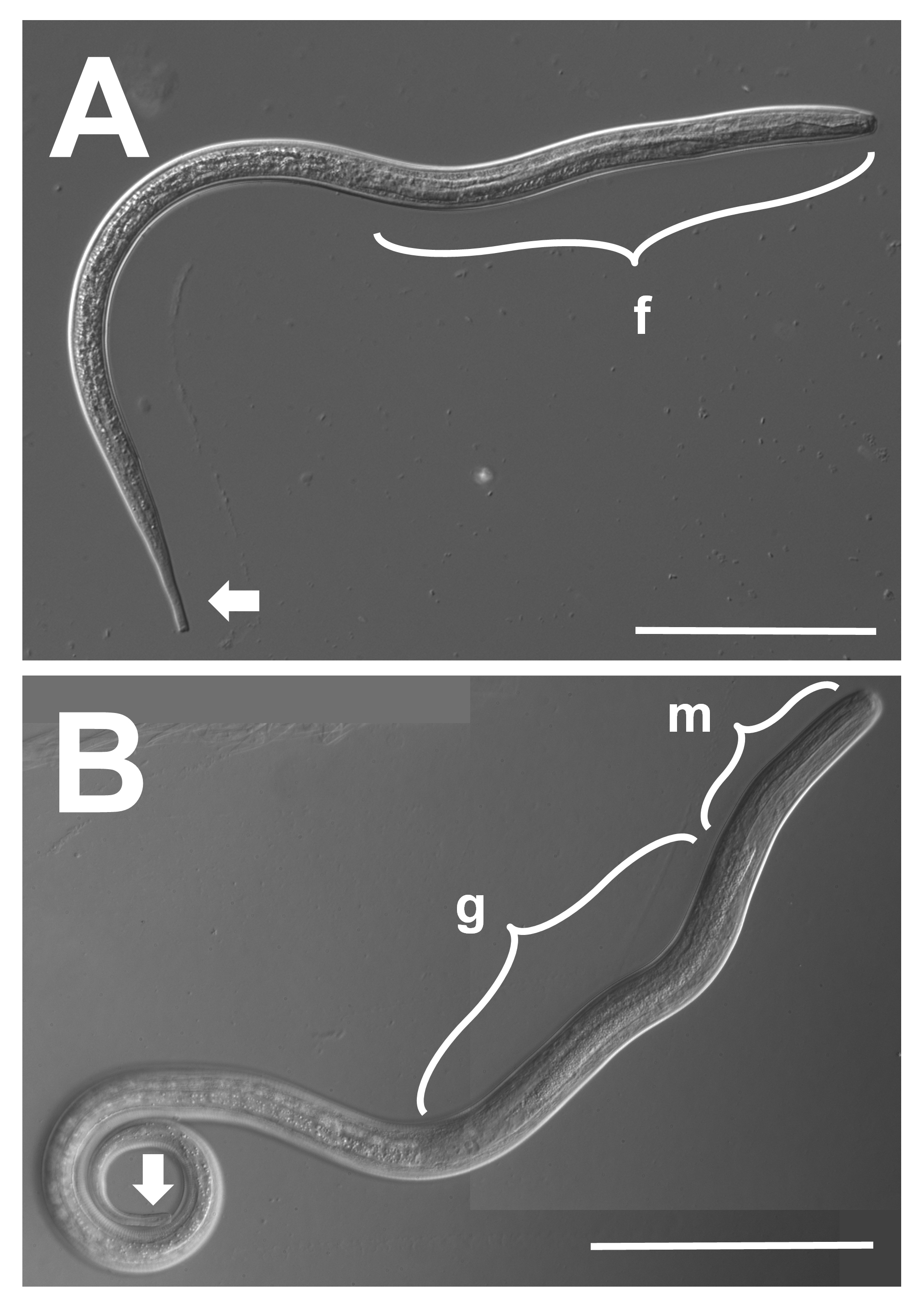

Supplement: Figure S1 — Morphological comparison of S. stercoralis L3i and L3+. S. stercoralis third-stage larvae were photographed as (A) developmentally arrested and non-feeding third-stage larvae (L3i) or (B) as feeding third-stage larvae activated in vivo for three days in a permissive host (L3+). Overall, the body of the (A) L3i is more radially constricted than that of the (B) L3+. Similarly, the (A) L3i has a filariform pharynx (f) that is radially constricted forming a long thin tube extending almost to the midpoint of the worm, while the (B) L3+ has a pharynx that is pumping, is not radially constricted, and has differentiated into both muscular (m) and glandular(g) segments. The fork-shaped tail (arrow) in both the (A) L3i and (B) L3+ is characteristic of third-stage larvae in Strongyloides species and is lost in subsequent larval stages. Scale bar represents 100 µM. (TIF) [file pntd.0001854.s001.tif]

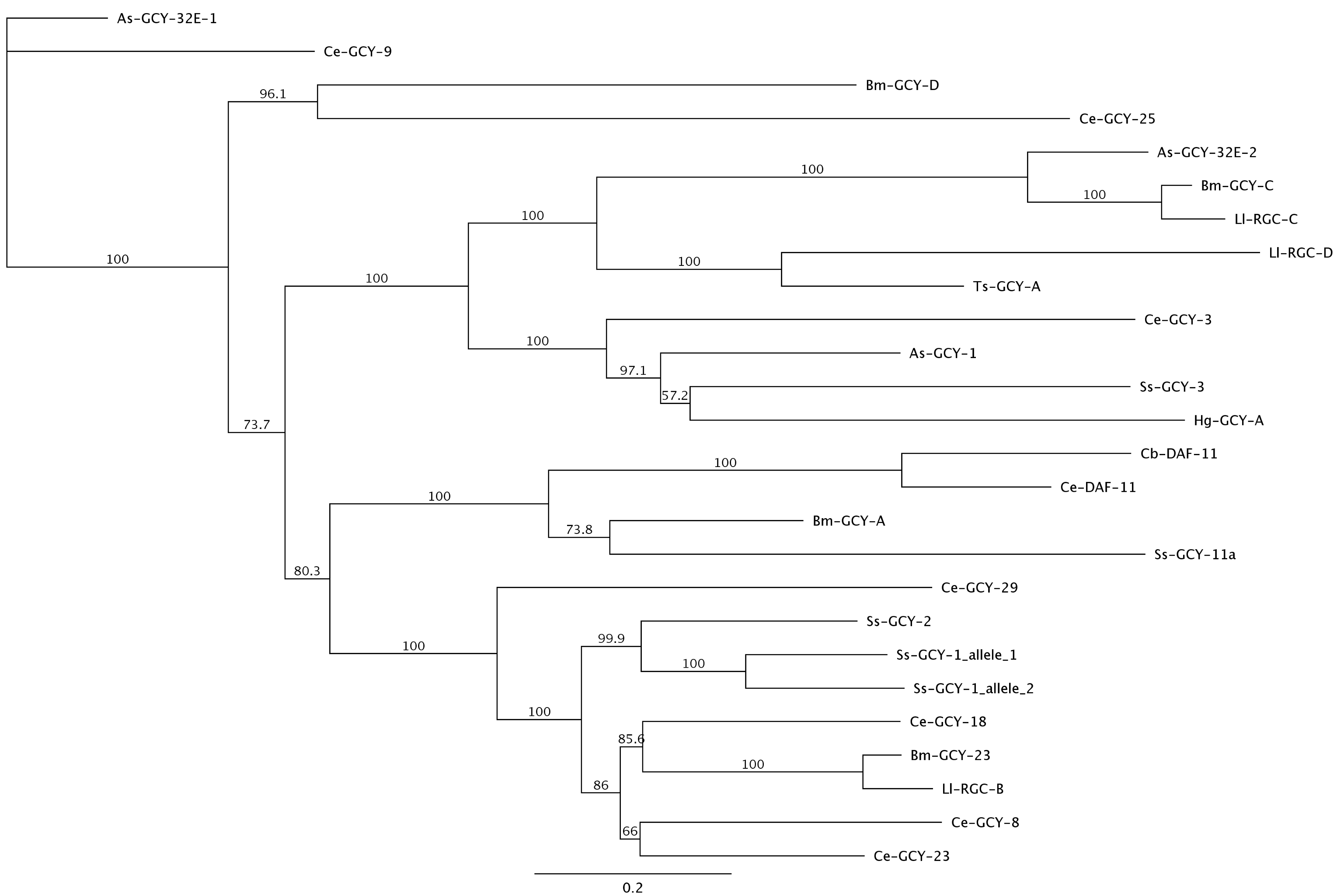

Supplement: Figure S2 — Phylogenetic analysis of phylum Nematoda guanylyl cyclases similar to Ce -DAF-11. A protein alignment, generated with Clustal W, of several predicted guanylyl cyclases similar to Ce-DAF-11 from S. stercoralis and other parasitic nematodes was used to construct a neighbor-joining tree with 100 iterations of boot-strapping. The predicted Ss-GCY-11 protein grouped with Ce-DAF-11. Abbreviations: Ascaris suum (As), Brugia malayi (Bm), Caenorhabditis briggsae (Cb), Caenorhabditis elegans (Ce), Heterodera glycines (Hg), Loa loa (Ll), Strongyloides stercoralis (Ss), and Trichinella spiralis (Ts). The scale bar represents substitutions per position. Accession numbers are listed in Data S4. (TIF) [file pntd.0001854.s002.tif]

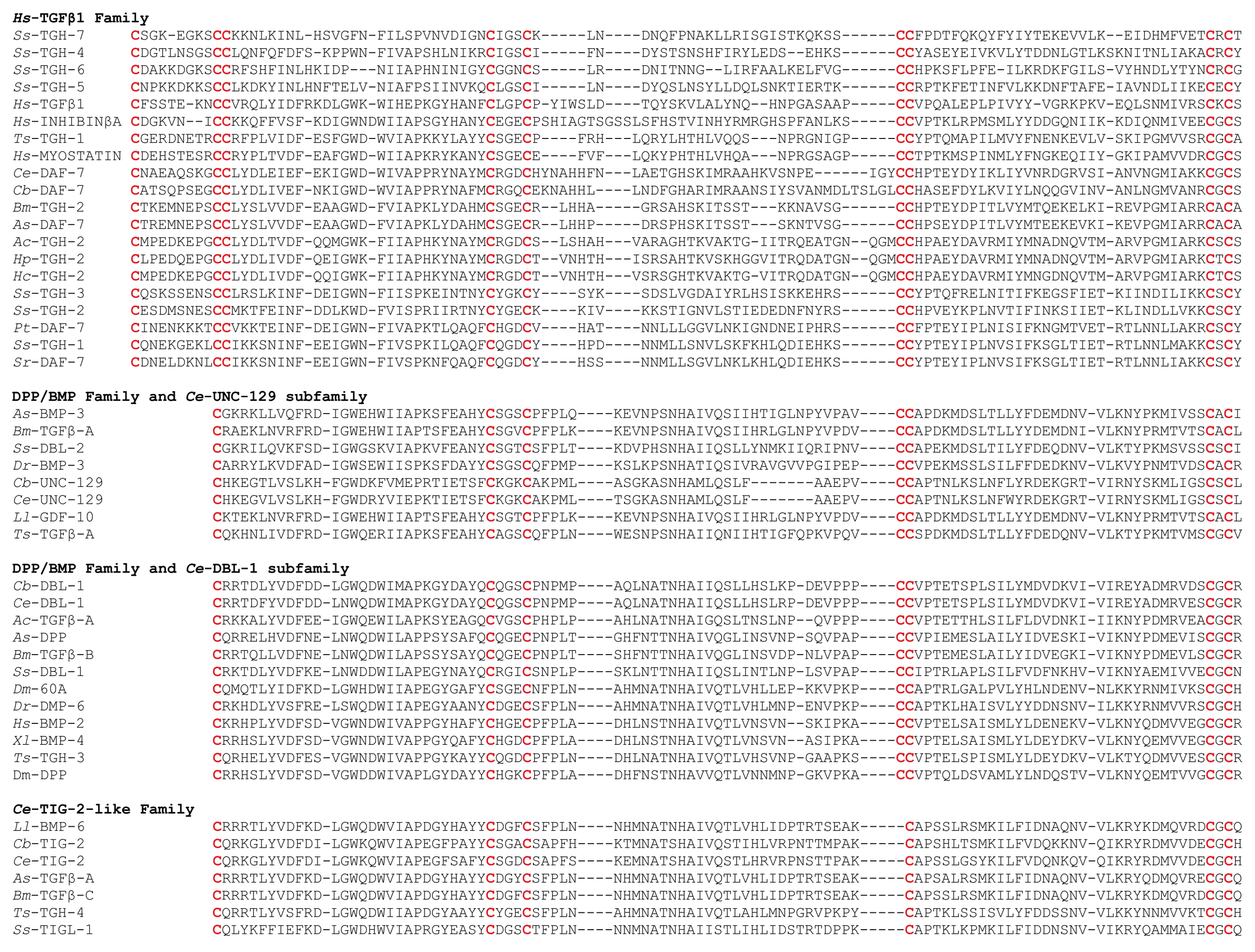

Supplement: Figure S3 — Protein alignment of TGFβ ligand domains by cysteine architecture. Protein sequence alignment of TGFβ ligand domains including all cysteine residues in S. stercoralis and other metazoans revealed three distinct sets of cysteine architecture: Hs-TGFβ1-like ligands, including Ce-DAF-7, which have nine conserved cysteine residues; Drosophila melanogaster decapentaplegic (DPP) and vertebrate bone morphogenetic protein (BMP) -like ligands, including Ce-DBL-1 and Ce-UNC-129 subfamilies, which have seven conserved cysteine residues; and Ce-TIG-2-like ligands, which have six conserved cysteine residues. The conserved cysteine residues, critical for disulfide bond formation and “cysteine knot” folding, are in red. Abbreviations: Ancylostoma caninum (Ac), Ascaris suum (As), Brugia malayi (Bm), Caenorhabditis briggsae (Cb), Caenorhabditis elegans (Ce), Danio rerio (Dr), Drosophila melanogaster (Dm), Haemonchus contortus (Hc), Heligmosomoides polygyrus (Hp), Homo sapiens (Hs), Loa loa (Ll), Parastrongyloides trichosuri (Pt), Strongyloides ratti (Sr), Strongyloides stercoralis (Ss), Trichinella spiralis (Ts), and Xenopus laevis (Xl). Accession numbers are listed in Data S4. (TIF) [file pntd.0001854.s003.tif]

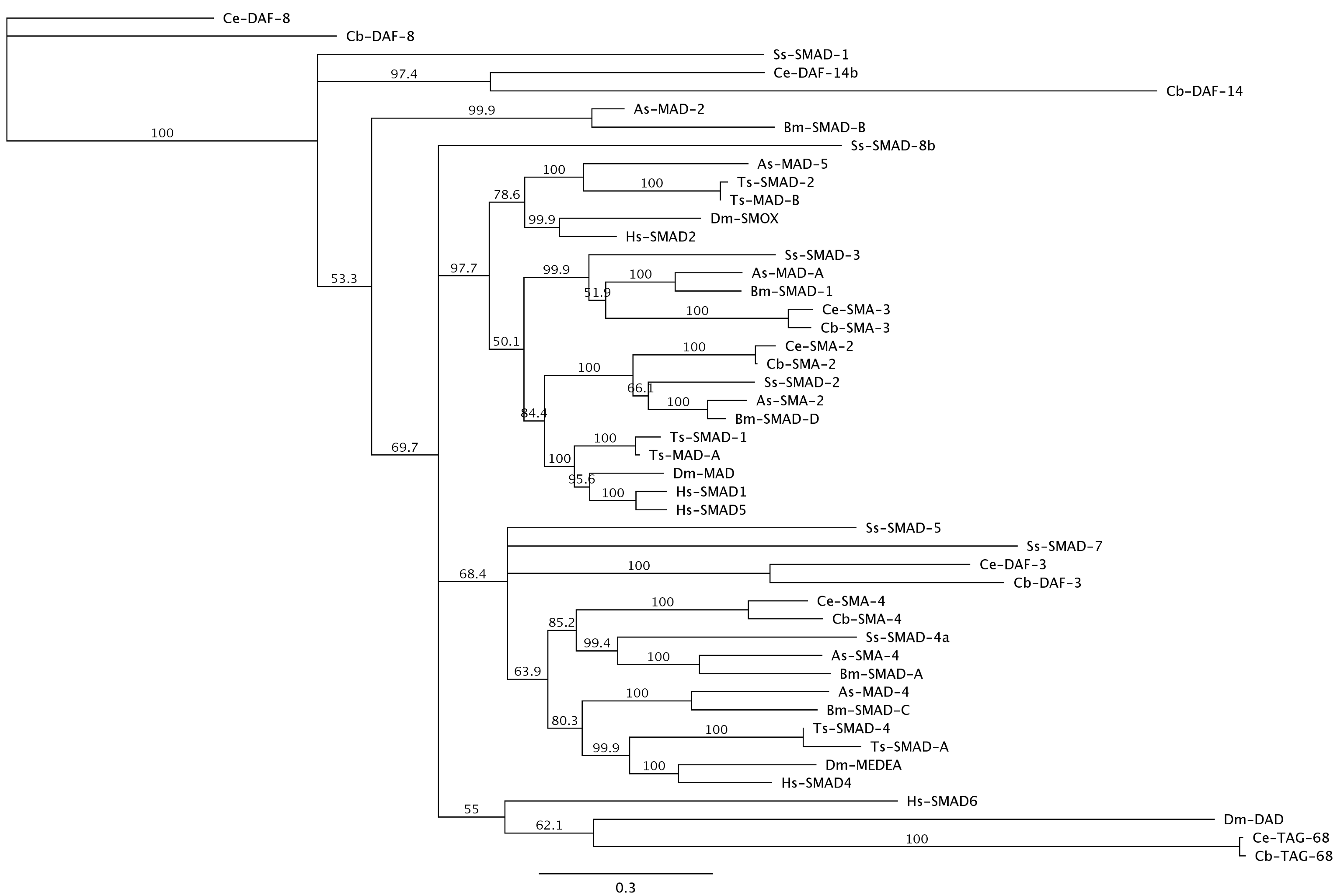

Supplement: Figure S4 — Phylogenetic analysis of phylum Nematoda SMAD homologs. A protein alignment, generated with Clustal W, of all publicly available phylum Nematoda SMAD homolog predicted proteins, was used to construct a neighbor-joining tree with 100 iterations of boot-strapping. The small body size and male tail abnormal (Sma/Mab) TGFβ pathway SMADs, including Ce-SMA-2, Ce-SMA-3, and Ce-SMA-4, resolved into distinct clades while the dauer TGFβ pathway SMADs, including Ce-DAF-3, Ce-DAF-8, and Ce-DAF-14, did not. Abbreviations: Ascaris suum (As), Brugia malayi (Bm), Caenorhabditis briggsae (Cb), Caenorhabditis elegans (Ce), Drosophila melanogaster (Dm), Heterodera glycines (Hg), Homo sapiens (Hs), Loa loa (Ll), Strongyloides stercoralis (Ss), and Trichinella spiralis (Ts). The scale bar represents substitutions per position. Accession numbers are listed in Data S4. (TIF) [file pntd.0001854.s004.tif]

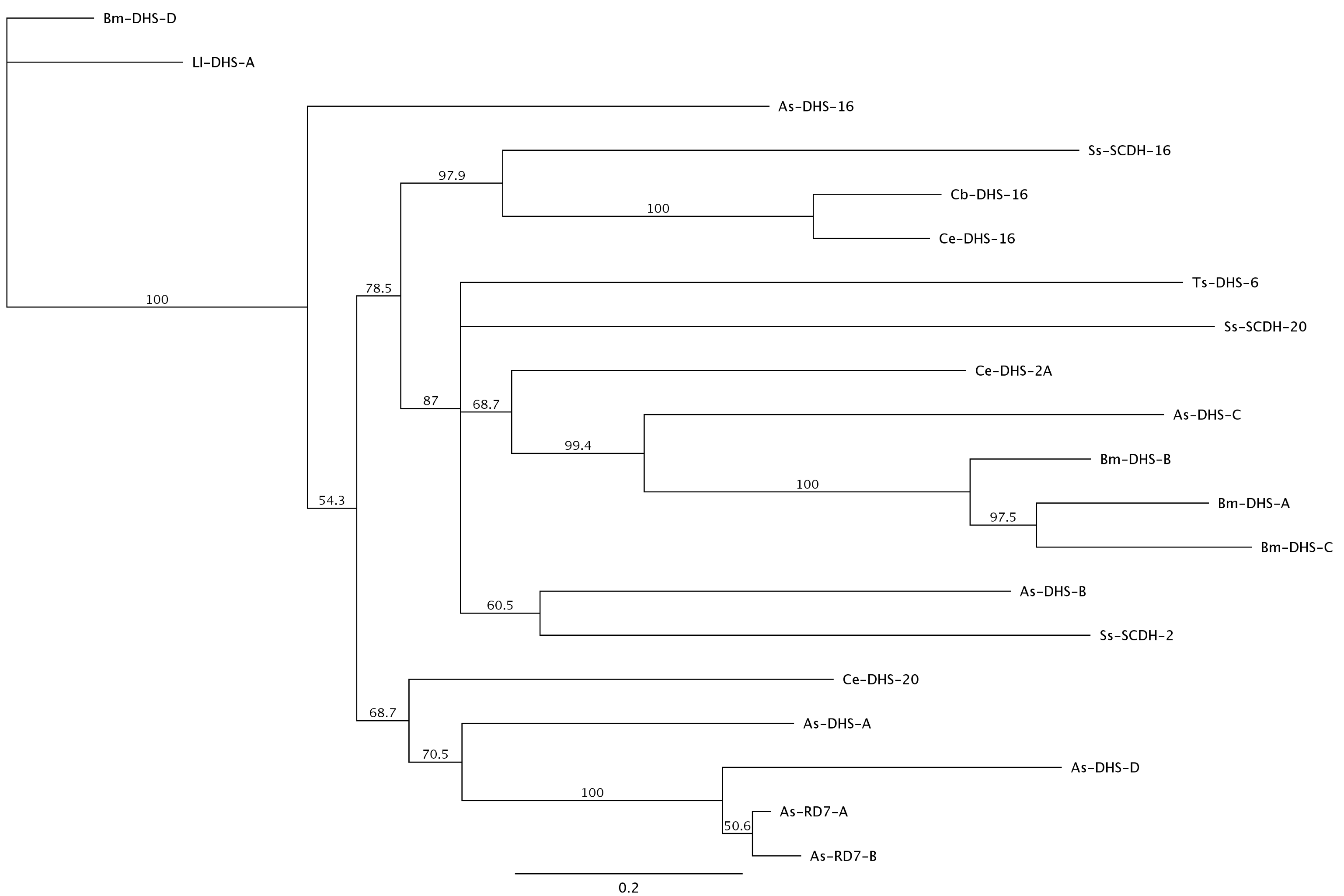

Supplement: Figure S5 — Phylogenetic analysis of phylum Nematoda short-chain dehydrogenase homologs similar to Ce -DHS-16. A protein alignment, generated with Clustal W, of phylum Nematoda short-chain dehydrogenase homologs similar to Ce-DHS-16 was used to construct a neighbor-joining tree with 100 iterations of boot-strapping. The predicted protein for Ss-SCDH-16 grouped closest to Ce-DHS-16. Abbreviations: Ascaris suum (As), Brugia malayi (Bm), Caenorhabditis briggsae (Cb), Caenorhabditis elegans (Ce), Loa loa (Ll), Strongyloides stercoralis (Ss), and Trichinella spiralis (Ts). The scale bar represents substitutions per position. Accession numbers are listed in Data S4. (TIF) [file pntd.0001854.s005.tif]

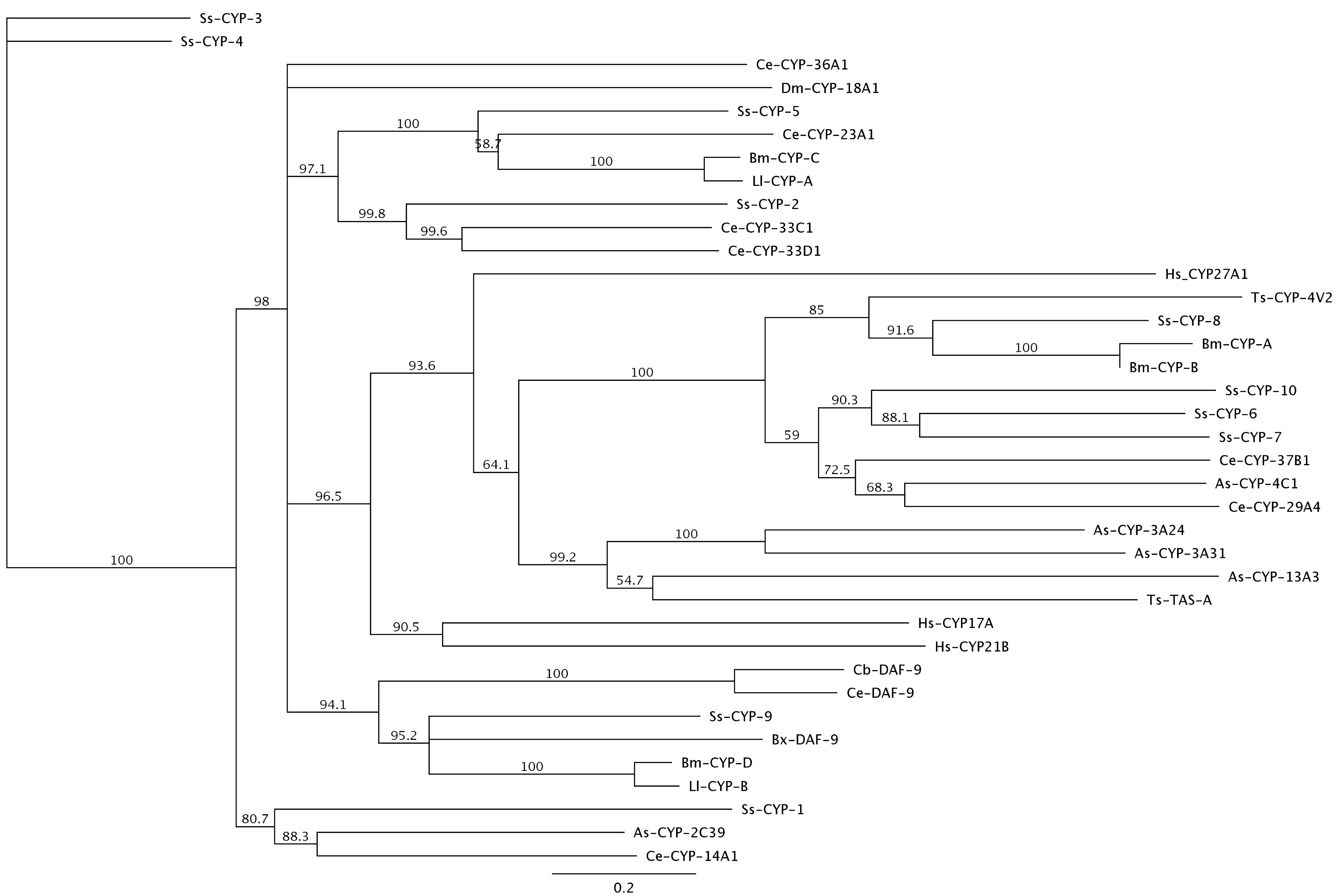

Supplement: Figure S6 — Phylogenetic analysis of phylum Nematoda cytochrome P450 homologs similar to Ce -DAF-9. A protein alignment, generated with Clustal W, of phylum Nematoda cytochrome P450 homologs similar to Ce-DAF-9 was used to construct a neighbor-joining tree with 100 iterations of boot-strapping. The predicted protein for Ss-CYP-9 grouped closest to Ce-DAF-9. Abbreviations: Ascaris suum (As), Brugia malayi (Bm), Bursaphelenchus xylophilus (Bx), Caenorhabditis briggsae (Cb), Caenorhabditis elegans (Ce), Drosophila melanogaster (Dm), Homo sapiens (Hs), Loa loa (Ll), Strongyloides stercoralis (Ss), and Trichinella spiralis (Ts). The scale bar represents substitutions per position. Accession numbers are listed in Data S4. (TIF) [file pntd.0001854.s006.tif]

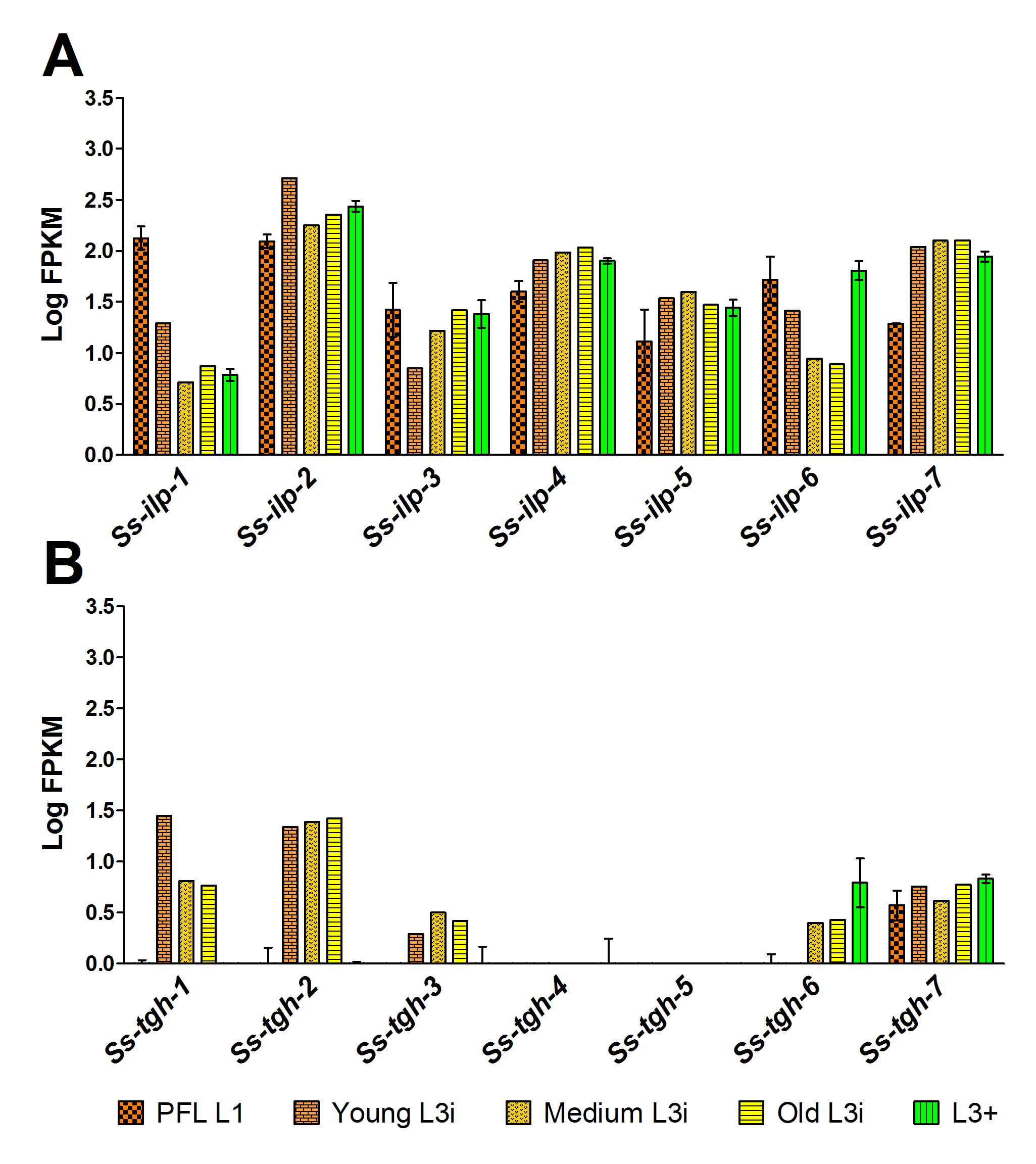

Supplement: Figure S7 — Regulation of ILP and TGFβ ligand genes during post-free-living development. Transcript abundances were determined for the coding region of (A–G) seven S. stercoralis insulin-like peptide (ILP) -encoding genes (Ss-ilp-1 through -7) and (H–N) seven S. stercoralis DAF-7-like TGFβ ligand encoding genes (Ss-tgh-1 through -7) over the course of post-free living larval development. Transcript abundances were examined for post-free-living first-stage larvae (PFL L1), infectious third-stage larvae (L3i), and in vivo activated third-stage larvae (L3+). The mean of both PFL L1 and L3+, from the three biological replicates, was plotted along with the 95% confidence intervals. Each biological sample of L3i was plotted individually according to relative age: L3i incubated at 21°C for 8 days (young L3i), L3i incubated at 25°C for 7 days (medium L3i), and L3i incubated at 21°C for 10 days (old L3i). Transcript abundances were calculated as fragments per kilobase of coding exon per million mapped reads (FPKM) and log transformed. The y-axes were scaled from 0 to 3.5 to aid comparison between genes. (TIF) [file pntd.0001854.s007.tif]

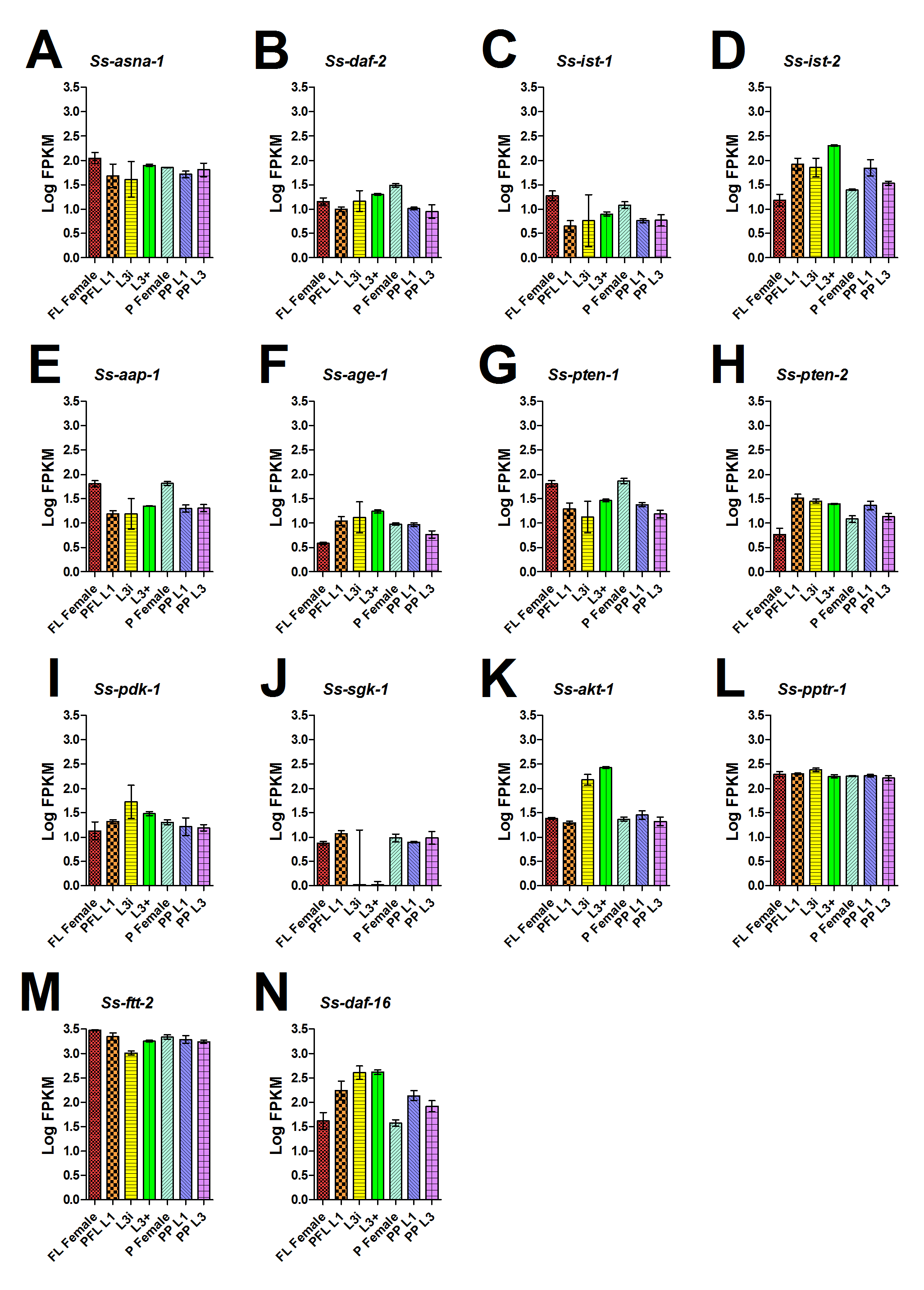

Supplement: Figure S8 — Developmental regulation of S. stercoralis homologs of intracellular IIS genes. Transcript abundances were determined for the coding region of S. stercoralis homologs of genes encoding insulin/IGF-1-like signaling (IIS) pathway proteins, including: (A) Ss-asna-1, a homolog of Ce-asna-1, which encodes a putative membrane transporter involved in insulin-like peptide secretion; (B) Ss-daf-2, a homolog of Ce-daf-2, which encodes an insulin-like receptor; (C) Ss-ist-1 and (D) Ss-ist-2, homologs of the insulin receptor substrate encoding gene Ce-ist-1; (E) Ss-aap-1, a homolog of Ce-aap-1, which encodes a phosphatidylinositol-3 (PI3) kinase accessory/regulatory subunit; (F) Ss-age-1, a homolog of Ce-age-1, which encodes a PI3 kinase catalytic subunit; (G) Ss-pten-1 and (H) Ss-pten-2, homologs of Ce-daf-18, which encodes a phosphatase opposing Ce-age-1 function; (I) Ss-pdk-1, a homolog of Ce-pdk-1, which encodes a PTEN-like kinase; (J) Ss-sgk-1, a homolog of the serum- and glucocorticoid-inducible kinase Ce-sgk-1; (K) Ss-akt-1, a homolog of Ce-akt-1 and -2, which encode AKT kinases; (L) Ss-pptr-1, a homolog of Ce-pptr-1, which encodes a B56 regulatory subunit of the PP2A phosphatase opposing Ce-AKT-1 function; (M) Ss-ftt-2, a homolog of the 14-3-3 encoding gene Ce-ftt-2; and (N) Ss-daf-16, a homolog of Ce-daf-16, which encodes a forkhead transcription factor. Transcript abundances were quantified in seven developmental stages: free-living females (FL Female), post-free-living first-stage larvae (PFL L1), infectious third-stage larvae (L3i), in vivo activated third-stage larvae (L3+), parasitic females (P Female), post-parasitic first-stage larvae (PP L1), and post-parasitic third-stage larvae (PP L3). Transcript abundances were calculated as fragments per kilobase of coding exon per million mapped reads (FPKM) and log transformed. Error bars represent 95% confidence intervals. The y-axes were scaled from 0 to 3.5 to aid comparison between genes. (TIF) [file pntd.0001854.s008.tif]

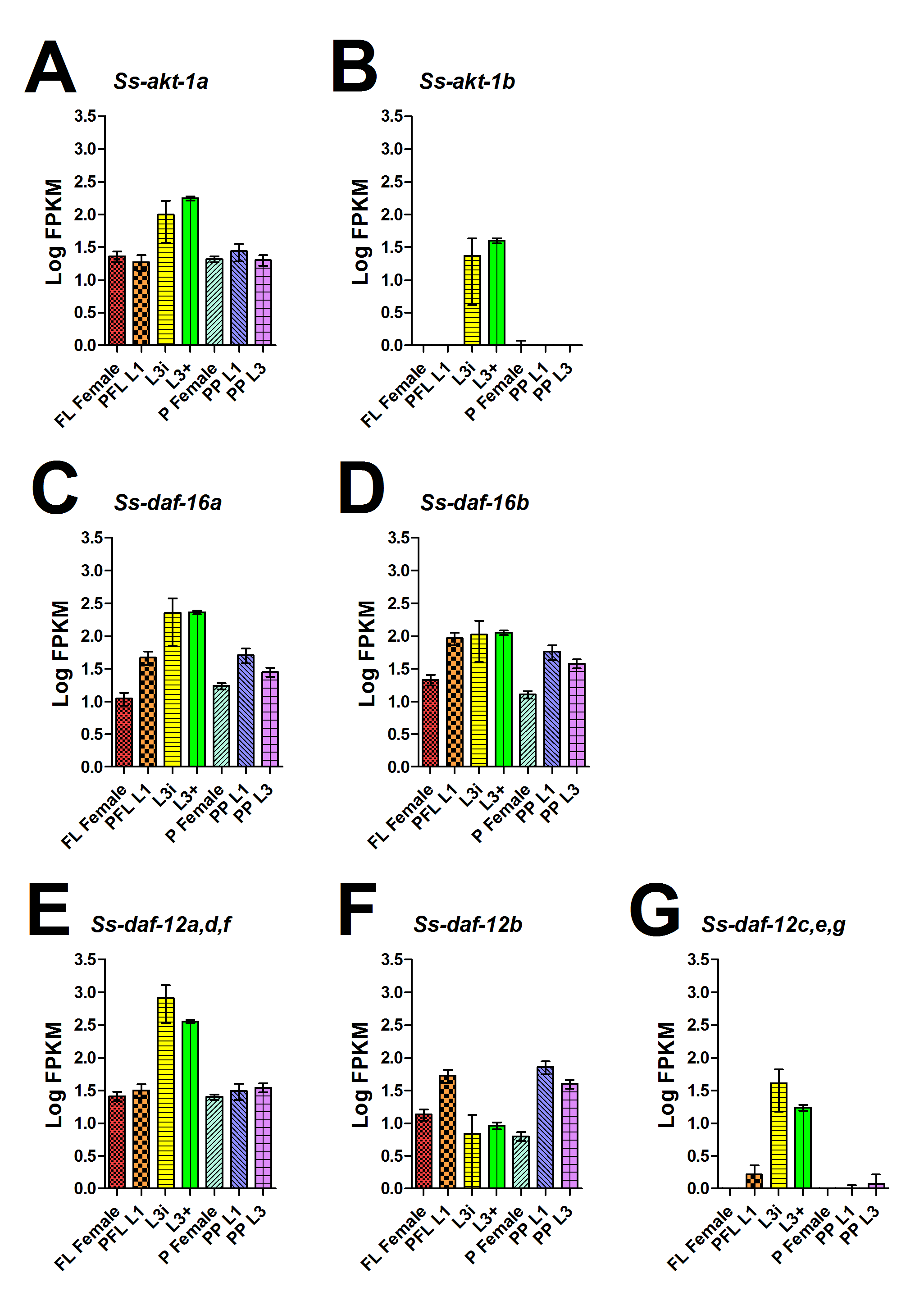

Supplement: Figure S9 — Developmental stage-specific transcripts of S. stercoralis akt-1 , daf-16 , and daf-12 isoforms. (A, B) Two transcripts, each from different promoters, are generated from the Ss-akt-1 locus: (A) Ss-akt-1a and (B) Ss-akt-1b. The Ss-akt-1b transcript encodes a putative 525 amino acid Ss-AKT-1B peptide that has 33 amino acids truncated from the conserved AKT pleckstrin homology (PH) domain at the N-terminus. The Ss-akt-1a transcript encodes a putative 580 amino acid Ss-AKT-1A protein with a conserved full-length PH domain. (C, D) The locus from which the S. stercoralis forkhead transcription factor Ss-daf-16 is transcribed also produces two transcripts, (C) Ss-daf-16a and (D) Ss-daf-16b, each from a different promoter. These transcripts encode a 741 amino acid Ss-DAF-16A predicted protein and a 566 amino acid Ss-DAF-16B predicted protein, which differ at the N-terminus. (E–G) The genomic locus encoding the S. stercoralis nuclear hormone receptor homolog Ss-DAF-12 expresses at least seven different transcripts, which we termed Ss-daf-12a-g, from several promoters. These seven transcripts encode a total of three predicted proteins, each differing at the N-terminus before the DNA-binding domain, with transcripts (E) Ss-daf-12a,d,f encoding a putative 752 amino acid Ss-DAF-12A protein, (F) Ss-daf-12b encoding a putative 947 amino acid Ss-DAF-12B protein, and (G) Ss-daf-12c,e,g encoding a putative 722 amino acid Ss-DAF-12C protein. Transcript abundances were quantified in seven developmental stages: free-living females (FL Female), post-free-living first-stage larvae (PFL L1), infectious third-stage larvae (L3i), in vivo activated third-stage larvae (L3+), parasitic females (P Female), post-parasitic first-stage larvae (PP L1), and post-parasitic third-stage larvae (PP L3). Transcript abundances were calculated as fragments per kilobase of transcript exon per million mapped reads (FPKM) and log transformed. Error bars represent 95% confidence intervals. The y-axes were scal [file pntd.0001854.s009.tif]

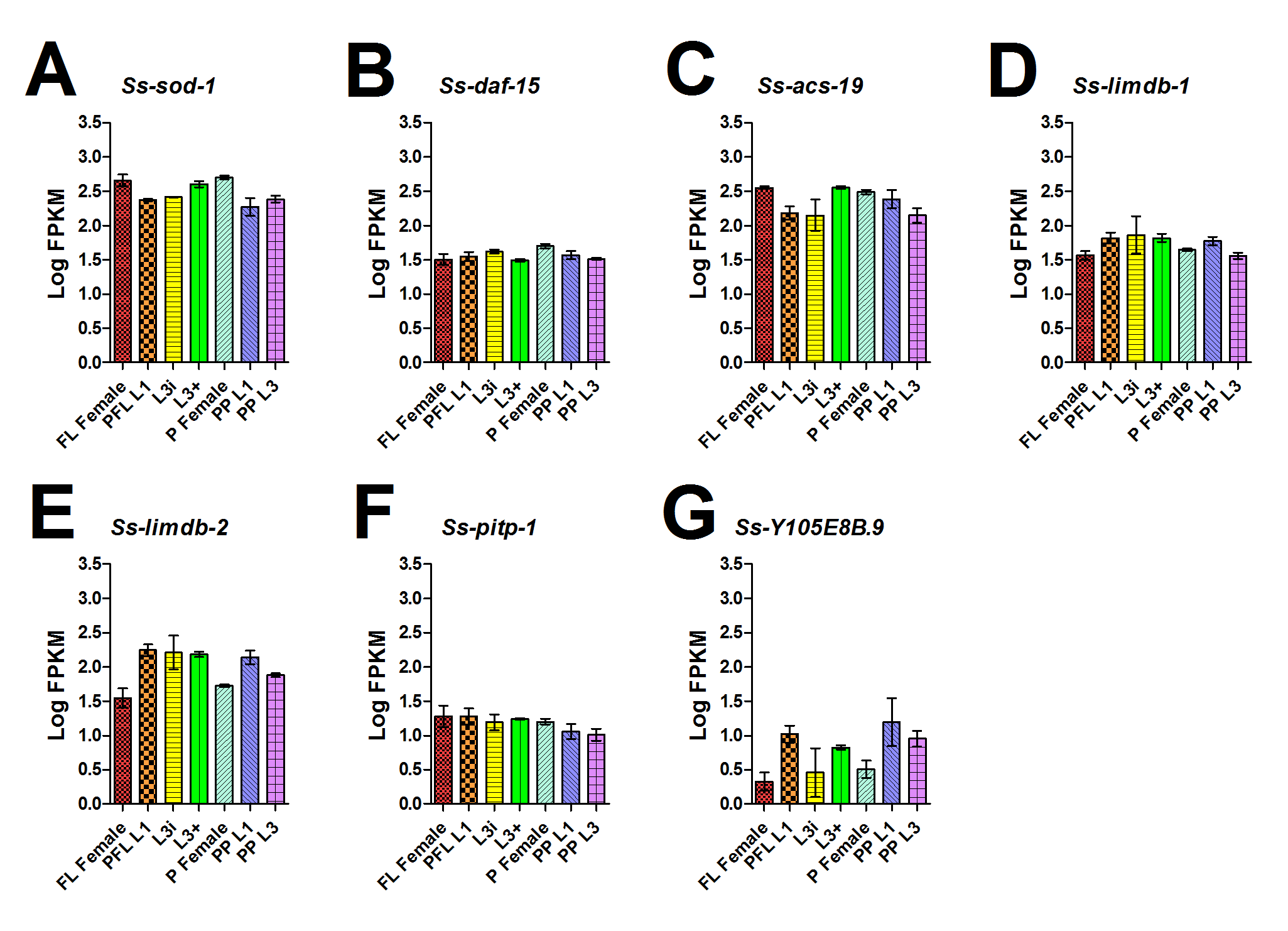

Supplement: Figure S10 — Lack of developmental regulation of S. stercoralis homologs of Ce -DAF-16-regulated genes. Transcript abundances were examined for S. stercoralis homologs of C. elegans genes that are transcriptionally regulated directly by Ce-DAF-16 and have a phenotype associated with RNAi knock-down. (A) Ss-sod-1 is the sole S. stercoralis superoxide dismutase and is a homolog of Ce-sod-3. (B) Ss-daf-15 and (C) Ss-acs-19 are homologs of Ce-daf-15 and Ce-acs-19, respectively. Both (D) Ss-limdb-1 and (E) Ss-limdb-2 are homologs of Ce-lim-1, while (F) Ss-pitp-1 and (G) Ss-Y105E8B.9 are homologs of Ce-pitp-1 and Ce-Y105E8B.9, respectively. Transcript abundances were quantified in seven developmental stages: free-living females (FL Female), post-free-living first-stage larvae (PFL L1), infectious third-stage larvae (L3i), in vivo activated third-stage larvae (L3+), parasitic females (P Female), post-parasitic first-stage larvae (PP L1), and post-parasitic third-stage larvae (PP L3). Transcript abundances were calculated as fragments per kilobase of coding exon per million mapped reads (FPKM) and log transformed. Error bars represent 95% confidence intervals. The y-axes were scaled from 0 to 3.5 to aid comparison between genes. (TIF) [file pntd.0001854.s010.tif]

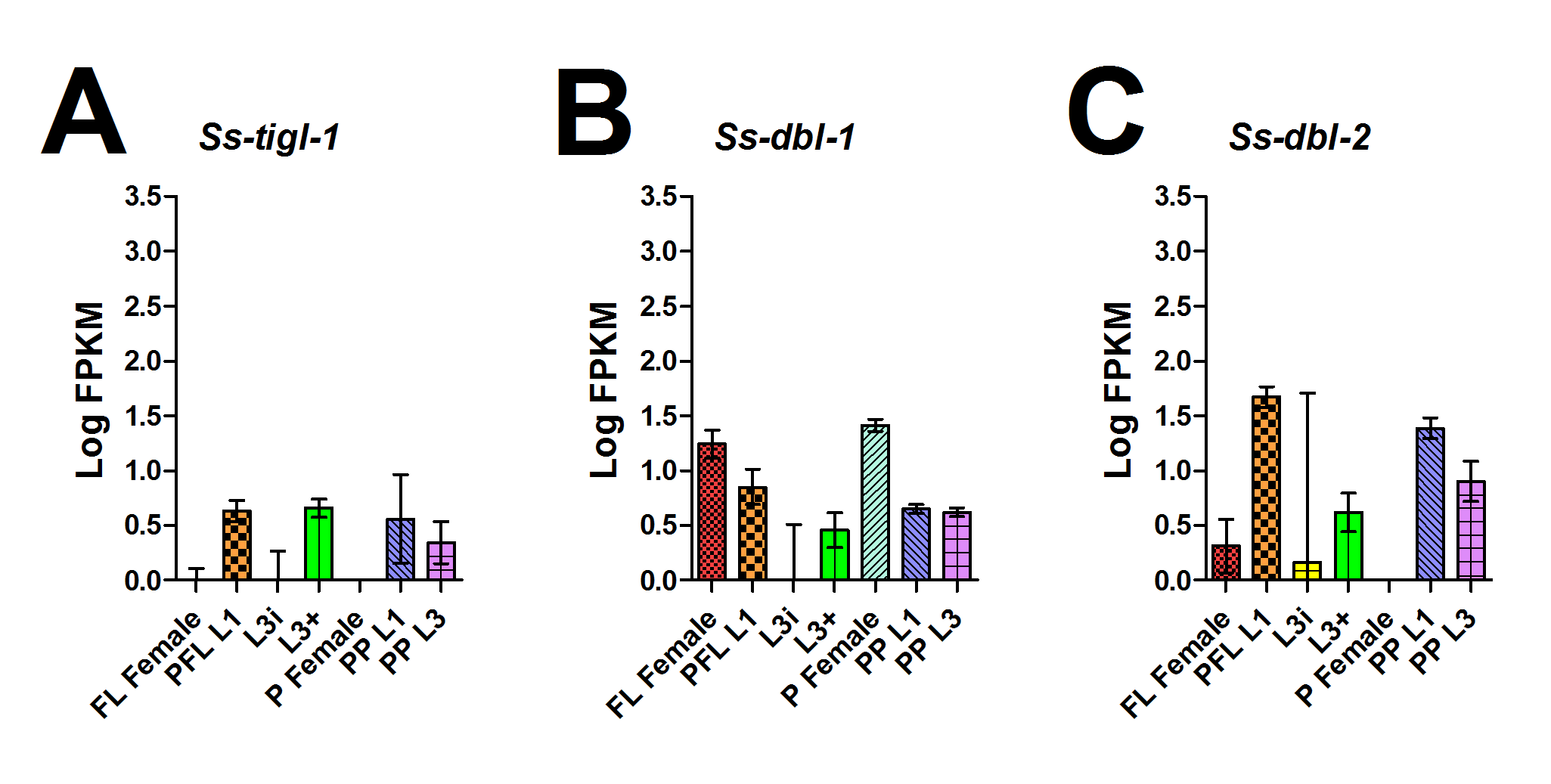

Supplement: Figure S11 — Developmental regulation of additional S. stercoralis TGFβ ligand transcripts. The S. stercoralis genome contains three TGFβ super-family ligands that do not group with the human TGFβ1 and Ce-DAF-7 family: (A) Ss-tigl-1, a gene encoding a homolog of Ce-TIG-2, a TGFβ ligand that does not have any known biological function or receptor; (B) Ss-dbl-1, a gene encoding a homolog of Ce-DBL-1, a TGFβ ligand homolog of D. melanogaster decapentaplegic (DPP) and vertebrate bone morphogenetic protein (BMP), which signals through the small body size and male tail abnormal (Sma/Mab) pathway; and (C) Ss-dbl-2, a gene encoding a homolog of the Ce-UNC-129 TGFβ ligand that regulates cell migration and axon guidance and acts independently of type I or type II receptors. Transcript abundances were quantified in seven developmental stages: free-living females (FL Female), post-free-living first-stage larvae (PFL L1), infectious third-stage larvae (L3i), in vivo activated third-stage larvae (L3+), parasitic females (P Female), post-parasitic first-stage larvae (PP L1), and post-parasitic third-stage larvae (PP L3). Transcript abundances were calculated as fragments per kilobase of coding exon per million mapped reads (FPKM) and log transformed. Error bars represent 95% confidence intervals. The y-axes were scaled from 0 to 3.5 to aid comparison between genes. (TIF) [file pntd.0001854.s011.tif]

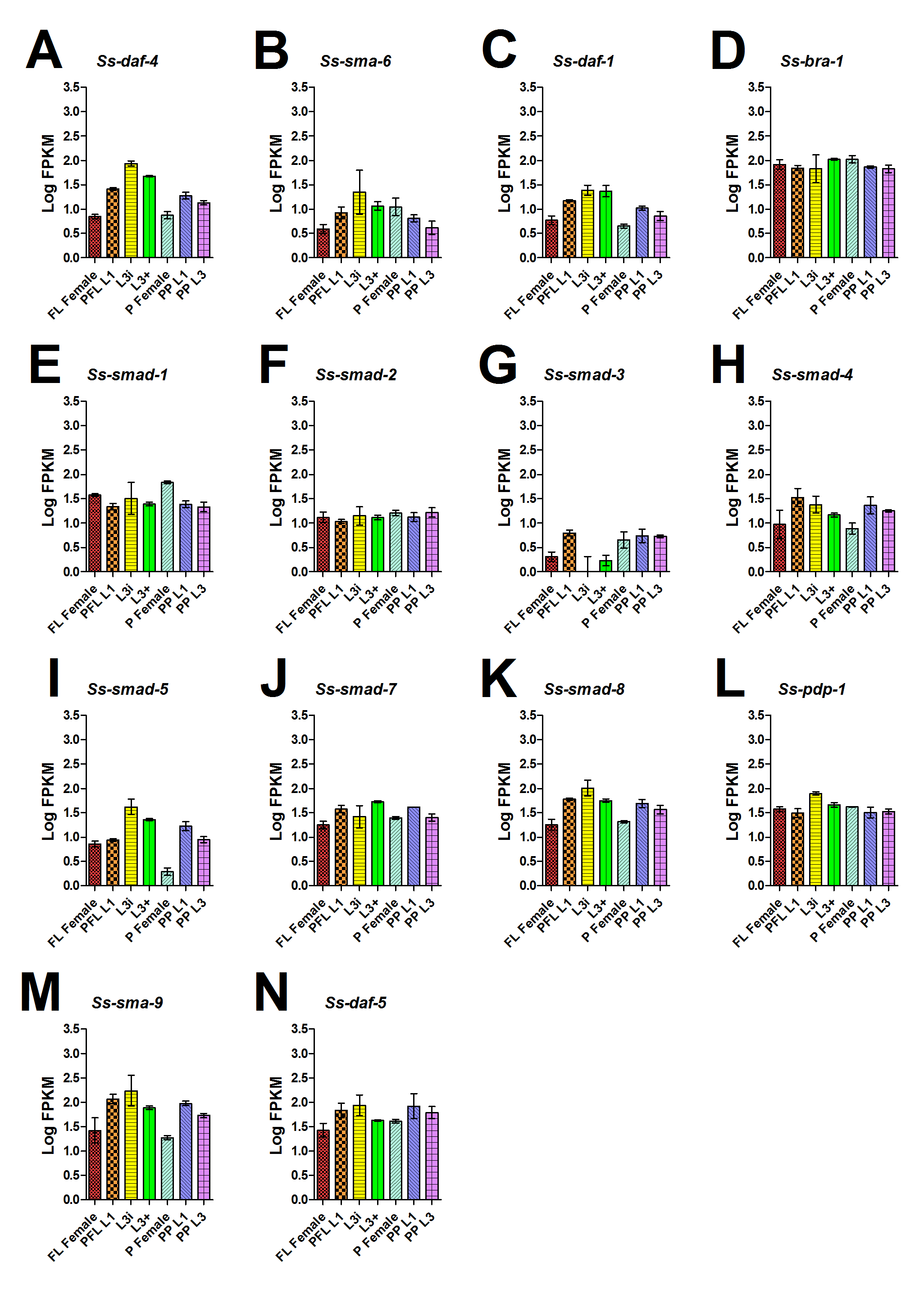

Supplement: Figure S12 — Developmental regulation of TGFβ signaling homologs in S. stercoralis . Developmental expression patterns were assessed for members of both the dauer and small body size and male tail abnormal (Sma/Mab) TGFβ pathways: (A) Ss-daf-4, a gene encoding a homolog of the type II receptor Ce-DAF-4; (B) Ss-sma-6, a gene encoding a homolog of the Sma/Mab type I receptor Ce-SMA-6; (C) Ss-daf-1, a gene encoding a homolog of the dauer type I receptor Ce-DAF-1; (D) Ss-bra-1, a gene encoding a homolog of the Ce-DAF-1 negative regulator Ce-BRA-1; (E) Ss-smad-1, a gene encoding a SMAD similar to Ce-DAF-14; (F) Ss-smad-2, (G) Ss-smad-3, and (H) Ss-smad-4, genes encoding SMAD homologs similar to Ce-SMA-2, Ce-SMA-3, and Ce-SMA-4, respectively; (I) Ss-smad-5, (J) Ss-smad-7, and (K) Ss-smad-8, genes encoding SMAD homologs similar to Ce-DAF-3 and Ce-DAF-8; (L) Ss-pdp-1, a gene encoding a homolog of the phosphatase Ce-PDP-1; (M) Ss-sma-9, a gene encoding a homolog of the Sma/Mab transcriptional co-factor Ce-SMA-9; and (N) Ss-daf-5, a gene encoding a Sno/Ski transcription factor homolog similar to Ce-DAF-5. Transcript abundances were quantified in seven developmental stages: free-living females (FL Female), post-free-living first-stage larvae (PFL L1), infectious third-stage larvae (L3i), in vivo activated third-stage larvae (L3+), parasitic females (P Female), post-parasitic first-stage larvae (PP L1), and post-parasitic third-stage larvae (PP L3). Transcript abundances were calculated as fragments per kilobase of coding exon per million mapped reads (FPKM) and log transformed. Error bars represent 95% confidence intervals. The y-axes were scaled from 0 to 3.5 to aid comparison between genes. (TIF) [file pntd.0001854.s012.tif]

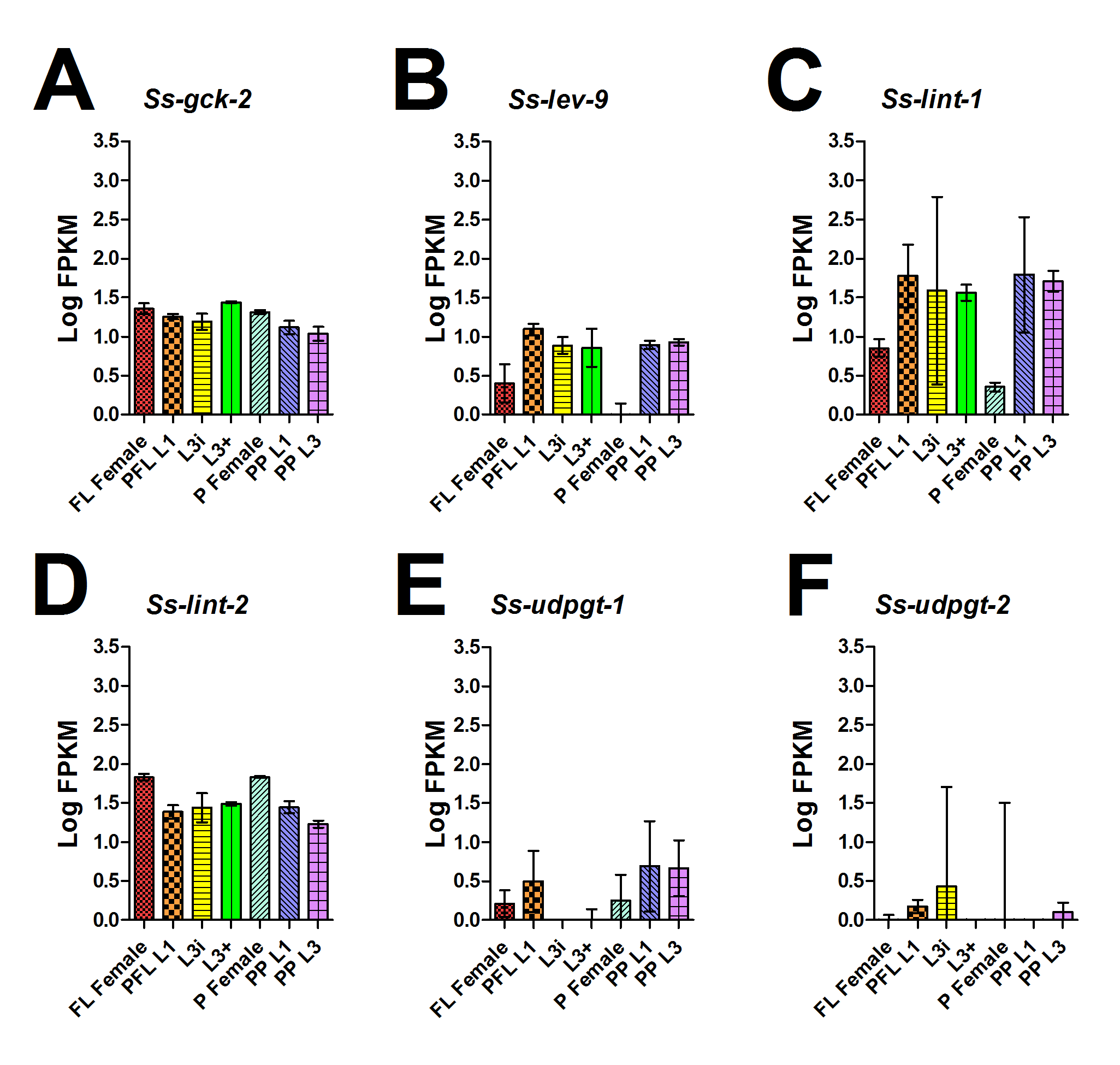

Supplement: Figure S13 — Developmental regulation of S. stercoralis homologs of Ce -DAF-12-regulated genes. Transcript abundances of S. stercoralis homologs of C. elegans genes transcriptionally regulated directly by Ce-DAF-12 during dauer development were examined: (A) Ss-gck-2, a gene encoding a homolog of Ce-GCK-2; (B) Ss-lev-9, a gene encoding a homolog of Ce-LEV-9; (C) Ss-lint-1 and (D) Ss-lint-2, genes encoding homologs of Ce-LIT-1; and (E) Ss-udpgt-1 and (F) Ss-udpgt-2, genes encoding homologs of Ce-UGT-65. Transcript abundances were quantified in seven developmental stages: free-living females (FL Female), post-free-living first-stage larvae (PFL L1), infectious third-stage larvae (L3i), in vivo activated third-stage larvae (L3+), parasitic females (P Female), post-parasitic first-stage larvae (PP L1), and post-parasitic third-stage larvae (PP L3). Transcript abundances were calculated as fragments per kilobase of coding exon per million mapped reads (FPKM) and log transformed. Error bars represent 95% confidence intervals. The y-axes were scaled from 0 to 3.5 to aid comparison between genes. (TIF) [file pntd.0001854.s013.tif]
