# Supplementary material for: RNAseq Analysis of the Parasitic Nematode Strongyloides stercoralis Reveals Divergent Regulation of Canonical Dauer Pathways
Source: PLoS Negl Trop Dis. 2012 Oct 25;6(10):e1854. doi: 10.1371/journal.pntd.0001854 (PMC3493385; doi:10.1371/journal.pntd.0001854)
Supplement: Text S1 — Supplemental methods for isolation of Strongyloides stercoralis developmental stages. (DOC) [file pntd.0001854.s025.doc]

**Text S1. Supplemental methods for isolation of *Strongyloides stercoralis* developmental stages.**

**Post-parasitic first-stage larvae (PP L1)**

Post-parasitic L1 from the *S. stercoralis* PV001 line, which has >95% heterogonic development under standard culture conditions, were isolated. Larvae were migrated from fresh feces using the Baermann technique, with a 28-29°C water temperature for two hours. Four 50 ml aliquots were drained and centrifuged at room temperature for 10 minutes; subsequently, the supernatants were removed. Pelleted debris were combined, resuspended in 1-2 ml water, and mixed with an equal volume of approximately 30°C liquid, 1% low melting temperature agarose (Lonza, Basel, Switzerland). The suspension was transferred to a petri dish and allowed to solidify, first on 4°C glass until the agarose began to gel and then at room temperature for 10-15 minutes. Subsequently, 14 ml of BU buffer was added, and the dish was placed at 25-29°C for two hours, allowing larvae to migrate into the liquid. Any precociously developing L3i were removed from the liquid, which was then transferred to a 15 ml tube and centrifuged at room temperature for 10 minutes; subsequently, the supernatant was removed. The larval pellet was transferred to a 1.5 ml tube and centrifuged again; subsequently, the supernatant was removed. Larvae were mixed with 200 μl of TRIzol reagent and snap frozen in liquid nitrogen.

**Post-parasitic third-stage larvae (PP L3) heterogonically developing to free-living adults**

*S. stercoralis* PV001 line larvae were migrated from charcoal coprocultures incubated for 24 hours at 21°C, using the Baermann technique, with a 28-29°C water temperature for one hour. One 40 ml aliquot was removed, and worms were allowed to settle at 1 *g* for 10 minutes. Larvae were resuspended in 1-2 ml water and mixed with an equal volume of approximately 30°C liquid, 1% low melting temperature agarose. The suspension was transferred to a petri dish and allowed to solidify, first on 4°C glass until the agarose began to gel and then at room temperature for 10-15 minutes. Subsequently, 14 ml of BU buffer was added, and the dish was placed at 25-29°C for one hour, allowing larvae to migrate into the liquid. Approximately third-stage larvae, identified by overall length and an elongated gonadal primordium (late L2 or L3) but without development of gonadal arms (L4), were removed to a 1.5 ml tube with a pipette. Larvae with a visible spicule, developing to adult males, were not selected, thus enriching the population for larvae developing to free-living females. Larvae were allowed to settle at 1 *g* for 10 minutes; subsequently, the supernatant was removed. Larvae were mixed with 200 μl of TRIzol reagent and snap frozen in liquid nitrogen.

**Free-living females (FL Females)**

*S. stercoralis* PV001 line adult free-living worms were migrated from charcoal coprocultures, incubated for 48 hours at 21°C, using the Baermann technique, with a 28-29°C water temperature for one hour. One 30 ml aliquot was removed, and worms were allowed to settle at 1 *g* for 10 minutes. Worms were resuspended in 1-2 ml water and mixed with an equal volume of approximately 30°C liquid, 1% low melting temperature agarose. The suspension was transferred to a petri dish and allowed to solidify, first on 4°C glass until the agarose began to gel and then at room temperature for 10-15 minutes. Subsequently, 14 ml of BU buffer was added, and the dish was placed at 28°C for 30 minutes, allowing adult worms to migrate into the liquid. Free-living females with 2-10 eggs per gonadal arm were removed to a 1.5 ml tube with a pipette and allowed to settle at 1 *g* for 10 minutes; subsequently, the supernatant was removed. Females were then mixed with 200 μl of TRIzol reagent and snap frozen in liquid nitrogen.

**Post-free-living first-stage larvae (PFL L1)**

*S. stercoralis* PV001 line larvae were migrated from charcoal coprocultures, incubated for three days at 21°C, using the Baermann technique, with a 29°C water temperature for two hours. Four 50 ml aliquots were drained and allowed to settle at 1 *g* for 6-8 minutes. Free-living adults were removed from the bottom of each tube. The remaining suspension of larvae was centrifuged at room temperature for 10 minutes, and the supernatants were removed. Pelleted larvae were resuspended in 1-2 ml water and mixed with an equal volume of approximately 30°C liquid, 1% low melting temperature agarose. The suspension was transferred to a petri dish and allowed to solidify, first on 4°C glass until the agarose began to gel and then at room temperature for 10-15 minutes. Subsequently, 14 ml of BU buffer was added, and the dish was placed at 29°C for two hours, allowing larvae to migrate into the liquid. All remaining free-living adults were removed with a pipette, leaving only post-free-living L1. The larval suspension was transferred to a 15 ml tube and centrifuged for 10 minutes; subsequently, the supernatant was removed. The larval pellet was transferred to a 1.5 ml tube and centrifuged again; the supernatant was removed. Larvae were mixed with 200 μl of TRIzol reagent and snap frozen in liquid nitrogen.

**Infectious third-stage larvae (L3i)**

*S. stercoralis* PV001 line larvae were migrated from charcoal coprocultures, incubated for 8-10 days at 21°C or 7 days at 25°C, using the Baermann technique, with a 27-30°C water temperature for one hour. One 40 ml aliquot was removed and centrifuged for three minutes; subsequently, the supernatant was removed. L3i were then washed twice in deionized water, and the supernatant was removed. L3i were resuspended in 1-2 ml water and mixed with an equal volume of approximately 30°C liquid, 1% low melting temperature agarose. The suspension was transferred to a petri dish and allowed to solidify, first on 4°C glass until the agarose began to gel and then at room temperature for 10-15 minutes. Subsequently, 14 ml of BU buffer was added, and the dish was placed at 28°C for one hour, allowing larvae to migrate into the liquid. The L3i suspension was transferred to a 15 ml tube and centrifuged for 10 minutes; subsequently, the supernatant was removed. L3i in 200 μl aliquots were divided among 1.5 ml tubes, and each aliquot of L3i was mixed with 200 μl of TRIzol reagent and snap frozen in liquid nitrogen.

**L3i activated *in vivo* (L3+)**

*S. stercoralis* PV001 line L3i for experimental infections were isolated from 10 day-old cultures incubated at 21°C by the Baermann technique, with a 27°C water temperature for 1.5 hours. One 30 ml aliquot was removed and allowed to settle at 1 *g* for three minutes; subsequently, the supernatant was removed. L3i were resuspended in 1-2 ml water and mixed with an equal volume of approximately 30°C liquid, 1% low melting temperature agarose. The suspension was transferred to a petri dish and allowed to solidify, first on 4°C glass until the agarose began to gel and then at room temperature for 10-15 minutes. Subsequently, 14 ml of BU buffer was added, and the dish was placed at 27°C for 30 minutes, allowing larvae to migrate into the liquid. The L3i suspension was transferred to a 15 ml tube and allowed to settle at 1 *g* for 15 minutes; subsequently, the supernatant was removed. L3i were then suspended in phosphate buffered saline (PBS) at a concentration of 4,000 L3i per 200 μl.

Mongolian gerbils were experimentally infected with 4,000 L3i and sacrificed three days after infection. The digestive organs were discarded to remove any worms which may have rapidly developed, and the remaining carcass was minced. Tissue-migrating third-stage larvae (L3+) were isolated using the Baermann technique, with L3+ migrated into DMEM supplemented with gentamycin for three hours at 37°C. Fresh DMEM + gentamycin was added, and L3+ were allowed to migrate for an additional two hours at 37°C. L3+ were then washed in DMEM + gentamycin at 37°C several times to remove gerbil tissue. Subsequently, L3+ from infections of single gerbils were then mixed with 200 μl of TRIzol reagent and snap frozen in liquid nitrogen.

**Parasitic females (P Females)**

*S. stercoralis* PV001 line L3i for experimental infections were isolated from seven day-old cultures incubated at 25°C by the Baermann technique, with a 28°C water temperature for one hour. One 40 ml aliquot was removed and centrifuged for five minutes at room temperature; subsequently, the supernatant was removed. L3i were then washed once more in de-ionized water, and the supernatant was removed. L3i were resuspended in 1-2 ml water and mixed with an equal volume of approximately 30°C liquid, 1% low melting temperature agarose. The suspension was transferred to a petri dish and allowed to solidify, first on 4°C glass until the agarose began to gel and then at room temperature for 10-15 minutes. Subsequently, 14 ml of BU buffer was added, and the dish was placed at 29°C for one hour, allowing larvae to migrate into the liquid. The L3i suspension was transferred to a 15 ml tube and centrifuged for five minutes; subsequently, the supernatant was removed. L3i were then suspended in phosphate buffered saline (PBS) at a concentration of 4,000 L3i per 200 μl.

Mongolian gerbils were experimentally infected with 4,000 L3i and sacrificed three weeks after infection. The gastrointestinal tract was removed and hung separately for each gerbil in graduated cylinders with DMEM, supplemented with gentamycin, for three hours at 37°C. Parasitic females were separated from the intestinal mucosa by repeatedly pipetting worms into fresh medium and removing debris. Cleaned worms were allowed to settle at 1 *g* for 10 minutes, and the supernatant was removed. Parasitic females from infections of single gerbils were then mixed with 200 μl of TRIzol reagent and snap frozen in liquid nitrogen.
